# Development of Heterocycle-Substituted and Fused Azulenes in the Last Decade (2010–2020)

**DOI:** 10.3390/ijms21197087

**Published:** 2020-09-25

**Authors:** Taku Shoji, Tetsuo Okujima, Shunji Ito

**Affiliations:** 1 Department of Material Science, Graduate School of Science and Technology, Shinshu University, Matsumoto 390-8621, Japan; 2 Department of Chemistry and Biology, Graduate School of Science and Engineering, Ehime University, Matsuyama 790-8577, Japan; 3 Graduate School of Science and Technology, Hirosaki University, Hirosaki 036-8561, Japan

**Keywords:** azulene, heterocycles, synthetic method, reactivity

## Abstract

Azulene derivatives with heterocyclic moieties in the molecule have been synthesized for applications in materials science by taking advantage of their unique properties. These derivatives have been prepared by various methods, involving electrophilic substitution, condensation, cyclization, and transition metal-catalyzed cross-coupling reactions. Herein, we present the development of the synthetic methods, reactivities, and physical properties for the heterocycle-substituted and heterocycle-fused azulenes reported in the last decade.

## 1. Introduction

**Scheme 1:**
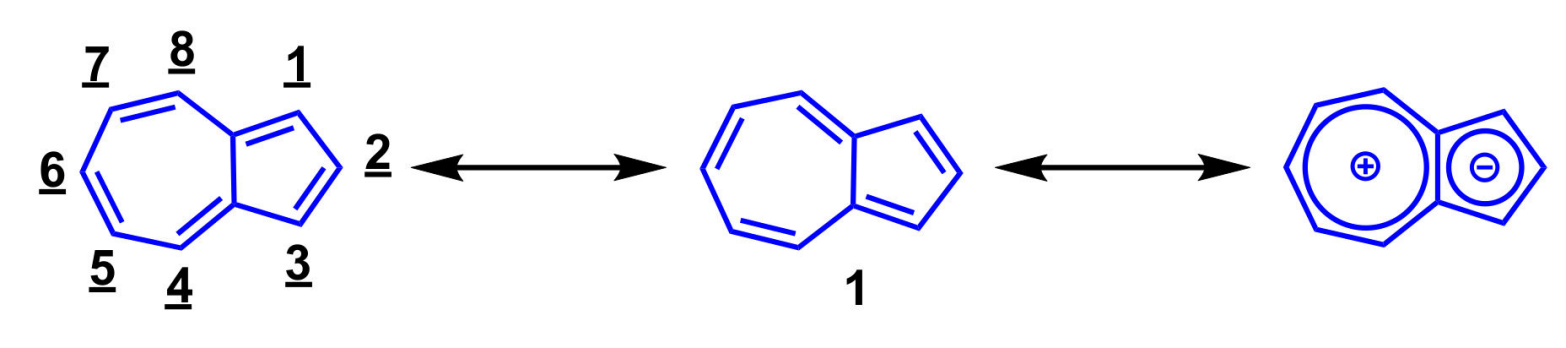
Resonance structure and numbering scheme of azulene (**1**).

Azulene (**1**) is one of the 10π-electron non-benzenoid aromatic hydrocarbons having a fused structure of 5- and 7-membered rings. Azulene (**1**) is one of the structural isomers of naphthalene, however, the color of these isomers differ greatly from each other. Despite the fact that naphthalene is a colorless compound, azulene (**1**) shows a beautiful blue color. The name “azulene” derives from the Arabic word “azur” and the Spanish word “azul” which both mean “blue”. Azulene (**1**) has the contribution of the cyclopentadienide and tropylium substructures as shown in Scheme 1. Hence, despite being a hydrocarbon, azulene (**1**) shows a relatively large dipole moment (1.08 debye). Furthermore, electrophilic substitution reaction of azulene (**1**) proceeds at 1-, 3-, 5-, and 7-positions, while nucleophilic addition reaction takes place at 4-, 6-, and 8-positions. Some azulene derivatives have pharmacological activity, and azulene sulfonic acid derivatives are especially used as anti-inflammatory drugs [1,2,3]. In recent years, azulene derivatives have also been reported to exhibit anti-tumor activity [4,5,6,7].

Due to their unique optical and electrochemical properties, heterocycle-substituted and heterocycle-fused azulene derivatives are expected to be applied to organic materials such as organic light-emitting diodes (OLEDs), organic field effect transistors (OFETs), solar cells, and biosensors. Therefore, many researchers have studied the synthesis, reactivity, and properties of these derivatives, as discussed below. Several review articles on the classical heterocycle-substituted and heterocycle-fused azulene derivatives have been published so far [8,9,10,11,12,13,14,15,16,17]. However, there is no systematic review of the progress in the synthesis and reactivity of these derivatives within the last decade. Herein, we describe the development of heterocycle-substituted and heterocycle-fused azulene derivatives in the last decade.

## 2. Synthesis and Reactivity of Azulene Derivatives with 6-Membered Ring Heterocycles

### 2.1. Azulene Derivatives with N-Containing 6-Membered Heterocycles

Nitrogen-containing (N-containing) 6-membered heterocycles, such as pyridine, are an important substructure found in pharmaceuticals and natural products. Therefore, the synthesis and reactivity of these derivatives with N-containing 6-membered heterocycles have been reported in relatively large numbers.

**Scheme 2:**
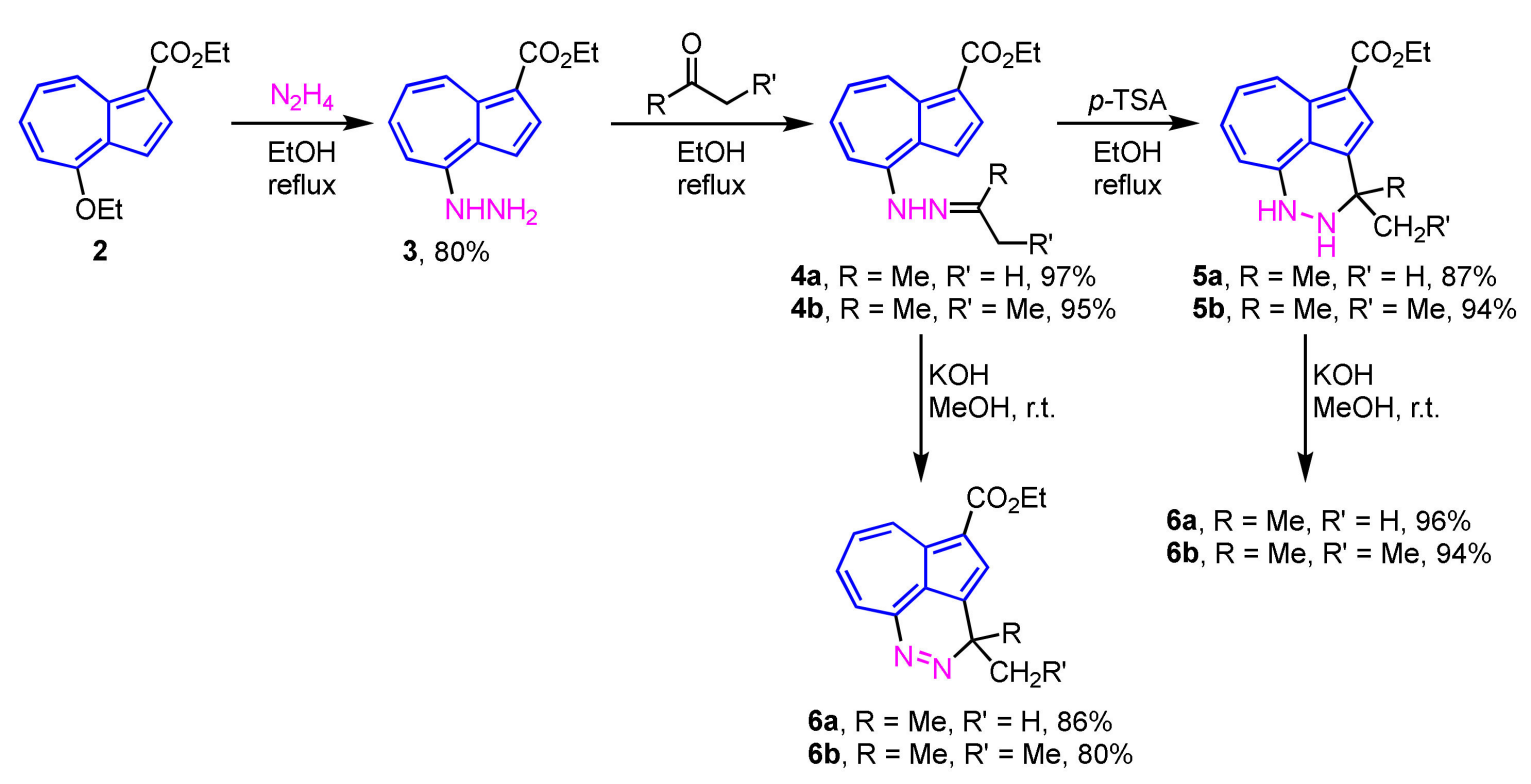
Syntheses of azulenopyridazines **6a** and **6b** from 4-hydrazinylazulene **3**.

In 2010, Wu et al. reported the two- or three-step synthesis of azulenopyridazines 6a and 6b from 4-hydrazinylazulene 3, which were prepared by the S_N_Ar reaction of 4-ethoxyazulene 2 with hydrazine (N_2_H_4_) [18]. The reaction of 3 with ketones in EtOH gives the corresponding hydrazones 4a and 4b in excellent yields. When the hydrazones 4a and 4b are treated with *p*-toluenesulfonic acid (*p*-TSA), dihydropyridazines 5a and 5b can be obtained in 87% and 94% yields, respectively. Both hydrazones 4a, 4b and dihydropyridazines 5a, 5b afford the corresponding azulenopyridazines 6a and 6b by the treatment with KOH in MeOH in excellent yields (Scheme 2).

**Scheme 3:**
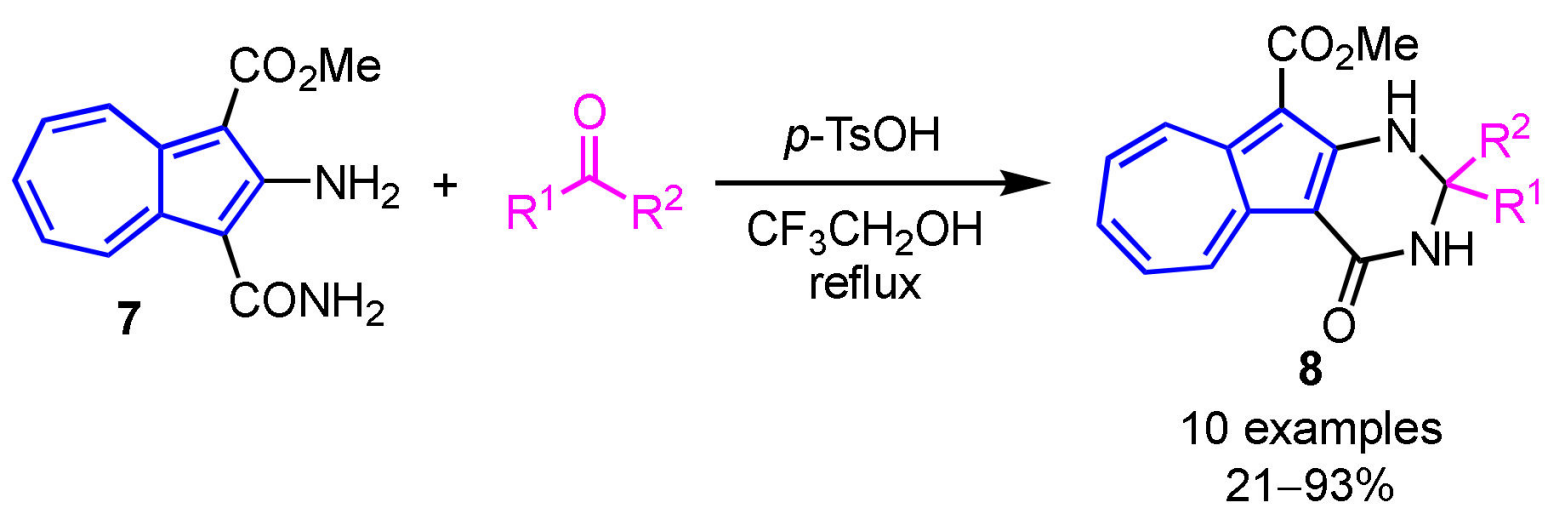
Synthesis of 2,3-dihydro-4(1*H*)-azuleno[2,1-*d*]pyrimidinones **8** by the condensation of 2-amino-3-carbamoylazulene **7** with carbonyl compounds.

A facile synthesis of 2-substituted 2,3-dihydro-4(1*H*)-azuleno[2,1-*d*]pyrimidinones **8** was demonstrated by Wang and co-workers (Scheme 3) [19]. In their procedure, Brønsted acid-catalyzed reaction of 2-amino-3-carbamoylazulene **7** with ketones or aldehydes in 2,2,2-trifluoroethanol gives the azulene-fused dihydropyrimidinones **8** in moderate to excellent yields.

**Scheme 4:**
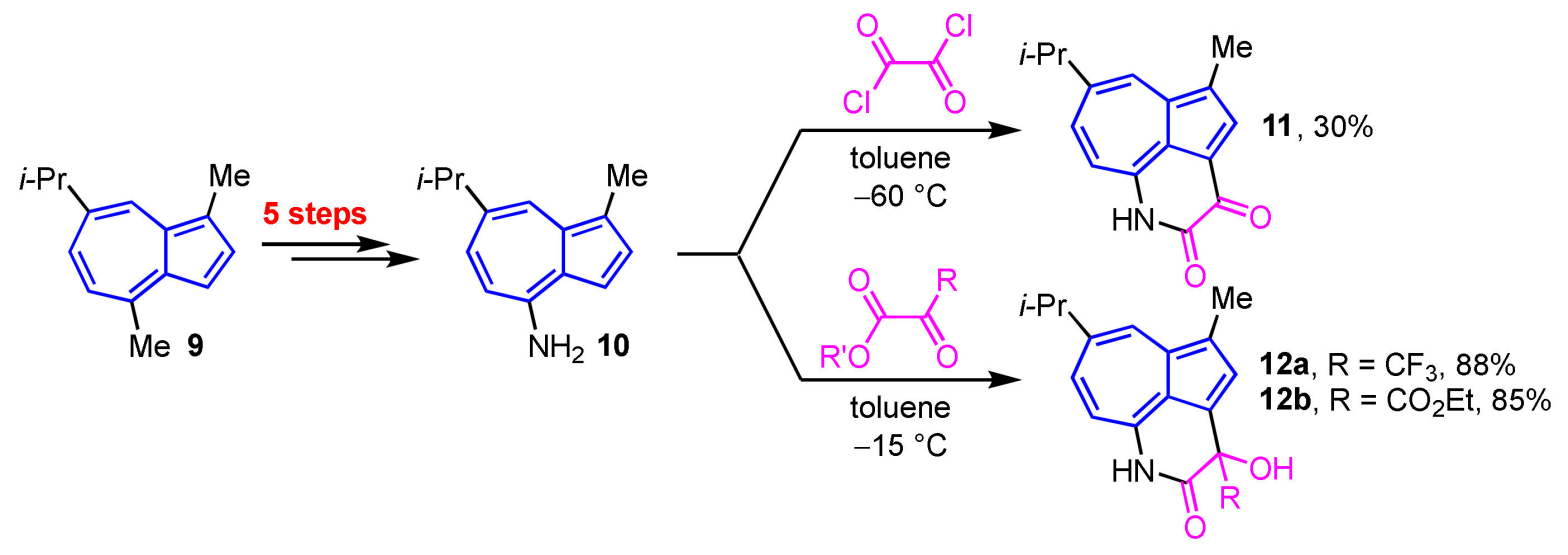
Synthesis of azulene-fused *δ*-lactams **11**, **12a** and **12b** from 4-aminoguaiazulene (**10**).

Yli-Kauhaluoma et al. reported the synthesis of azulene-fused *δ*-lactams **11**, **12a**, and **12b** by the sequential nucleophilic and intramolecular cyclization reactions of 4-aminoazulene (**10**), which was prepared from guaiazulene (**9**) in five steps, including Curtius–Schmidt rearrangement, with the corresponding 1,2-diketones (Scheme 4) [20].

**Scheme 5:**
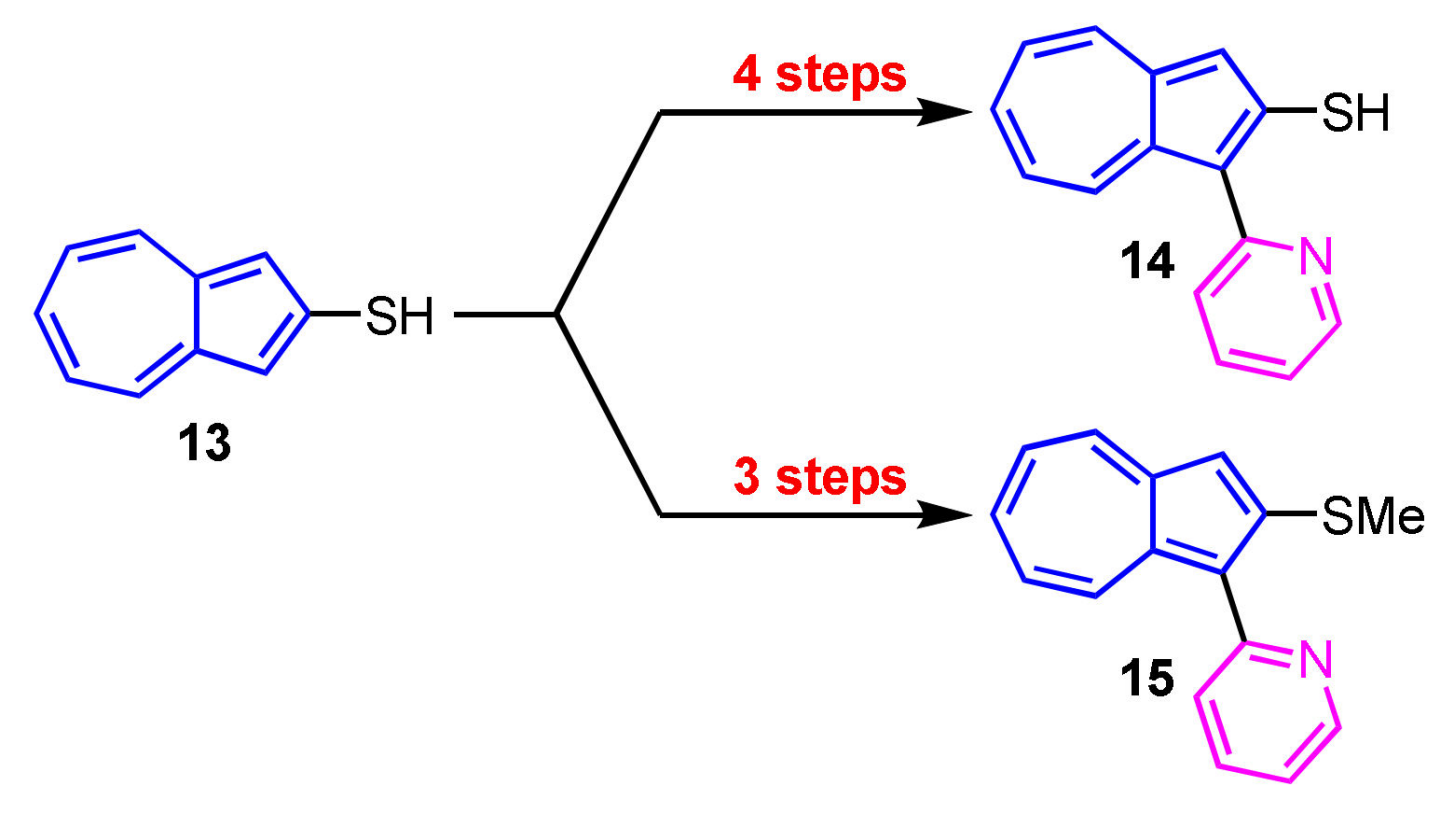
Synthesis of 1-(2-pyridyl)azulenes **14** and **15** with a sulfur function at the 2-position.

Synthesis of 1-(2-pyridyl)azulenes **14** and **15** bearing a thiol and a methylthio group at the 2-position was reported by Wakabayashi and his colleagues (Scheme 5) [21]. They investigated the metal ion sensitivity of these compounds and found that **14** and **15** show color change with addition of Hg^2+^ ion.

**Scheme 6:**
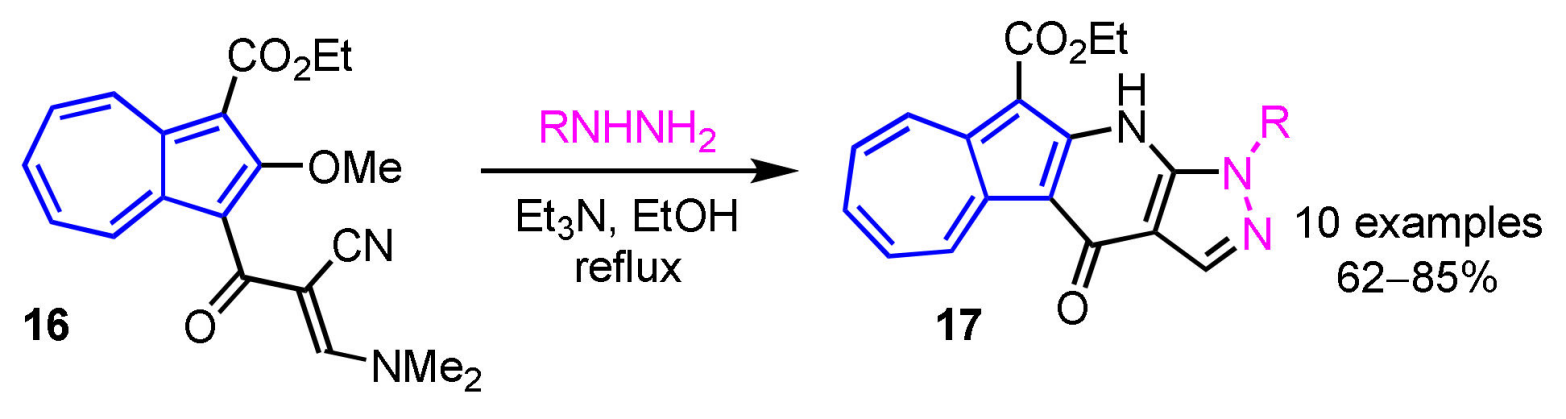
Synthesis of 11*H*(2*H*)-4-oxopyrazolo[3′,4′:6,5]pyrido[3,2-*a*]azulenes **17** by the domino reaction.

**Scheme 7:**
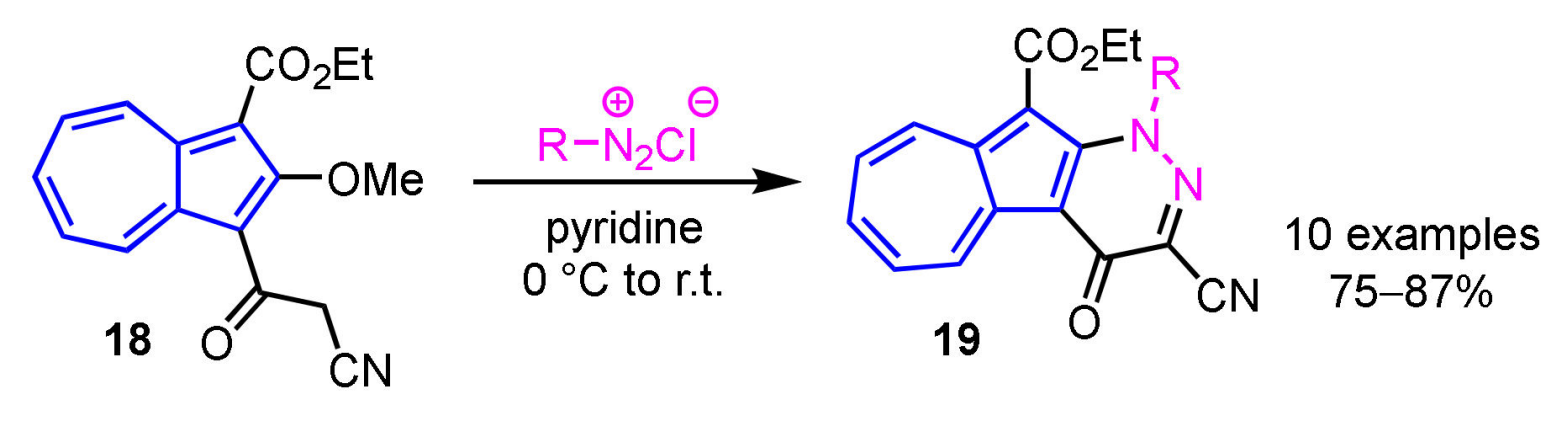
Synthesis of azuleno[2,1-*b*]pyridazines **19** from 1-cyanoacetyl-2-methoxyazulene **18**.

In 2013, Wang et al. developed a preparation method of 11*H*(2*H*)-4-oxopyrazolo[3′,4′:6,5]pyrido[3,2-*a*]azulene derivatives **17** by the domino reaction of **16** with hydrazines in moderate to good yields (Scheme 6) [22]. A simple procedure for azuleno[2,1-*b*]pyridazines **19** was also reported by the same group. In this reaction, azuleno[2,1-*b*]pyridazines **19** are obtained by the condensation between 1-cyanoacetyl-2-methoxyazulene **18** and aryldiazonium salts in pyridine in good yields (Scheme 7) [23].

**Scheme 8:**
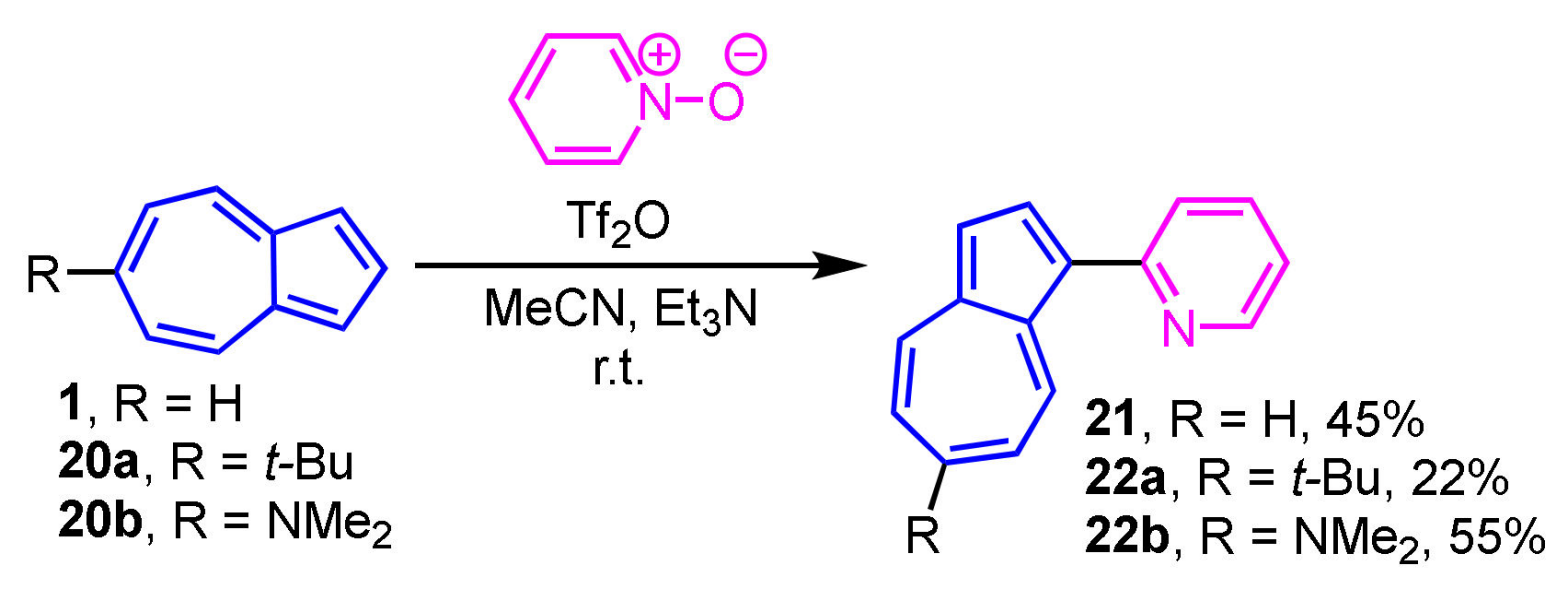
Synthesis of 1-(2-pyridyl)azulenes **21**, **21a**, and **22b**.

The Reissert–Henze-type reaction is applicable to the synthesis of 1-(2-pyridyl)azulene (**21**) and its derivatives **22a** and **22b**. The reaction of **1**, **20a** and **20b** with pyridine *N*-oxide in the presence of trifluoromethanesulfonic anhydride (Tf_2_O) yields **21**, **22a** and **22b** in moderate to good yields (Scheme 8) [24]. This procedure is also applicable to the substituted pyridine *N*-oxides and quinoline *N*-oxide, which react with azulenes to produce the corresponding 1-(2-pyridyl- and 2-quinolyl)azulenes.

**Scheme 9:**
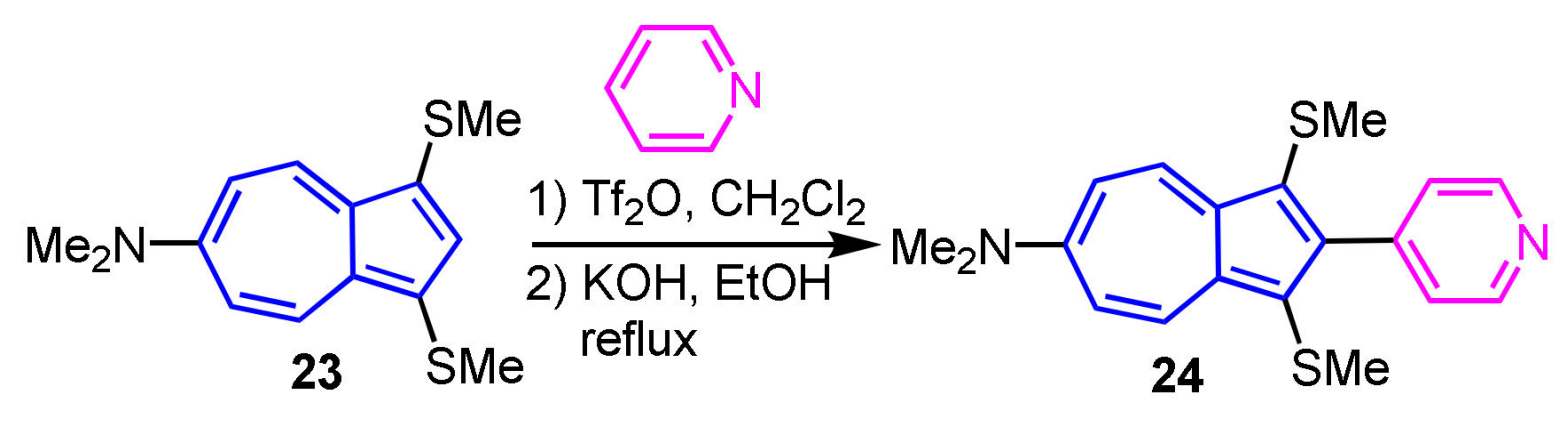
Synthesis of 2-(4-pyridyl)azulene derivative **24** via electrophilic substitution.

The synthesis of 2-heteroarylazulenes via electrophilic substitution reaction was first described in 2012. The reaction of 6-dimethylamino-1,3-bis(methylthio)azulene (**23**) with pyridine in the presence of Tf_2_O, followed by treatment with KOH, gives 2-(4-pyridyl)azulene **24** (Scheme 9) [25]. In a similar reaction, it is possible to employ quinoline, acridine, and 1,10-phenanthroline to furnish the corresponding 2-heteroarylazulene derivatives. This method is the first example of the synthesis of 2-heteroarylazulenes via an electrophilic substitution reaction.

**Scheme 10:**
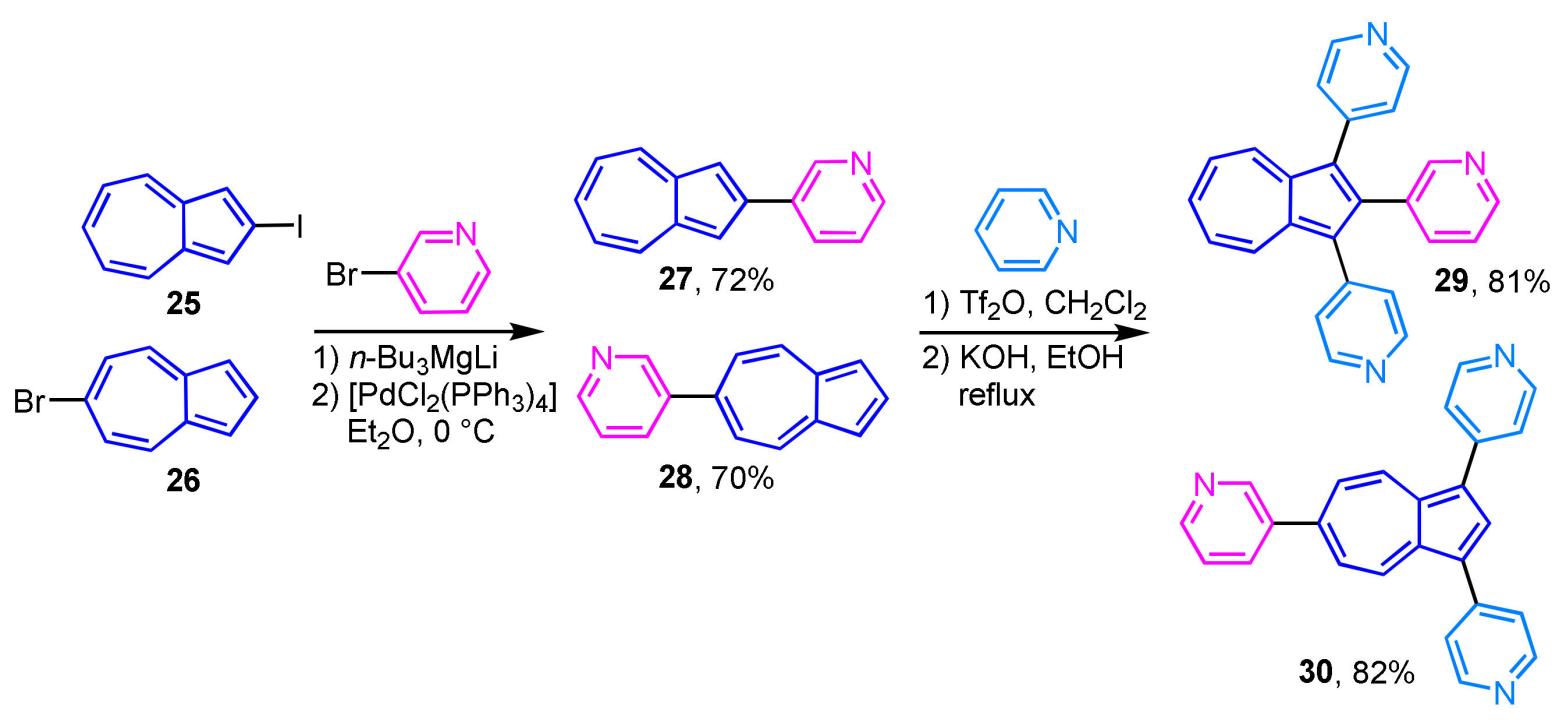
Synthesis and pyridinylation of 2- and 6-(3-pyridyl)azulenes **27** and **28**.

The palladium-catalyzed cross-coupling reaction of 2-iodoazulene (**25**) and 6-bromoazulene (**26**) with lithium pyridyl and quinolyl magnesium ate-complexes affords the corresponding 2- and 6-heteroarylazulene derivatives **27** and **28** in good to excellent yields (Scheme 10) [26]. The reaction of **27** and **28** with pyridine in the presence of Tf_2_O and subsequent aromatization with KOH gives the corresponding tris(heteroaryl)azulenes **29** and **30**.

**Scheme 11:**
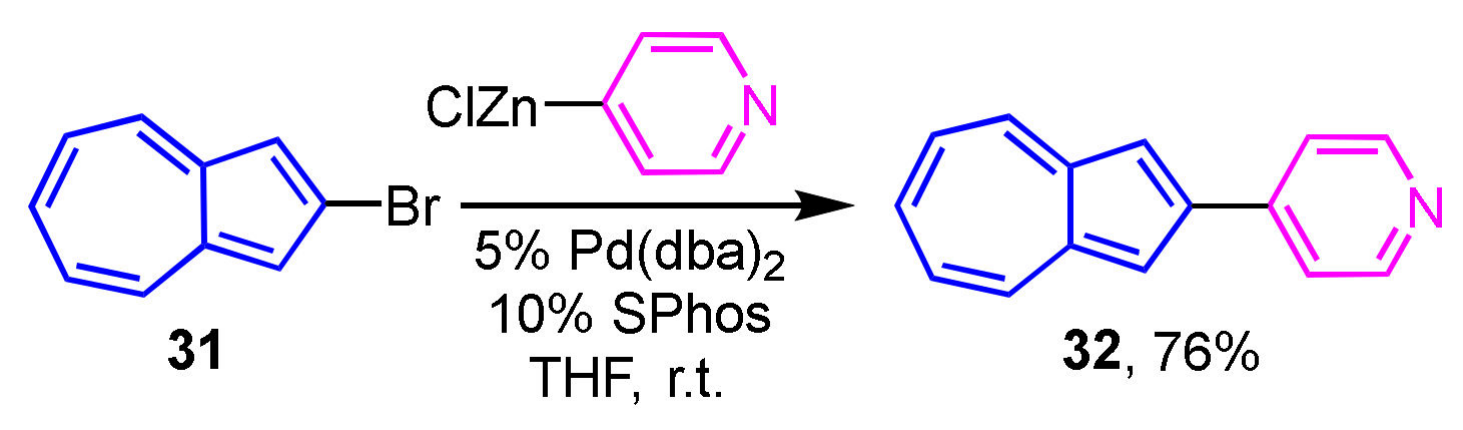
Synthesis of 2-(4-pyridyl)azulene (**32**) using a heteroaryl zinc reagent.

**Scheme 12:**
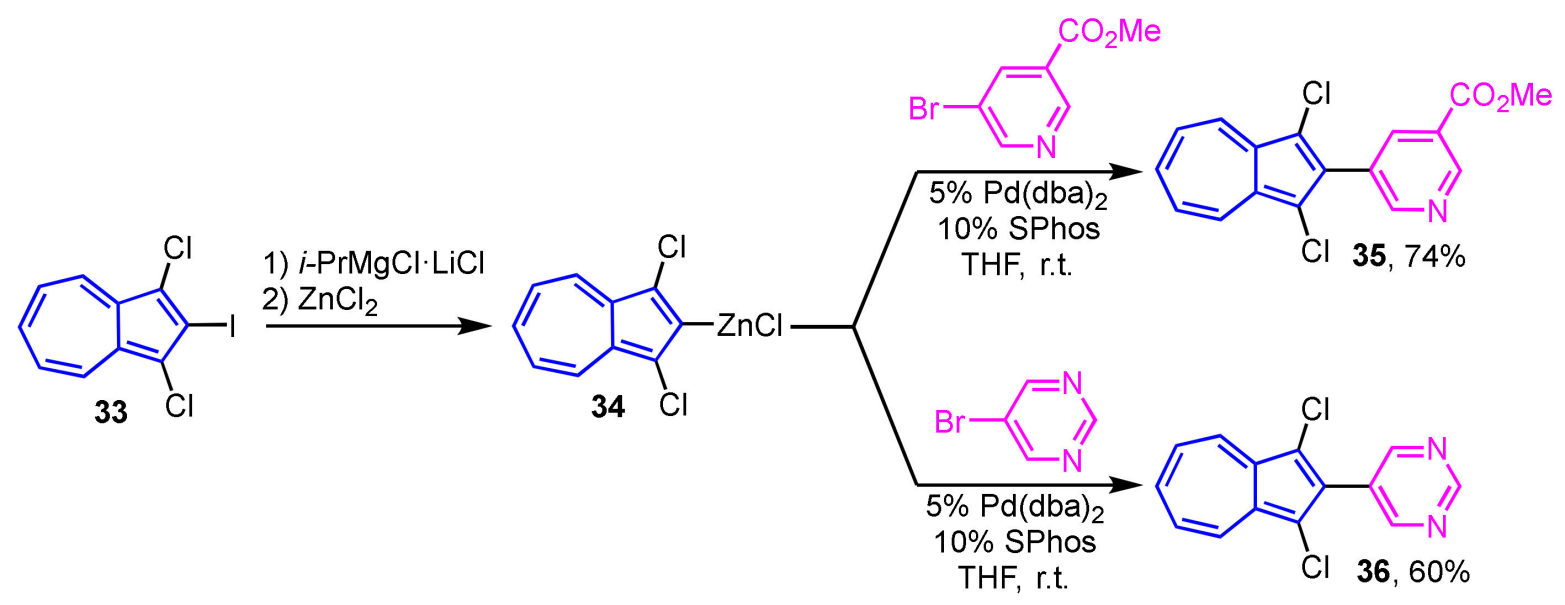
Synthesis of 2-(pyridyl- and pyrimidiyl)azulenes **35** and **36** by Negishi cross-coupling reaction using 2-azulenyl zinc reagent **34**.

Preparation of 2-heteroarylazulenes by the Negishi cross-coupling reaction was achieved in two ways by Bredihhin et al. The first is the reaction of 2-bromoazulene (**31**) with a zinc reagent of the heterocycles, where the cross-coupling reaction of **31** with 4-pyridyl zinc reagent at room temperature yields 2-(4-pyridyl)azulene (**32**) in 76% yield (Scheme 11) [27]. In another way, 2-azulenyl zinc reagent **34**, which is prepared from 2-iodoazulene derivative **33**, reacts with heteroaryl bromides at room temperature to give 2-(pyridyl- and primidinyl)azulene derivatives **35** and **36** in good yields. In this case, the 2-azulenyl zinc reagent **34** can be prepared by the direct metalation of 1,3-dichloro-2-iodoazulene (**33**) precursor with *i*-PrMgCl·LiCl, then transmetalation with zinc chloride (Scheme 12) [28].

**Scheme 13:**
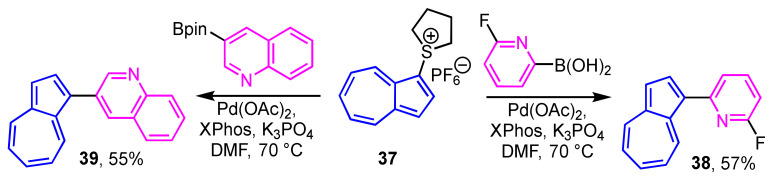
Synthesis of 1-(2-pyridyl)- and 1-(3-quinolyl)azulenes **38** and **39** from 1-azulenylsulfonium salt **37**.

1-Haloazulenes, such as 1-bromo- and 1-iodoazulenes, are known to be unstable compounds. Lewis and co-workers demonstrated the preparation of 1-heteroarylazulenes by the cross-coupling reaction of aryl boronic acids with 1-azulenylsulfonium salt **37**, instead of the 1-haloazulenes [29]. In this procedure, the reaction of the 1-azulenylsulfonium salt **37** with the boronic acids or acid esters of the heterocycles in the presence of a palladium/XPhos catalyst affords the corresponding 1-heteroarylazulenes **38** and **39** (Scheme 13). This method is also applicable to the synthesis of 1-(2-thienyl)azulenes. Details of the synthesis of these derivatives are described later.

**Scheme 14:**
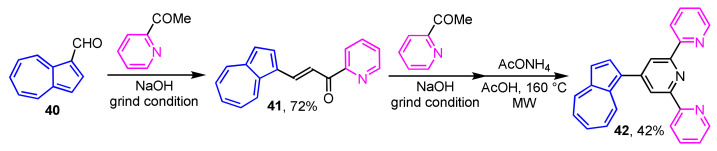
Preparation of 2,2′:6′,2”-terpyridine-substituted azulene **42**.

In 2016, Nica et al. reported the multistep synthesis of 2,2′:6′,2′′-terpyridine-substituted azulene derivatives under the Kröhnke-type reaction conditions (Scheme 14) [30]. Condensation of **40** with 2-acetylpyridine under grinding conditions leads to the corresponding azulene-substituted chalcone **41** in 72% yield. Compound **41** is subjected to further reaction with 2-acetylpyridine under the same conditions, then the microwave (MW) irradiation in the presence of ammonium acetate to form 4-(1-azulenyl)terpyridine **42** in 42% yield.

**Scheme 15:**
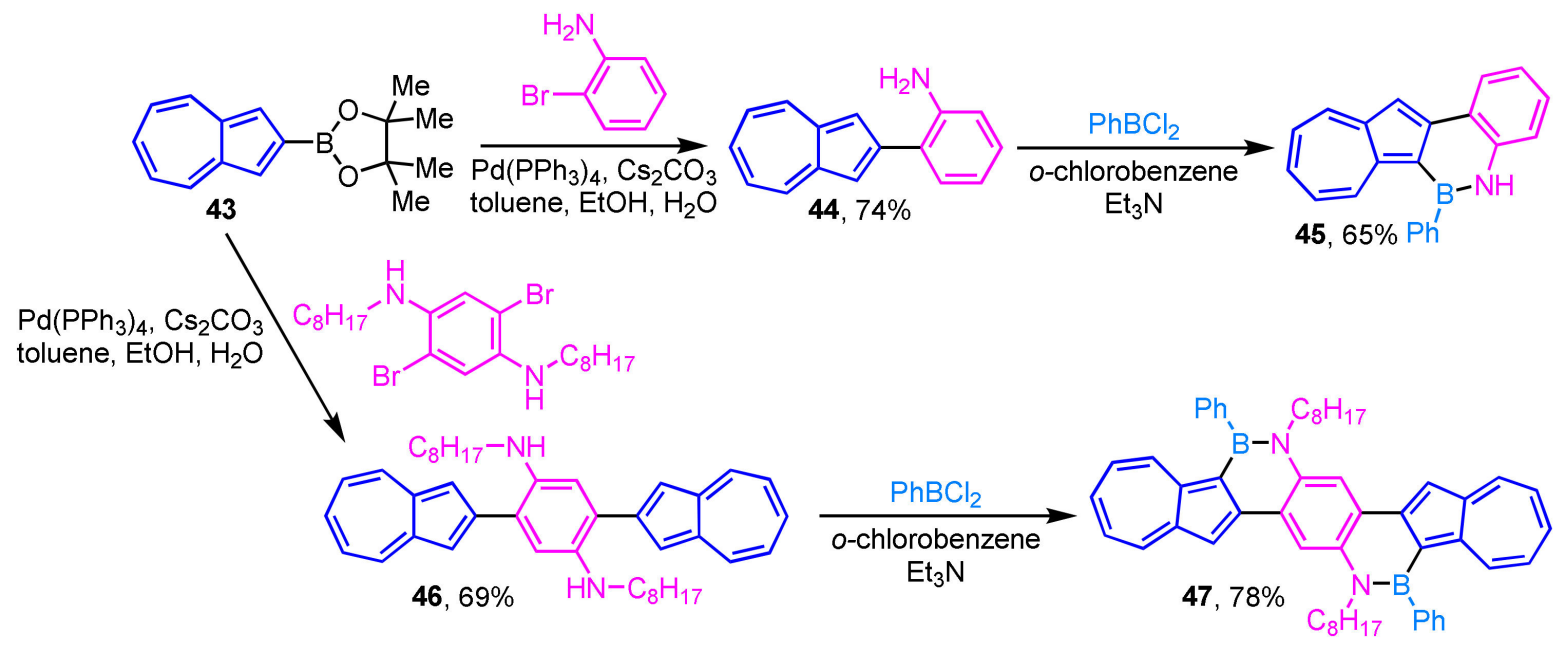
Synthesis of azulene-fused BN-heterocycles **45** and **47** via aromatic borylation.

The polycyclic aromatic hydrocarbons (PAHs) in which a part of the C=C moiety is replaced with a B–N unit has attracted considerable attention in the field of materials science. Recently, Gao et al. reported the preparation of two azulene-fused BN-heterocycles **45** and **47** via aromatic borylation of **44** and **46** (Scheme 15) [31]. Compounds **45** and **47** exhibit unique absorption and fluorescence properties unlike those of previously reported BN-embedded PAHs. Furthermore, **45** and **47** show high sensitivity toward fluoride ion, and a remarkable color change is observed by the addition of the ion.

### 2.2. Azulene Derivatives with O-Containing 6-Membered Heterocycles

Several examples of azulene derivatives, which are substituted or fused by oxygen-containing (O-containing) 6-membered heterocycles, have been reported. The synthesis of these derivatives is mainly established by the intermolecular or intramolecular cyclization reactions.

**Scheme 16:**
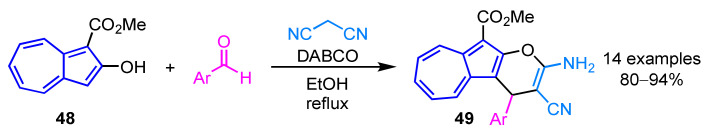
Synthesis of 2-amino-3-cyano-4-aryl-10-ethoxycarbonylazuleno[2,1-*b*]pyrans **49** from 2-hydroxyazulene derivative **48**.

In 2012, Wang et al. reported the synthesis of 2-amino-3-cyano-4-aryl-10-ethoxycarbonylazuleno[2,1-*b*]pyrans **49** from 2-hydroxyazulene derivative **48** (Scheme 16) [32]. The three-component cascade reaction of **48**, arylaldehydes and malononitrile in the presence of diazabicyclo[2.2.2]octane (DABCO) as a base produces the corresponding products **49** in excellent yields.

**Scheme 17:**
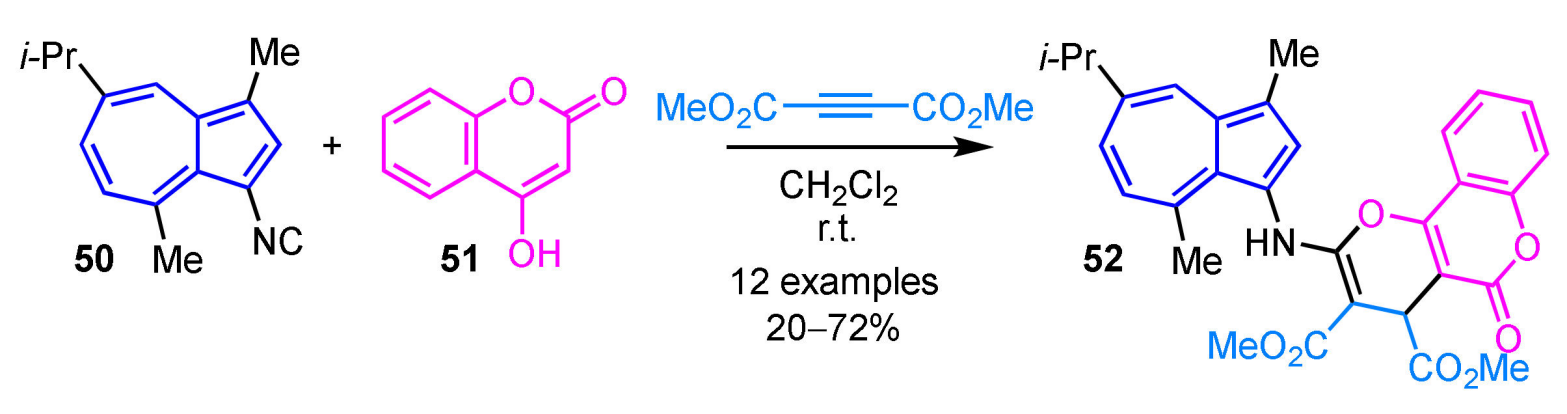
Synthesis of tricyclic compound **52** connected with 3-aminoguaiazulene by the multicomponent reaction.

Sato et al. demonstrated the preparation of tricyclic compound **52** connected with 3-aminoguaiazulene by the multicomponent reaction of 3-isocyanoguaiazulene **50**, dimethyl acetylenedicarboxylate (DMAD), and 4-hydroxycoumarin (**51**) (Scheme 17) [33]. This reaction also allows synthesis of the corresponding furan or hydropyridine derivatives by using an aldehyde or aniline derivatives instead of 4-hydroxycoumarin.

**Scheme 18:**
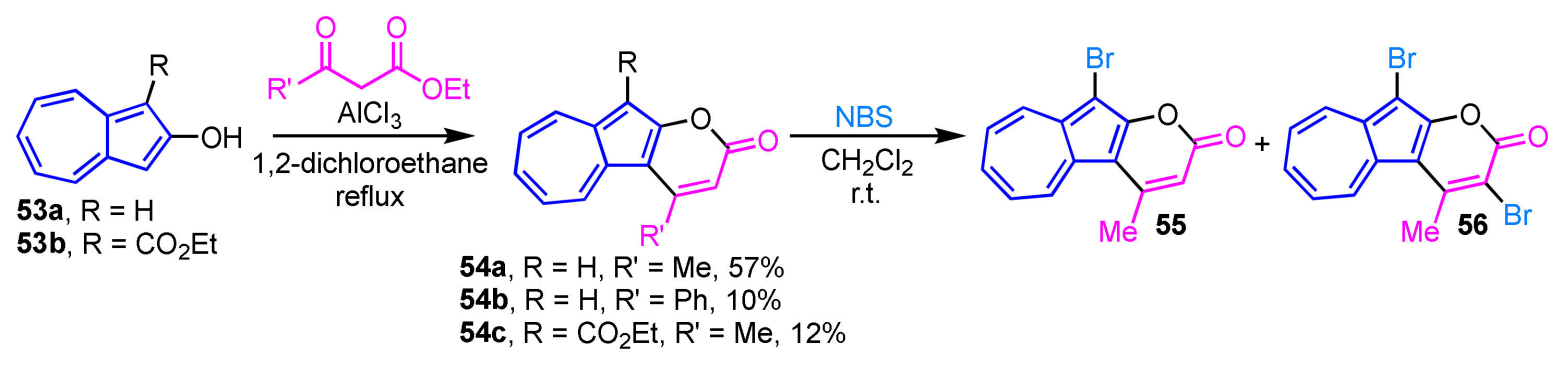
Synthesis of azulenopyranones **54a**–**54c** by AlCl_3_-mediated cyclization reaction.

The AlCl_3_-mediated cyclization reaction of 2-hydroxyazulenes **53a** and **53b** with activated methylene compounds gives pyrone-fused azulene derivatives (azulenopyranones **54a**–**54c**) in low to moderate yields (Scheme 18) [34]. The reaction of **54a** with *N*-bromosuccinimide (NBS) results in conversion into the bromides **55** and **56**. When one equivalent of NBS is used, **55** is obtained in 42% yield along with **56** in 31% yield. While, the dibromo derivative **56** is selectively generated in excellent yield (91%), when two equivalents of NBS is employed. Compounds **55** and **56** can be further functionalized by the palladium-catalyzed cross-coupling reaction.

**Scheme 19:**
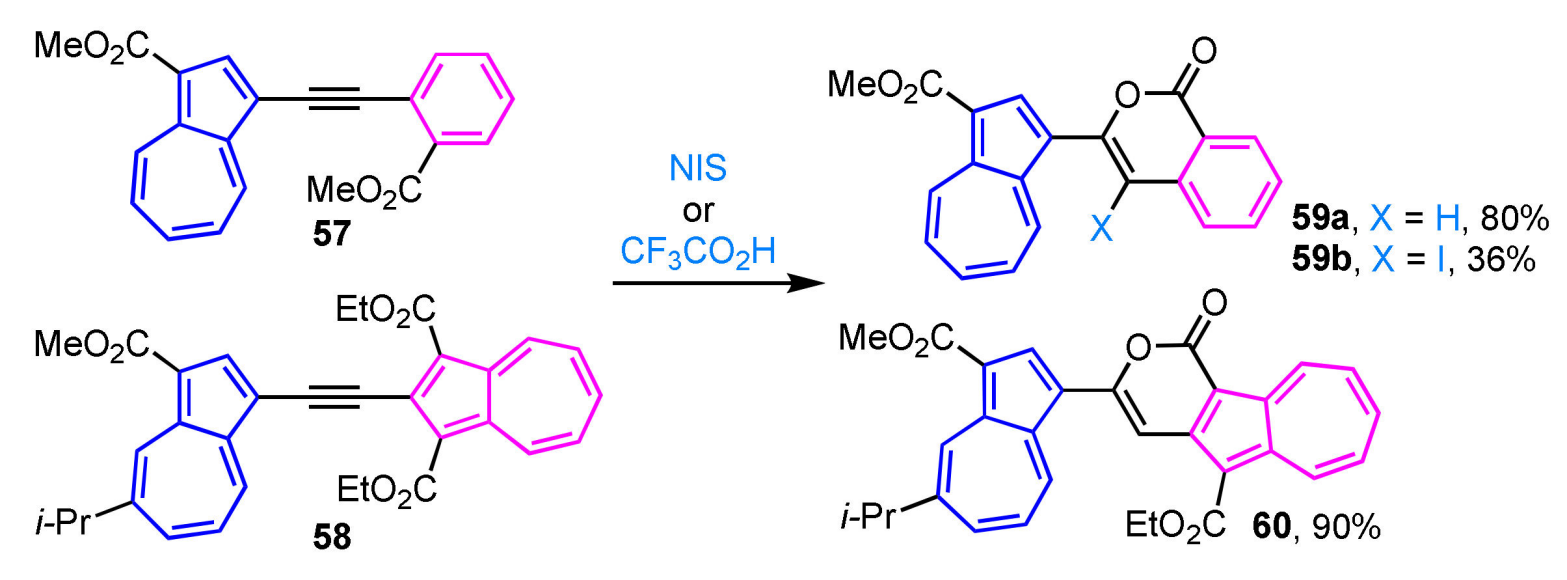
Synthesis of azulene-substituted isocoumarins **59a**, **59b** and pyrone-fused azulene **60** by intramolecular cyclization of 1-azulenyl alkynes.

Intramolecular cyclization of alkyne **57** in the presence of trifluoroacetic acid produces isocoumarin derivative **59a** in excellent yield (Scheme 19) [35], whereas iodocyclization of alkyne **57** with *N*-iodosuccinimide (NIS) gives the corresponding 4-iodoisocoumarin **59b** in moderate yield. Similarly, alkyne **58** having two azulenyl moieties is treated with trifluoroacetic acid in a mixed solvent of THF and water to give pyrone-fused azulene **60** in 90% yield [36].

## 3. Synthesis and Reactivity of Azulene Derivatives with 4-, 5-, 6-, and 7-Membered Ring Heterocycles

### 3.1. Azulene Derivatives with S-Containing 4-, 5-, 6-, and 7-Membered Heterocycles

Thiophene, TTF, and the other sulfur-containing heterocycles are expected to be applied in the field of organic electronic materials such as organic semiconductors. For this reason, many efforts have been focused on the incorporation of such heterocycles into azulene derivatives by cross-coupling, cycloaddition, and condensation reactions.

**Scheme 20:**
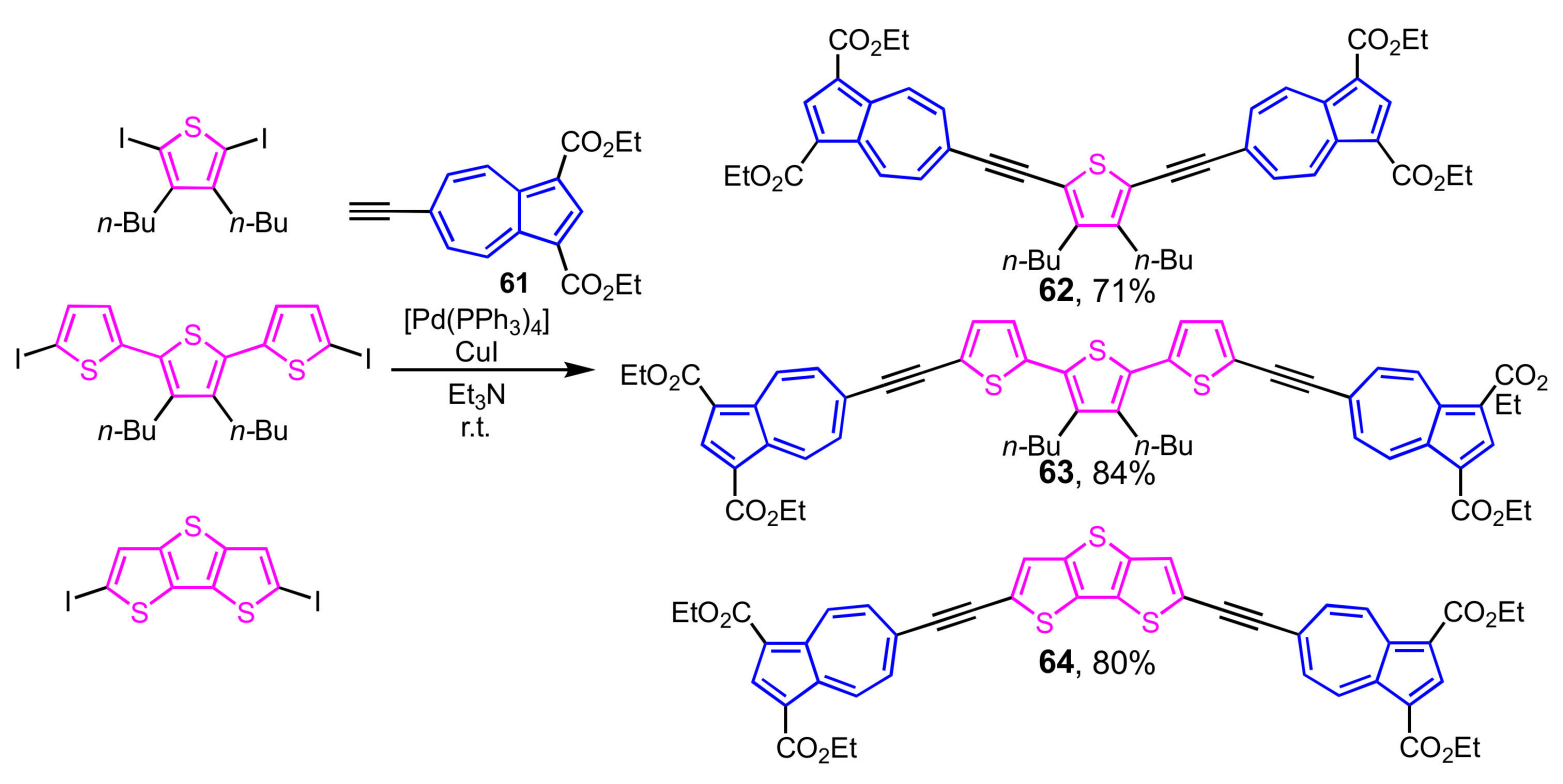
Synthesis of bis(6-azulenylethynyl)thiophenes **62**–**64** by Sonogashira–Hagihara cross-coupling reaction.

Preparation of bis(6-azulenylethynyl)thiophene **62**, terthiophene **63**, and dithienothiophene **64** is examined by the palladium-catalyzed cross-coupling reaction of 6-ethynylazulene **61** with diiodothiophene and the corresponding diiodides under Sonogashira–Hagihara conditions (Scheme 20) [37]. The absorption band of these compounds in their UV/Vis spectra spreads into the near-infrared region due to the decrement of HOMO–LUMO energy gap basis on the expansion of the π-conjugated system.

**Scheme 21:**
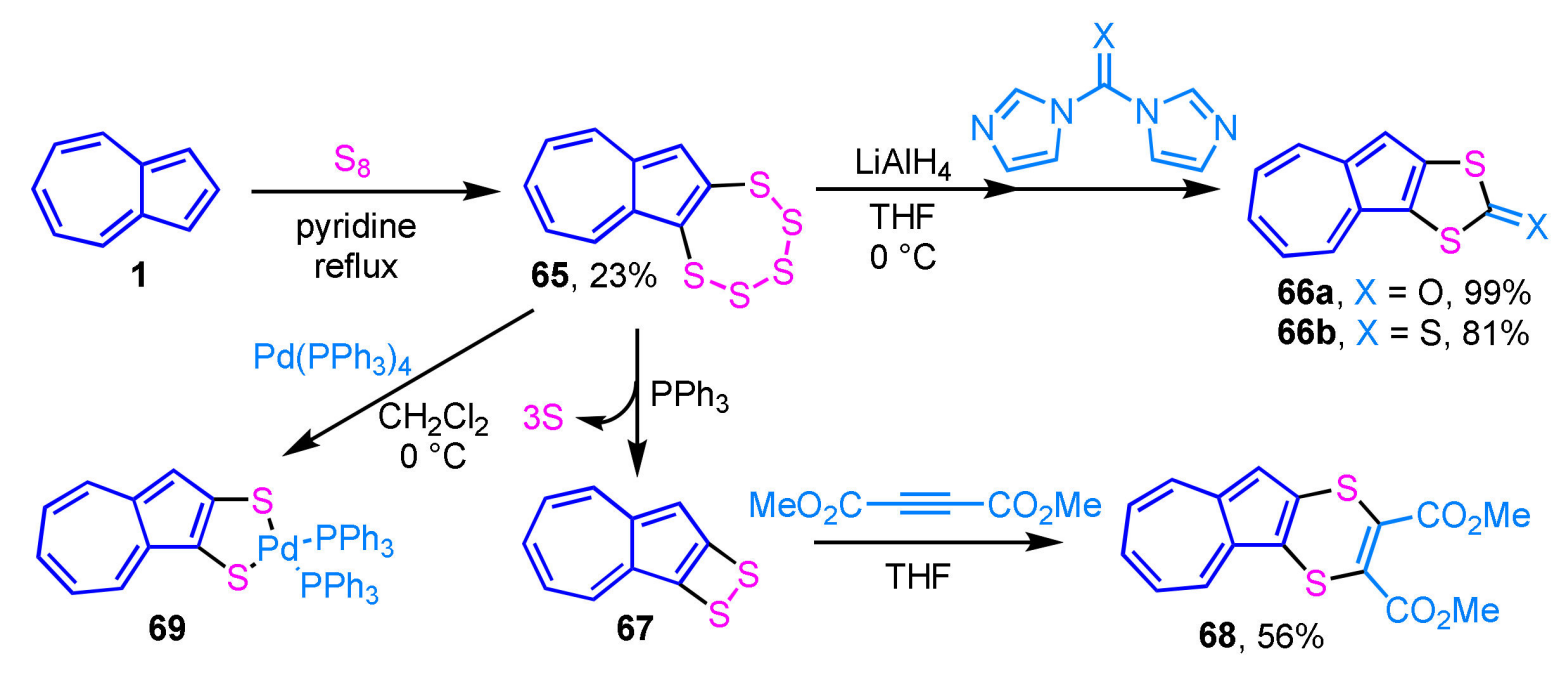
Synthesis and reactivity of azulenopentathiepin **65**.

Synthesis and reactivity of azulenopentathiepin **65** were reported by Sato and his co-workers. Azulenopentathiepin **65** is prepared by the reaction of azulene (**1**) with elemental sulfur in pyridine under reflux conditions. The azulene derivatives, such as **66a** and **66b**, are prepared by the reaction of electrophiles such as iodomethane, methyl chloroformate, *N,N*′-carbonyldiimidazole and *N,N*′-thiocarbonyldiimidazole, with the bis(thiolate) obtained by the treatment of **65** with LiAlH_4_ (Scheme 21) [38]. PPh_3_–mediated desulfuration of pentathiepin **65**, followed by the reaction with DMAD produces azuleno-1,4-dithiin **68** in 56% yield. The reaction of **65** with Pd(PPh_3_)_4_ gives the extremely unstable palladium complex **69** (6%), which is immediately converted to a binuclear complex.

**Scheme 22:**
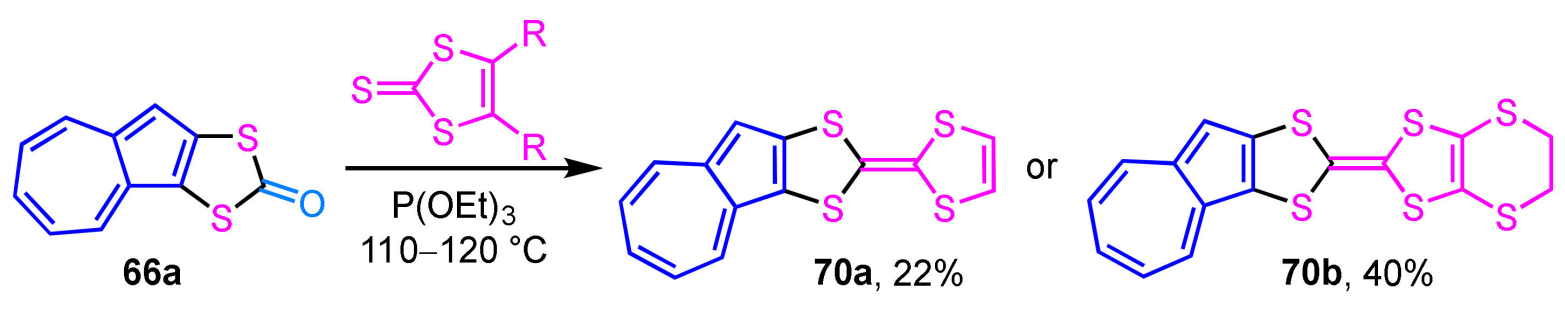
Synthesis of azulene-fused TTFs **70a** and **70b** by the condensation reaction.

Starting from the dithiocarbonate derivative **66a**, which was obtained by the reaction described above, the synthesis of azulene-fused TTFs **70a** and **70b** was achieved by the same group (Scheme 22) [39]. The condensation reaction of dithiocarbonate **66a** with vinylene trithiocarbonate or ethylenedithio derivative in triethyl phosphite generates azulene-fused TTFs **70a** and **70b** in moderate yields (22% and 49%, respectively). The redox behavior of the azulene-fused TTFs was also investigated by cyclic voltammetry (CV).

**Scheme 23:**
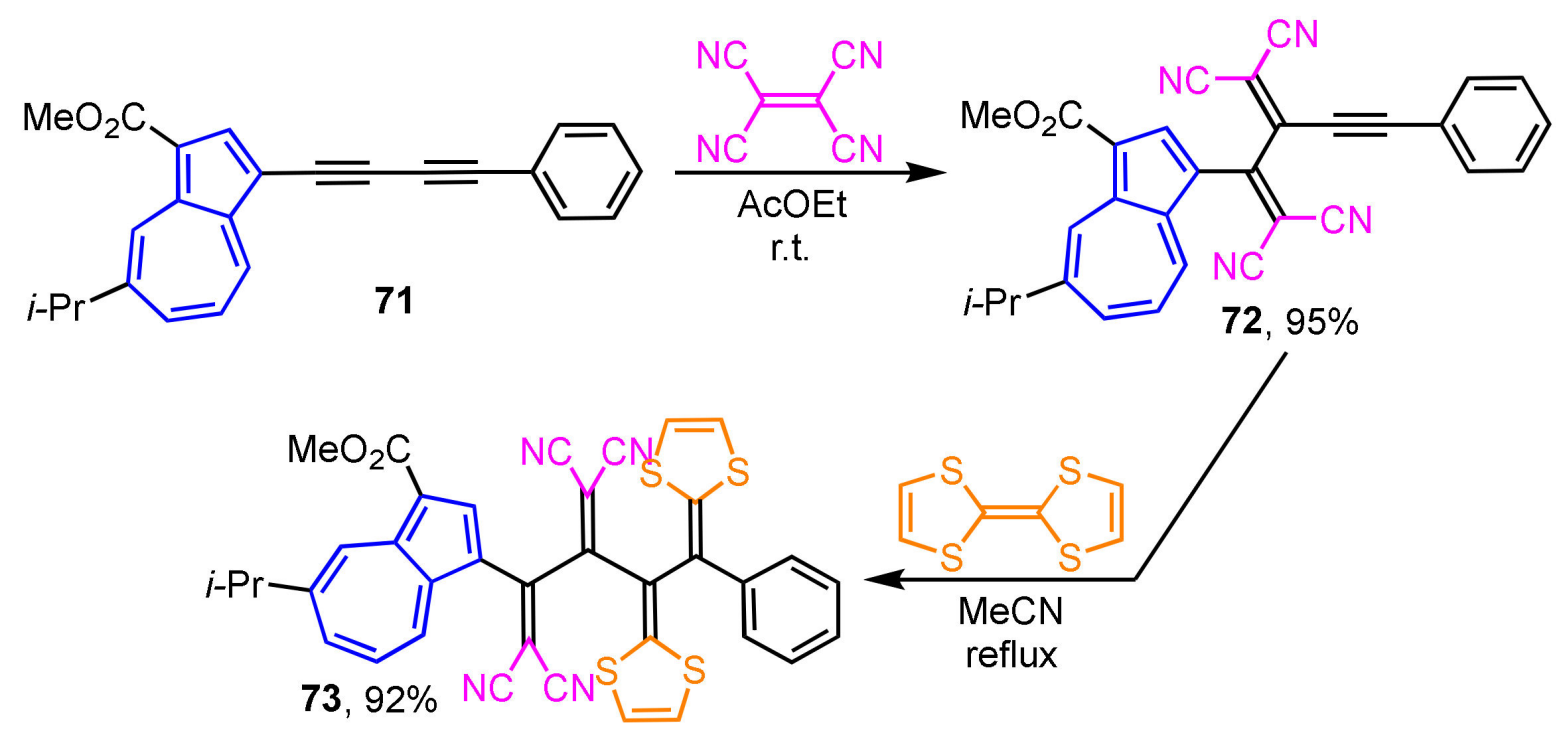
The synthesis of TCNE/TTF double adduct **73** by sequential [2 + 2] cycloaddition–retroelectrocyclization reaction.

Synthesis of the donor–acceptor-type extended TTFs **73** incorporated into cross-conjugated systems is achieved by the sequential [2 + 2] cycloaddition–retroelectrocyclization reaction of electron-rich butadiyne with tetracyanoethylene followed by TTF (Scheme 23) [40]. Butadiyne **71** gives tetracyanobutadiene **72** by the reaction with tetracyanoethylene, which is then converted to **73** by the cycloaddition with TTF. The UV/Vis spectrum of **73** shows a strong and broad absorption band in the visible region (λ_max_ = 471) owing to the overlapping of the charge-transfer (CT) absorption bands arising from both azulene and 1,2-bis(1,3-dithiol-2-ylidene)ethane donors to the tetracyanobutadiene acceptor unit. In the electrochemical analysis of **73**, a reversible one-stage two-electron oxidation wave and a two-stage reduction wave are observed in CV.

**Scheme 24:**
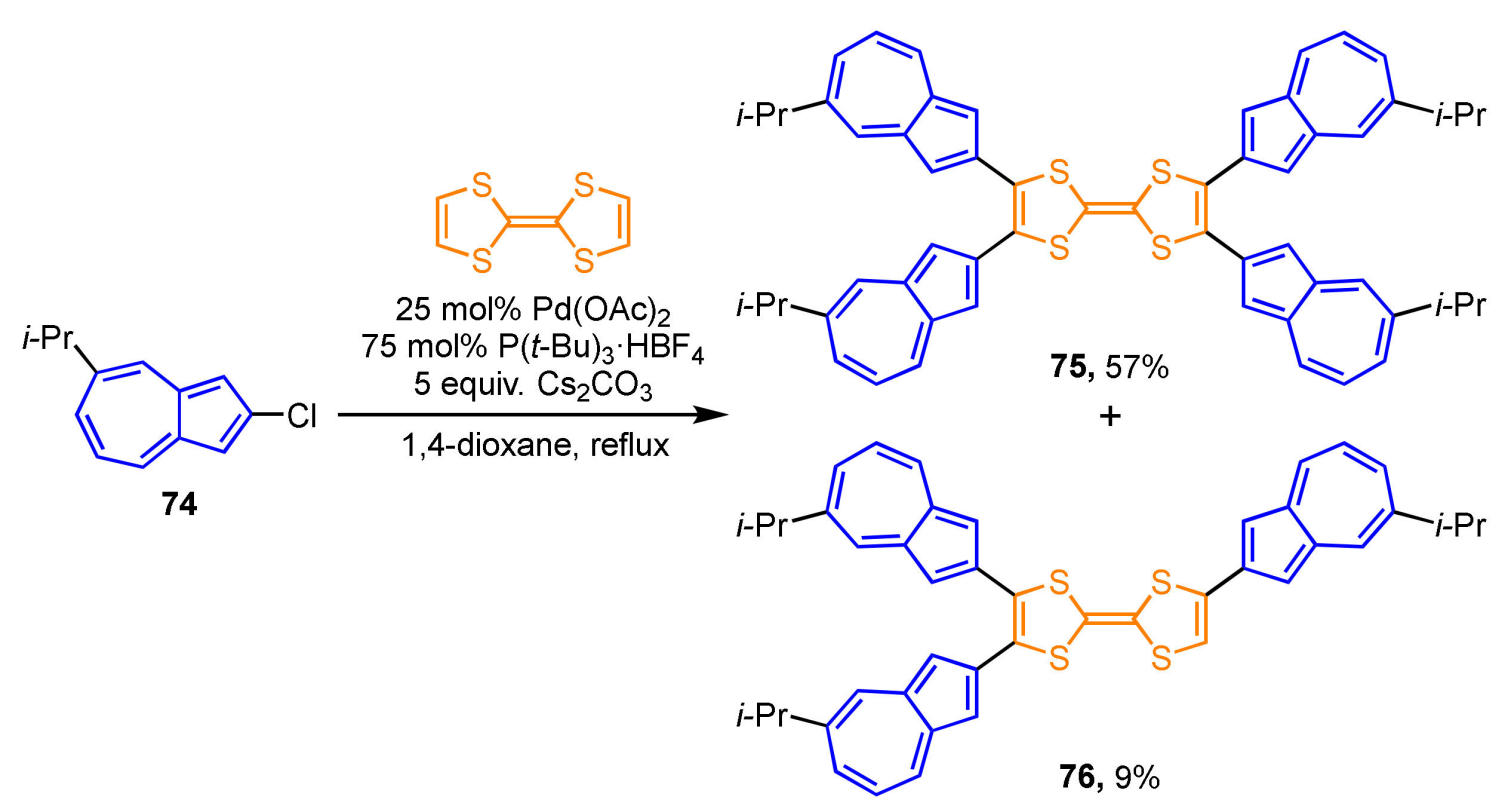
Synthesis of tetra(2-azulenyl)TTF **75** by the palladium-catalyzed C–H bond activation reaction.

Cross-coupling by the palladium-catalyzed C–H bond activation applies to the preparation of azulene-substituted TTFs (Scheme 24). 2-Chloroazulene derivative **74** reacts with TTF in the presence of Pd(OAc)_2_ and P(*t*-Bu)_3_·HBF_4_ as a ligand to generate tetra(2-azulenyl)TTF **75** (57%), along with tri(2-azulenyl)TTF **76** (9%) [41]. Although parent TTF exhibits a two-stage reversible oxidation wave, the CV experiments on **75** display one quasi-reversible wave under the electrochemical oxidation conditions owing to the overlapping of the first wave with the following irreversible waves. The spectroelectrochemical measurements of **75** exhibit significant spectral changes under the electrochemical reduction conditions.

**Scheme 25:**
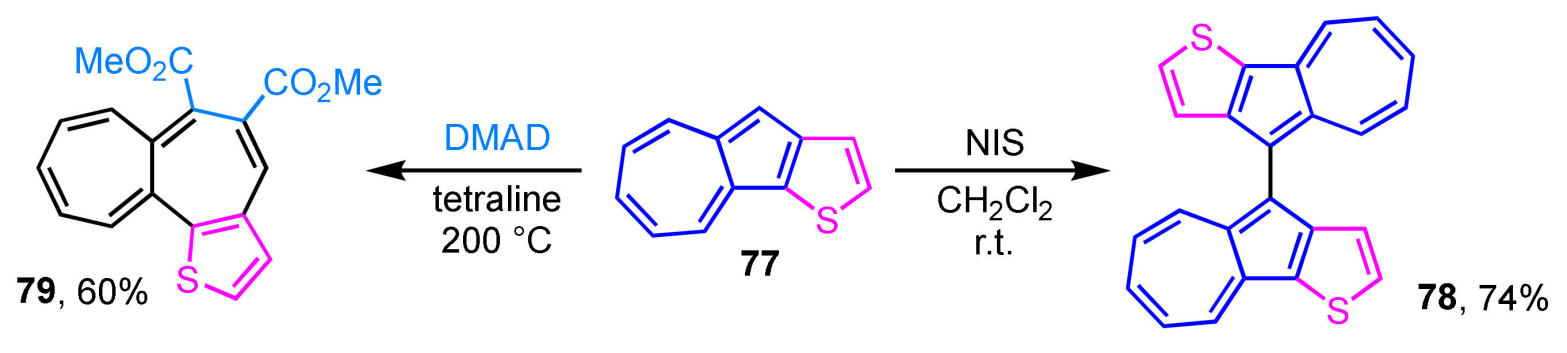
Reactivity of azuleno[1,2-*b*]thiophene (**77**).

Thiophene-fused azulenes sometimes exhibit a different reactivity from that of usual azulene derivatives. Thiophene-fused 1,1′-biazulene derivative **78** is obtained by the reaction of azuleno[1,2-*b*]thiophene (**77**) with NIS, instead of the presumed iodoazulene derivative (Scheme 25) [42]. NIS is commonly known as an iodination reagent, but it may act as an oxidant in this case. Although the longest absorption band of **78** displayed larger absorption coefficients than that of **77**, the absorption maxima are observed at almost the same wavelength region. The similarity of the absorption maxima of **77** and **78** suggests less effective conjugation due to the low planarity of the two azulene rings of **78**. Compound **77** undergoes a sequential cycloaddition–reverse electron cyclization with DMAD under high temperature reaction conditions (200 °C) to give thiophene-fused heptalene **79** in 60% yield [43]. The presence of bond alternation and the nonaromatic nature of **79** are revealed by single-crystal X-ray crystallographic analysis.

**Scheme 26:**
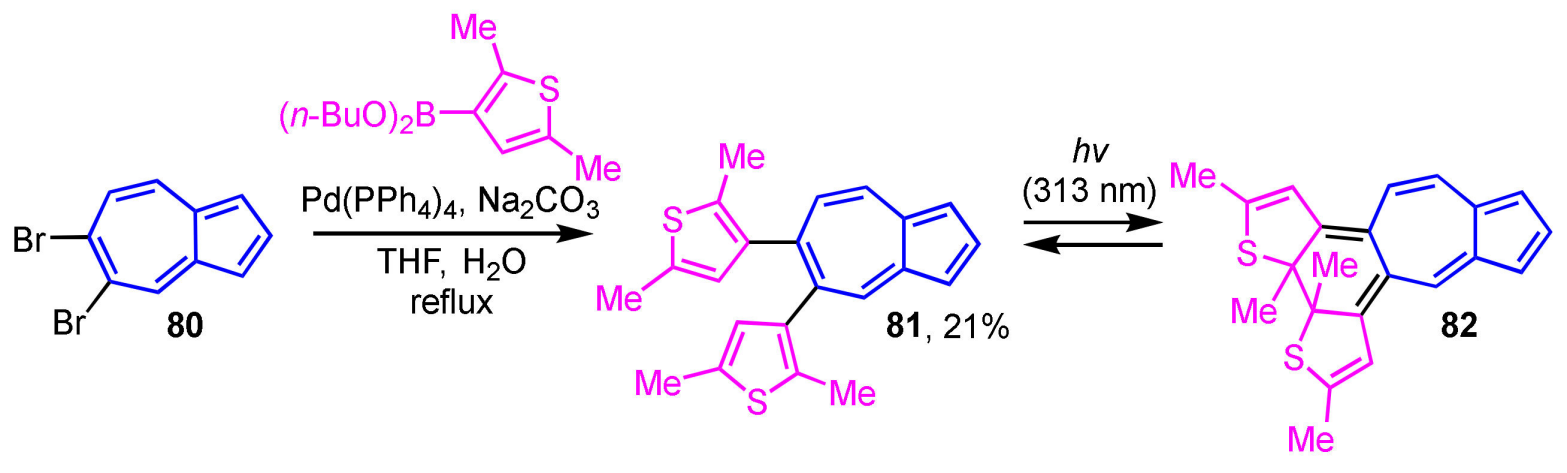
Synthesis of 5,6-di(3-thienyl)azulene derivative **81** by Suzuki–Miyaura cross-coupling reaction.

Photochromism is a phenomenon that is attracting a lot of attention in the field of materials science. Construction of an azulene-based photochromic system was reported by Uchida and co-workers (Scheme 26) [44]. The synthesis of **81** is accomplished by Suzuki–Miyaura cross-coupling reaction of **80** with a 3-thienylboronic acid ester. The light irradiation from excitation of **81** at λ_ex_ = 313 nm generates new absorption bands at λ_max_ = 495 nm and at around λ_max_ = 700 nm owing to the formation of the ring-closed system **82**.

**Scheme 27:**
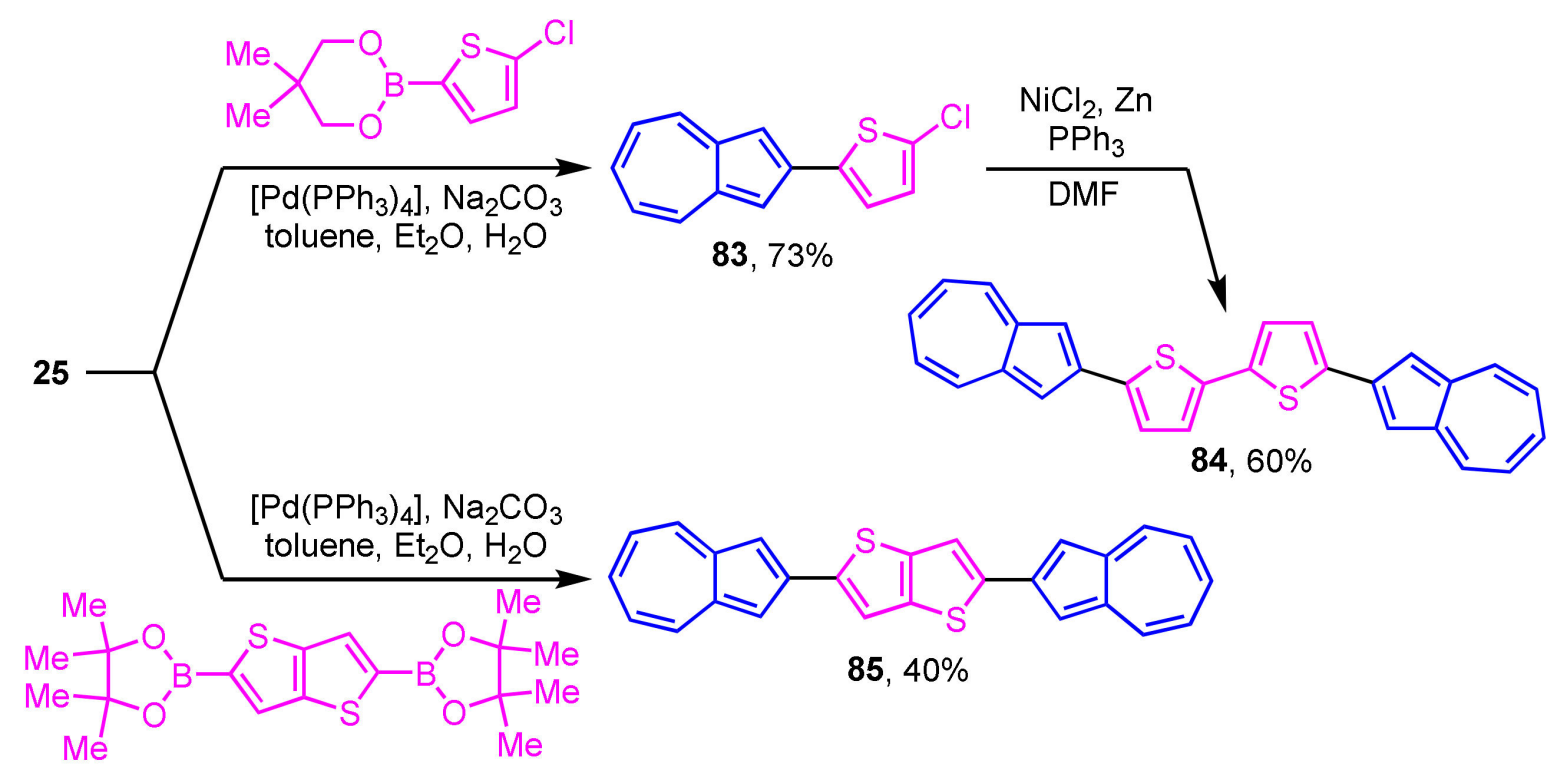
Synthesis of 2,2′-bithiophene **84** and thieno[3,2-*b*]thiophene **85** substituted by 2-azulenyl groups.

Some azulene derivatives are expected to be components of organic semiconductors due to their unique electronic properties. The synthesis of 2-arylazulene derivatives connected by 2,2′-bithiophene **84** and thieno[3,2-*b*]thiophene **85** by Suzuki–Miyaura cross-coupling reaction was reported by Katagiri et al. (Scheme 27) [45]. The single-crystal X-ray analysis revealed the herringbone packing in the crystal structure of **84** and **85**. Compounds **84** and **85** are aligned almost perpendicular to the substrate in the film form and show the characteristics of an OFET having a hole mobility.

**Scheme 28:**
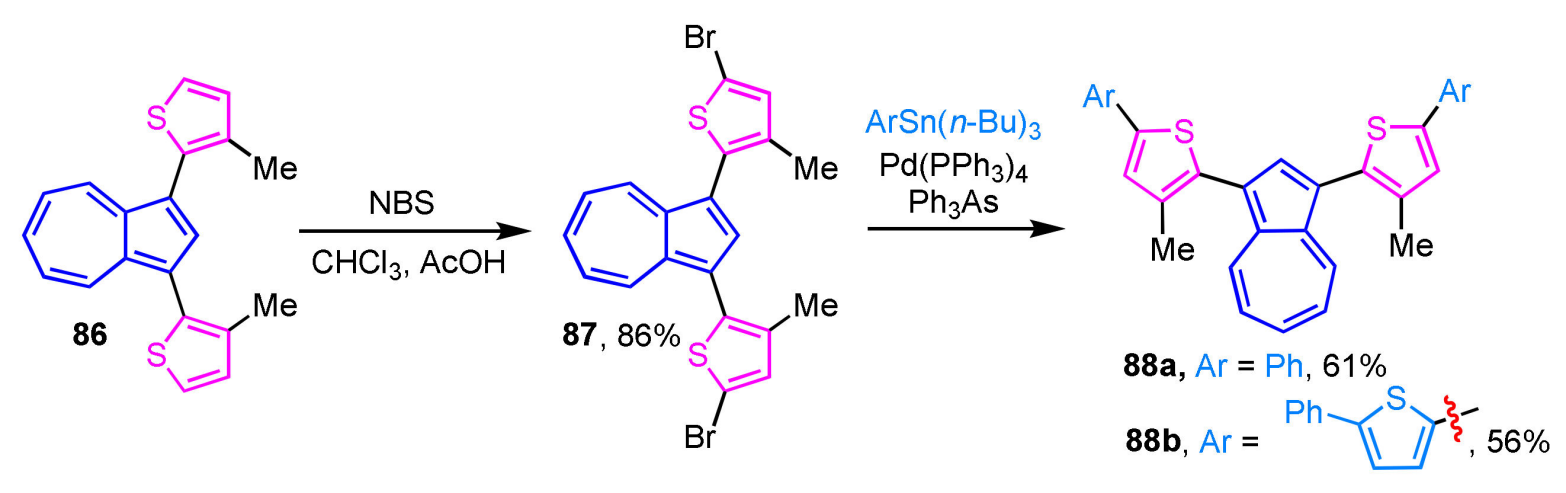
Synthesis of 1,3-di(5-aryl-2-thienyl)azulenes **88a** and **88b** by Stille cross-coupling reaction.

The synthesis of 1,3-di(2-thienyl)azulene derivatives **88a** and **88b** with an extended π-conjugated system has been reported. Bromination of **86** at the 5-position of the two 2-thienyl moieties can be enabled with NBS to give **87**, which reacts with tri-*n*-butylaryltin reagents in the presence of tetrakis(triphenylphosphine)palladium(0) and triphenylarsine ligand to afford **88a** and **88b** in 61% and 56% yields, respectively (Scheme 28) [46]. The UV/Vis spectrum of **88a** and **88b** in CHCl_3_ shows the characteristic absorption band of azulene derivatives, but protonation of **88a** and **88b** with trifluoroacetic acid shows noticeable changes of their absorption spectra both in the visible and near-IR regions.

**Scheme 29:**
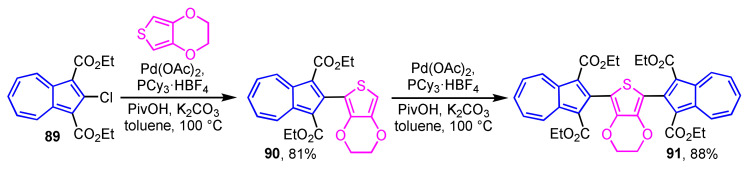
Synthesis of 2-(2-thienyl)azulene **90** and 2,5-di(2-azulenyl)thiophene **91** by palladium-catalyzed C–H bond activation reaction.

Direct preparation of 2-thienylazulene derivatives by employing the C–H bond activation reaction has also been reported (Scheme 29) [47]. The reaction of 2-chloroazulene derivative **89** with 3,4-ethylenedioxythiophene (EDOT) in the presence of Pd(OAc)_2_, PCy_3_·HBF_4_, pivalic acid (PivOH), and K_2_CO_3_ to produce 2-thienylazulene derivative **90** in 81% yield, which reacts further with **89** to afford symmetric 2,5-di(2-azulenyl)thiophene derivative **91** in 88% yield.

**Scheme 30:**
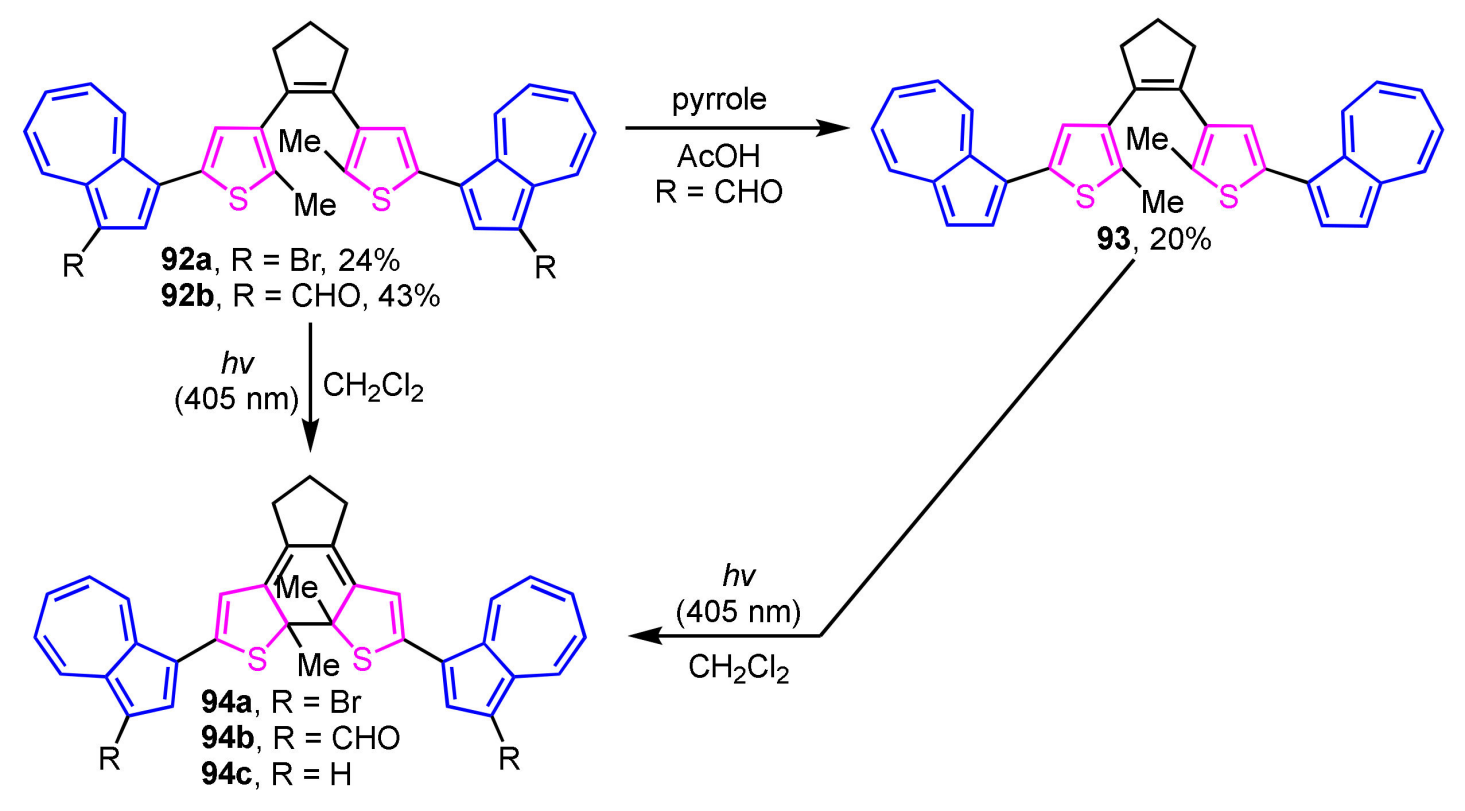
Photoinduced ring-closure reaction of dithienylethenes **92a**, **92b**, and **93**.

The synthesis of photochromic 1-(2-thienyl)azulene-based diarylethene derivatives **92a** and **92b** has been achieved by Nica and co-workers utilizing Suzuki–Miyaura cross-coupling reaction (Scheme 30) [48]. Compound **93** was also synthesized via deformylation by the treatment with pyrrole in acetic acid using **92b** as a starting material. Photocyclization of compounds **92a**, **92b**, and **93** in CH_2_Cl_2_ takes place by irradiating the light at λ_ex_ = 405 nm with the color change of the solution from greenish-yellow to blue-purple due to form ring-closing derivatives **94a**–**94c**.

**Scheme 31:**
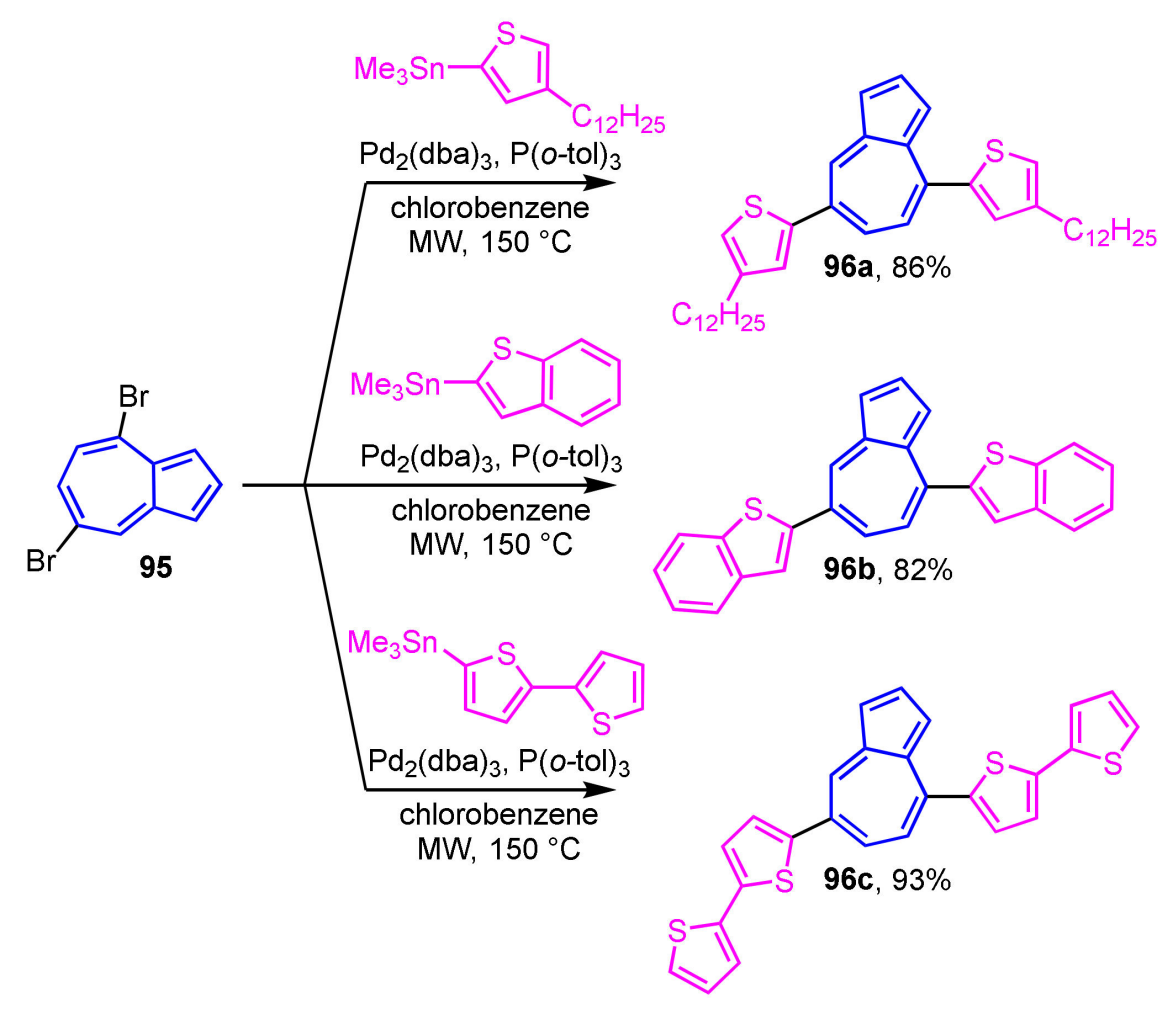
Synthesis of 4,7-di(2-thienyl)azulenes **96a**–**96c** by Stille cross-coupling reaction under the microwave irradiation conditions.

Hawker et al. reported the Stille cross-coupling reaction of 4,7-dibromoazulene **95** with 2-thienyltin reagents under the microwave irradiation conditions to give 4,7-di(2-thienyl)azulene derivatives **96a**–**96c** (Scheme 31) [49,50]. In the UV/Vis spectrum of these 4,7-di(2-thienyl)azulene derivatives, a weak absorption band arising from the azulene skeleton is observed in CH_2_Cl_2_, while a strong absorption band is appeared at around λ_max_ = 500 nm in CF_3_CO_2_H. Compound **96a** shows a strong fluorescence at λ_FL_ = 573 nm in CF_3_CO_2_H, which is not observed in CH_2_Cl_2_.

**Scheme 32:**
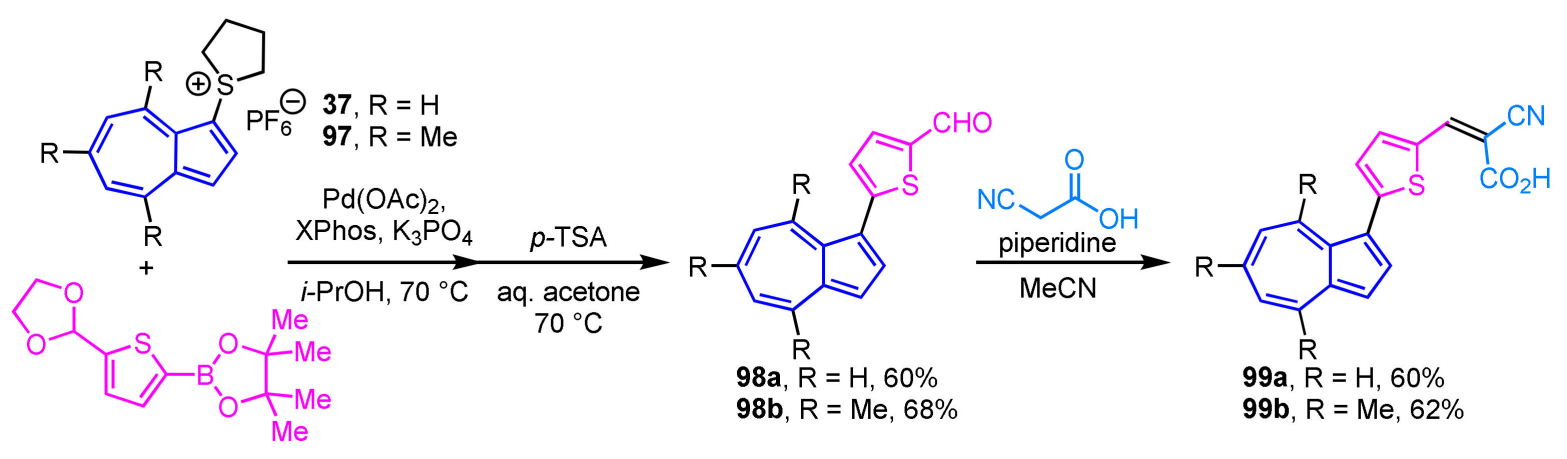
Synthesis of azulene-containing dyes **99a** and **99b** via Knoevenagel condensation of 1-(5-formyl-2-thienyl)azulenes **98a** and **99b**.

Lewis et al. reported the preparation of azulene-containing dyes **99a** and **99b** and evaluated their performance as dye-sensitized solar cells (DSSCs) [51]. The synthesis of these azulene dyes **99a** and **99b** was accomplished in four to five steps, including the cross-coupling reaction of 1-azulenylsulfonium salts **37** and **97** with the corresponding thiophene-2-boronic acid following Knoevenagel condensation (Scheme 32). These azulene dyes **99a** and **99b** are characterized as sensitizers in DSSCs concerning their electrochemical nature and crystal structure.

**Scheme 33:**
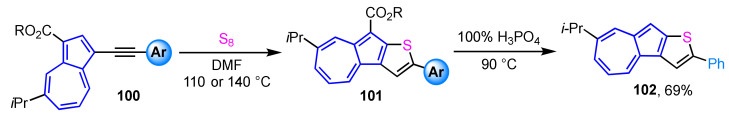
Synthesis of azuleno[2,1-*b*]thiophenes **101** by the reaction of azulenyl alkynes with elemental sulfur and preparation of **102** by following decarboxylation with 100% H_3_PO_4_.

A method for the preparation of azuleno[2,1-*b*]thiophene derivatives **101** with aryl substituent at the thiophene moiety was reported [52]. In this synthetic procedure, azuleno[2,1-*b*]thiophene derivatives **101** can be obtained in one step by the reaction of readily available azulenyl alkynes **100** with elemental sulfur (Scheme 33). The azuleno[2,1-*b*]thiophene **101** with a phenyl substituent is converted to **102** by decarboxylation with 100% H_3_PO_4_. Both the UV/Vis measurements and DFT calculations show that the optical properties of azuleno[2,1-*b*]thiophenes are significantly affected by the electronic nature of the aryl substituents on the thiophene moiety. The decarboxylated derivative **102** in CH_2_Cl_2_ displays a similar absorption band in the visible region to that of the corresponding ester derivative, while the addition of CF_3_CO_2_H to the solution shows a pronounced color change, so-called halochromism, due to the protonation of the 5-membered ring of azuleno[2,1-*b*]thiophene to produce a tropylium ion substructure.

### 3.2. Azulene Derivatives with N-Containing 5- and 6-Membered Heterocycles

The compounds with pyrrole and indole skeletons can be found in many natural products, bioactive substances, and pharmaceuticals. Several reports on the synthesis of pyrrole and indole derivatives connected to the azulene ring have been published.

**Scheme 34:**
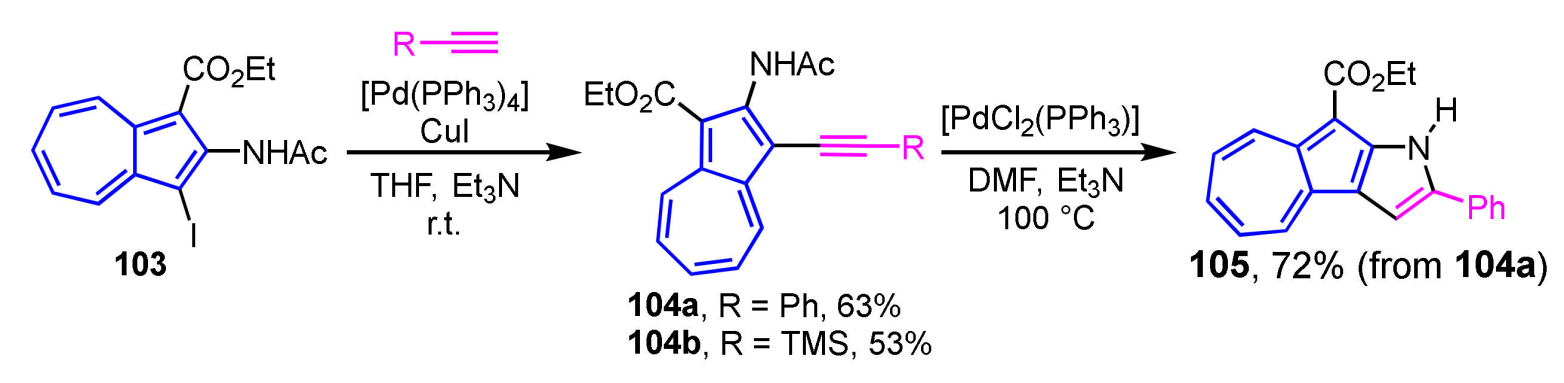
Synthesis of azuleno[2,1-*b*]pyrrole **105** by palladium-catalyzed cyclization of **104a**.

Abe et al. have achieved two-step synthesis of pyrrole-fused azulene derivatives using 1-iodo-2-acetoaminoazulene derivatives as a starting material (Scheme 34) [53]. Sonogashira cross-coupling reaction of iodo derivative **103** with the corresponding alkynes yielded 2-acetylamino-1-ethynylethynyazulenes **104a** and **104b** in good yields. Cyclization of **104a** in the presence of PdCl_2_(PPh_3_)_2_ and Et_3_N as a base in DMF gives azuleno[2,1-*b*]pyrrole **105** in 72% yield accompanied by the elimination of the acetyl group on the nitrogen atom.

**Scheme 35:**
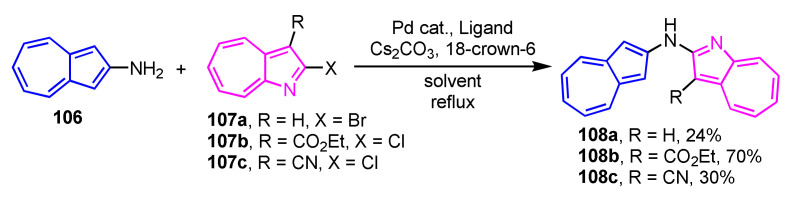
Synthesis of (2-azulenyl)(1-aza-2-azulenyl)amines **108a**–**108c** by Hartwig–Buchwald reaction.

Tsukada and co-workers have demonstrated the synthesis of (2-azulenyl)(1-aza-2-azulenyl)amines **108a**–**108c** containing azulene and 1-azaazulene linkage via a nitrogen atom (Scheme 35) [54]. The Hartwig–Buchwald reaction of 2-aminoazulene **106** with 2-halo-1-azaazulenes **107a**–**107c** was employed for the synthesis of **108a**–**108c**. As a result of the investigation of several conditions, the catalytic system using PdCl_2_(dppf)/BINAP in THF provides the best results.

**Scheme 36:**
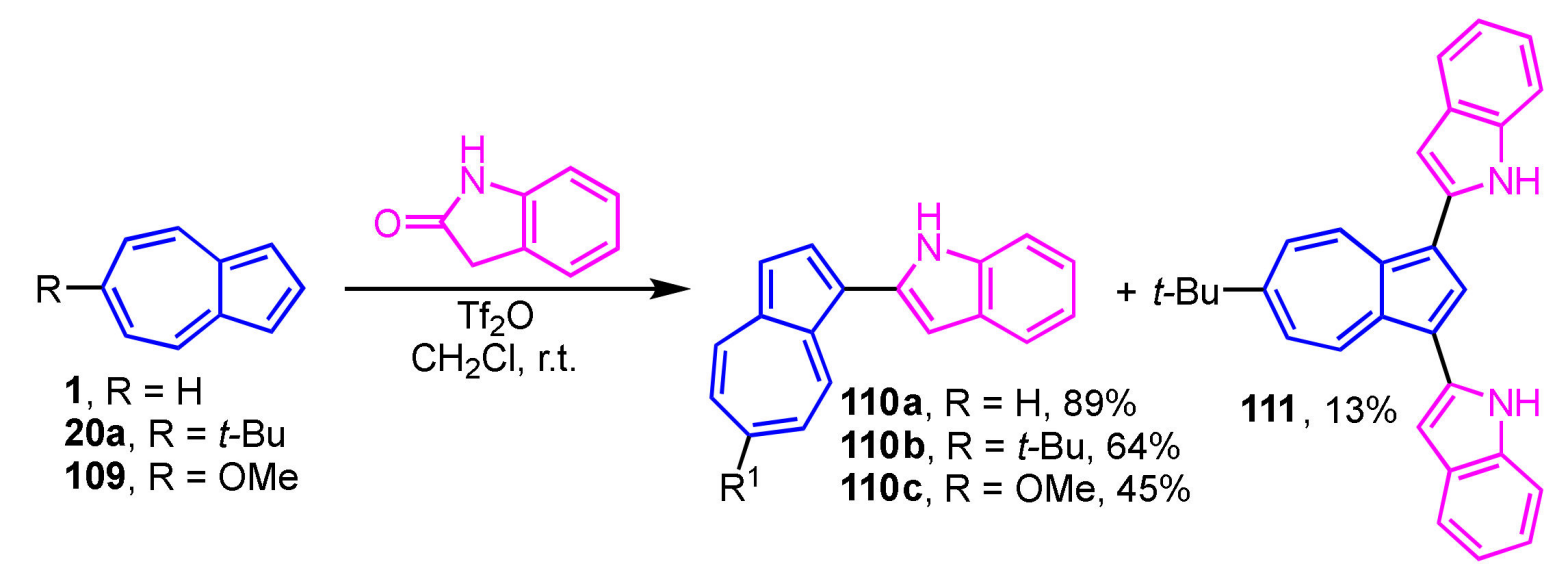
Synthesis of 1-(indol-2-yl)azulenes **110a**–**110c** and **111** by Vilsmeier–Haack-type reaction.

Vilsmeier–Haack-type reaction of azulene derivatives with 2-indolinone was conducted in the presence of Tf_2_O affords 1-(indol-2-yl)azulenes (Scheme 36) [55]. In this research, the substituent at the 6-position of the azulene ring was found to affect the reactivity, significantly. The reaction of **1** and **109** produces mono-substituted products **110a** and **110c**, whereas **20a** gives **110b** (64% yield) as the main product along with the di-substituted product **111** (13% yield).

**Scheme 37:**
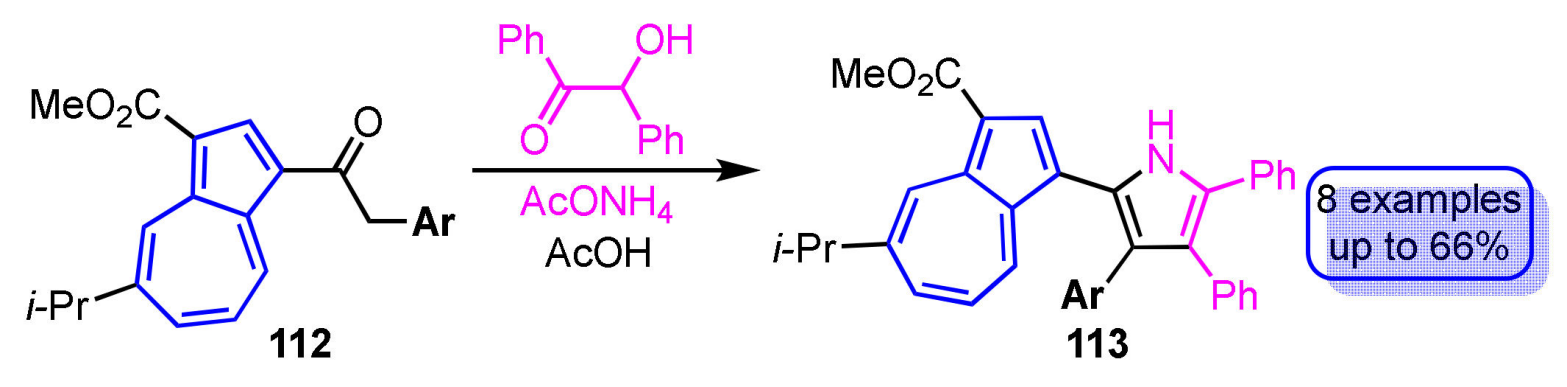
Synthesis of tetraarylpyrroles **113** by cyclization of benzoin with 1-azulenyl ketones **112**.

The synthesis of tetraarylpyrroles **113** with a 1-azulenyl substituent has been accomplished by cyclization of benzoin with 1-azulenyl ketones **112** with various aryl groups at the α-position in the presence of ammonium acetate as the nitrogen source for the pyrrole ring (Scheme 37) [56]. The yield of the reaction depends largely on the electronic properties of the aryl group substituted at the α-position of the ketones **112**. The origin of the absorption bands observed in the UV/Vis spectra of the tetraarylpyrroles **113** with a 1-azulenyl substituent was clarified by time-dependent density functional theory (TD-DFT) calculations.

**Scheme 38:**
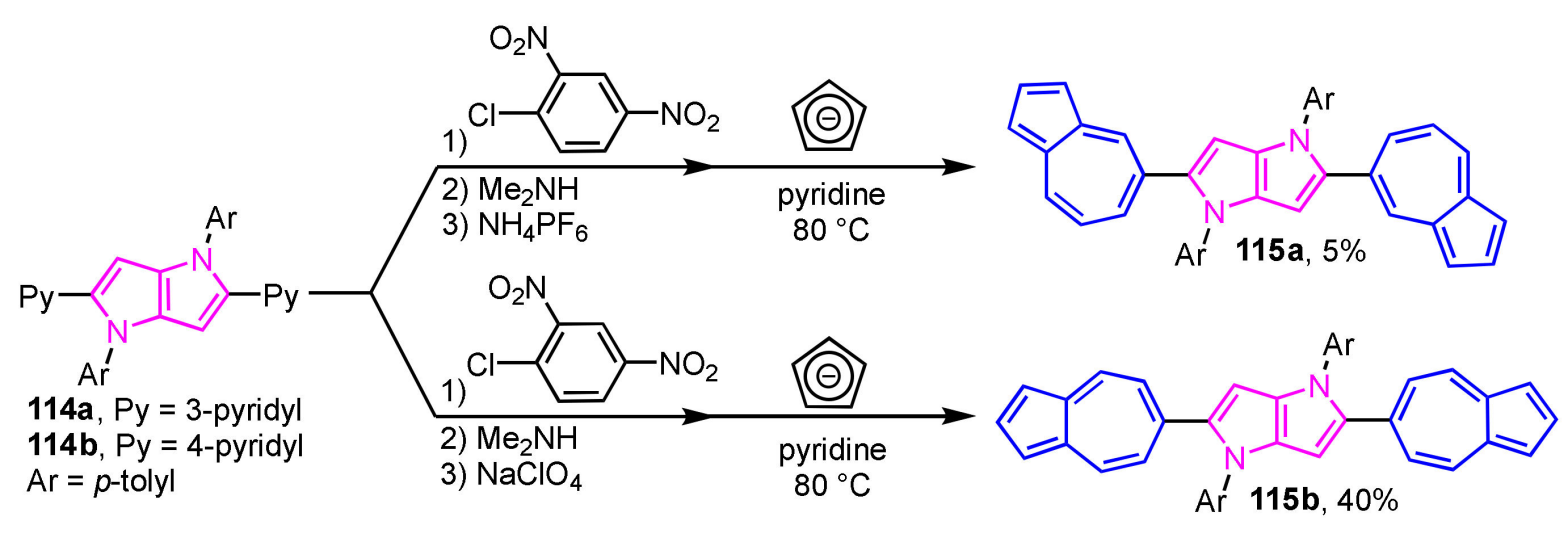
Synthesis of azulene-substituted pyrrolo[3,2-*b*]pyrroles **115a** and **115b** via Ziegler–Hafner method.

A synthetic route to pyrrolo[3,2-*b*]pyrroles **115a** and **115b** with two 5-azulenyl or 6-azulenyl groups have been developed by Gryko et al. (Scheme 38) [57]. In this method, pyridine-substituted pyrrolo[3,2-*b*]pyrroles **114a** and **114b** are converted, in three steps, to azulene-substituted pyrrolo[3,2-*b*]pyrroles **115a** and **115b** via Ziegler–Hafner method, i.e., successive *N*-arylation, ring-opening with dimethylamine, and the reaction with cyclopentadienide ion. The optical properties of azulene-substituted pyrrolo[3,2-*b*]pyrroles **115a** and **115b** were evaluated in terms of absorption spectra and quantum chemical calculations using TD-DFT.

**Scheme 39:**
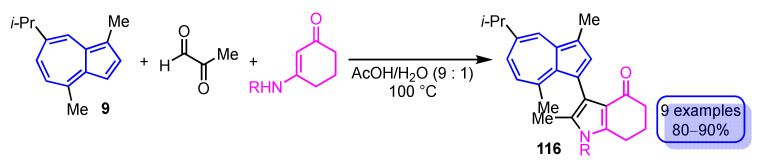
Synthesis of 3-(guaiazulen-3-yl)dihydro-1*H*-indol-4(5*H*)-ones **116** by the three-component domino reaction.

In 2019, Wang et al. reported two methods for the synthesis of pyrrole-substituted azulene derivatives via the reaction of guaiazulene (**9**) with methylglyoxal in common (Scheme 39). The first of the two procedures is the three-component domino reaction of **9**, methylglyoxal and enaminones under the acid catalyzed conditions to obtain 3-(guaiazulen-3-yl)dihydro-1*H*-indol-4(5*H*)-ones **116** in good yields (80–90%) [58].

**Scheme 40:**
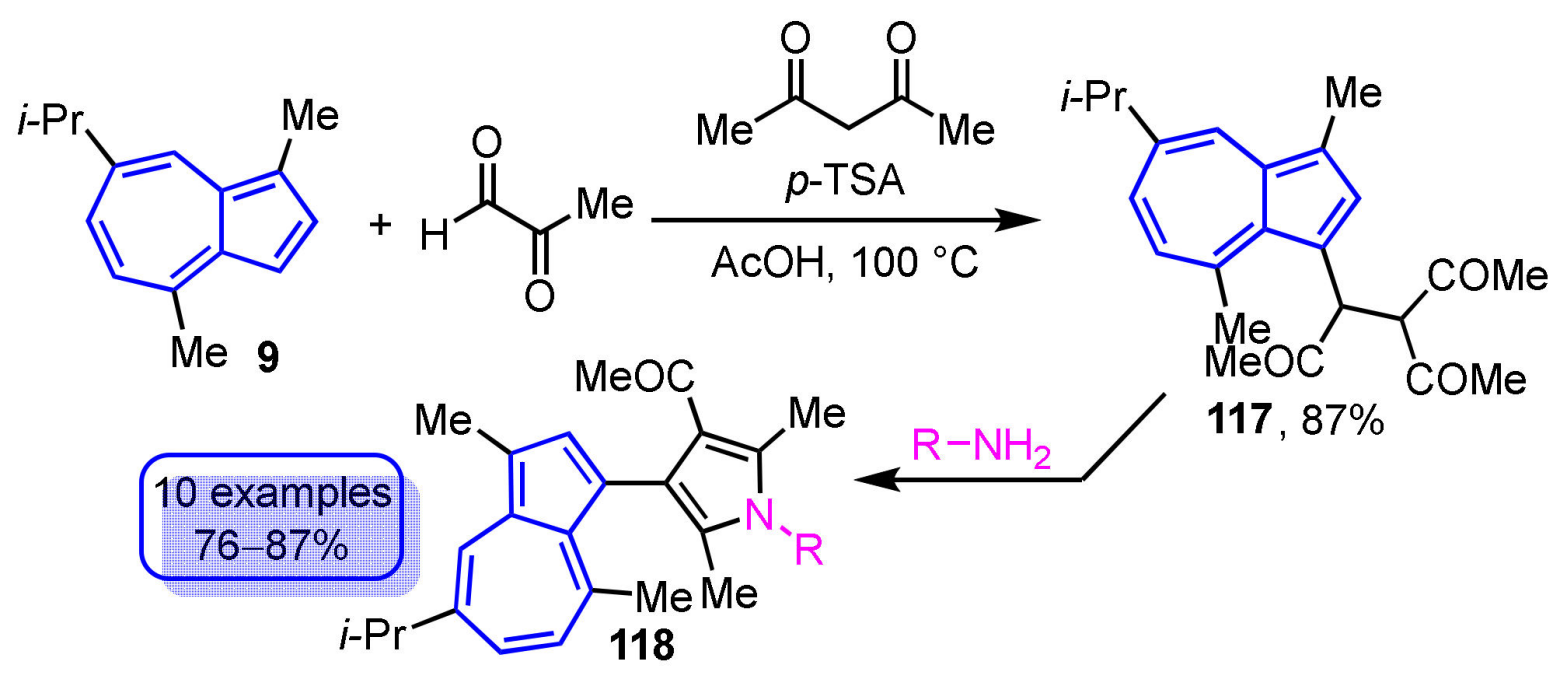
Synthesis of 3-(guaiazulen-3-yl)pyrroles **118** via Paal–Knorr reaction of 3-acetyl-4-(guaiazulen-3-yl)hexane-2,5-dione (**117**).

In another method, the synthesis of 3-(guaiazulen-3-yl)pyrroles **118** has been achieved by the Paal–Knorr reaction of 3-acetyl-4-(guaiazulen-3-yl)hexane-2,5-dione (**117**), which is obtained by the reaction of **9**, methylglyoxal, and acetylacetone in the presence of acetic acid via primary amines (Scheme 40) [59]. Both of these synthetic methods provide a facile method for the preparation of pyrrole-substituted azulene derivatives.

**Scheme 41:**
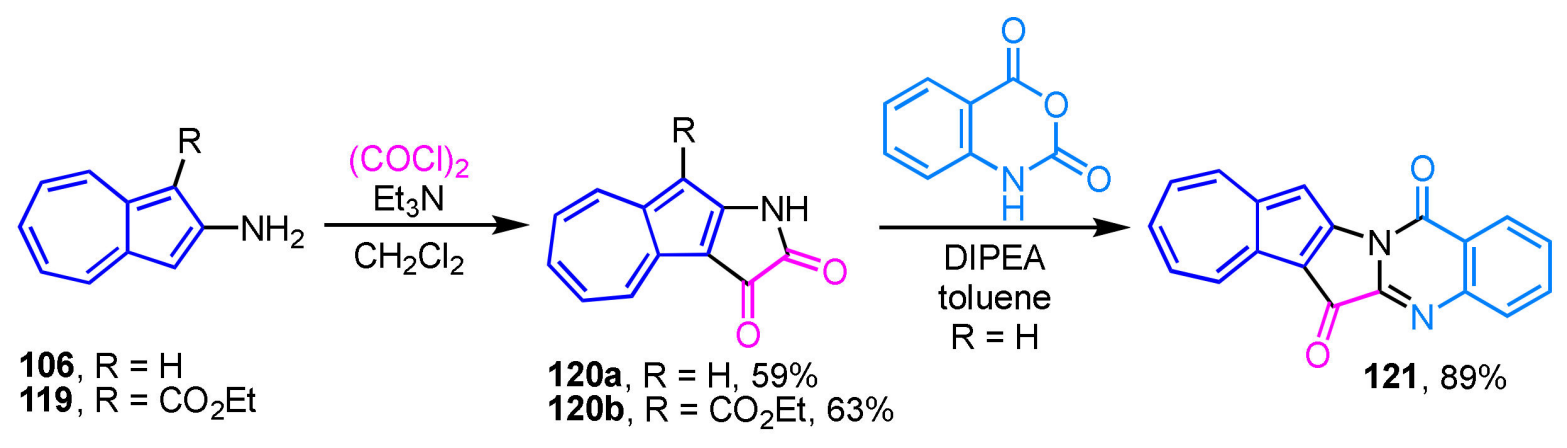
Synthesis of azuleno[1′,2′:4,5]pyrrolo[2,1-*b*]quinazoline-6,14-dione (**121**).

The synthesis of azulene-fused tryptanthrin, i.e., azuleno[1′,2′:4,5]pyrrolo[2,1-*b*]quinazoline-6,14-dione (**121**), has recently been reported (Scheme 41) [60]. The synthesis of **121** is accomplished by the condensation reaction of azuleno[2,1-*b*]pyrrole-2,3-dione (**120a**), which is prepared by the reaction of 2-aminoazulene (**106**) with oxalyl chloride, with isatoic anhydride in the presence of sodium hydride or diisopropyl ethylamine (DIPEA) as a base. The ester derivative **120b** provides no desired product due to the decomposition under the similar reaction conditions. Halogenation reaction of **121** using NBS and NIS results in high yields of 12-halo derivatives. However, even though the reaction of NBS smoothly proceeds at room temperature, the reaction with NIS requires a relatively high reaction temperature (refluxing in 1,2-dichloroethane). The redox properties of the new azulene-fused tryptanthrins are evaluated by spectroscopic and voltammetric analyses.

### 3.3. Azulene Derivatives with O-Containing 5-Membered Heterocycles

Although there are not many reports for the synthesis of azulene derivatives containing furans and their derivatives in the molecule, various approaches such as cross-coupling and intramolecular cyclization have been applied to the preparation of such compounds.

**Scheme 42:**
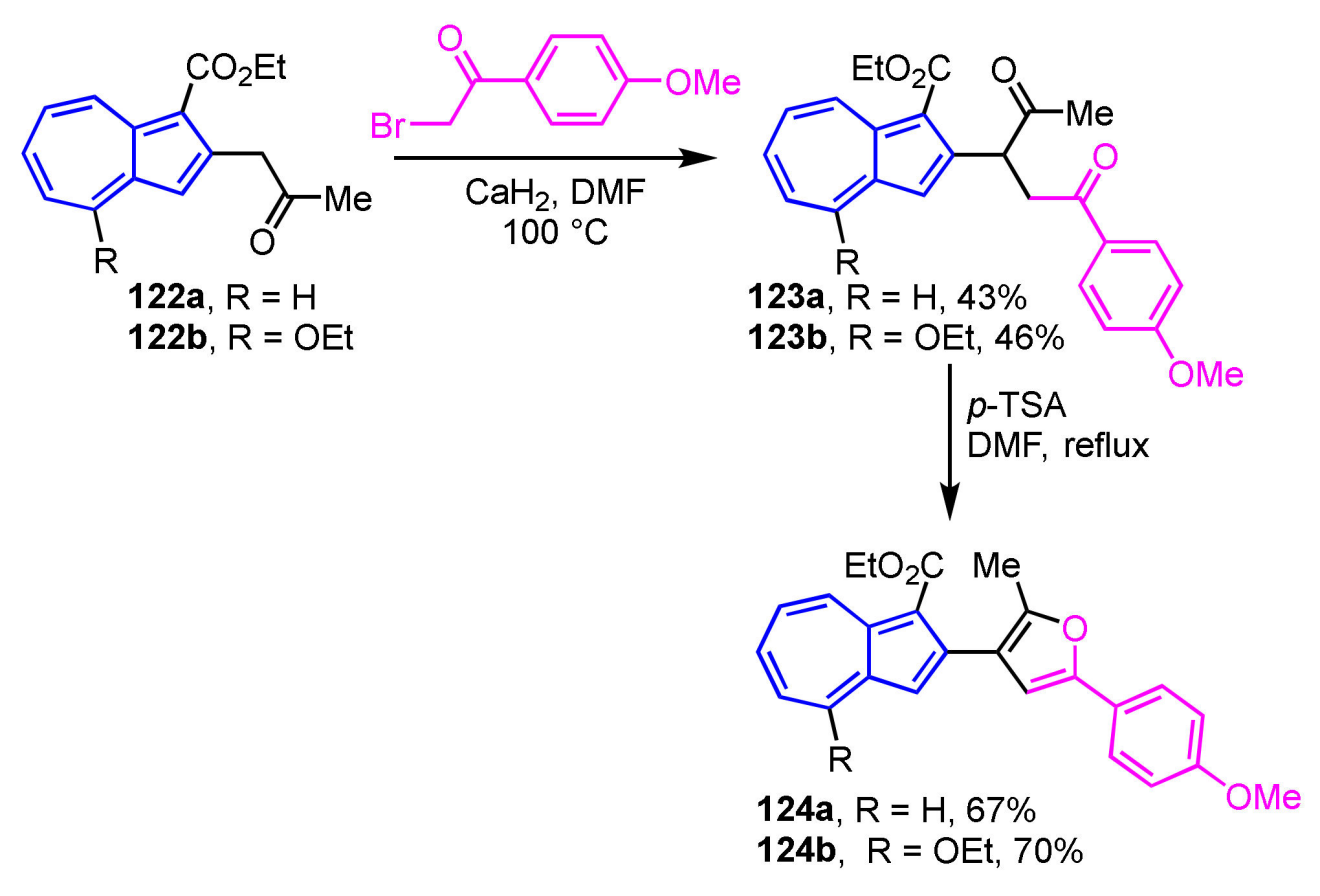
Synthesis of 2-(3′-furyl)azulenes **124a** and **124b** via Paal–Knorr reaction.

Wu, Ku, and their co-workers have reported the two-step synthesis of 2-(3-furyl)azulene derivatives **124a** and **124b [61]**. The diketone derivatives **123a** and **123b** are prepared by the reaction of **122a** and **122b** with 4′-methoxyphenacyl bromide in the presence of CaH_2_ in DMF. Compounds **124a** and **124b** can be obtained by Paal–Knorr reaction, i.e., treatment with *p*-TSA in DMF, in 67% and 70% yields, respectively (Scheme 42).

**Scheme 43:**
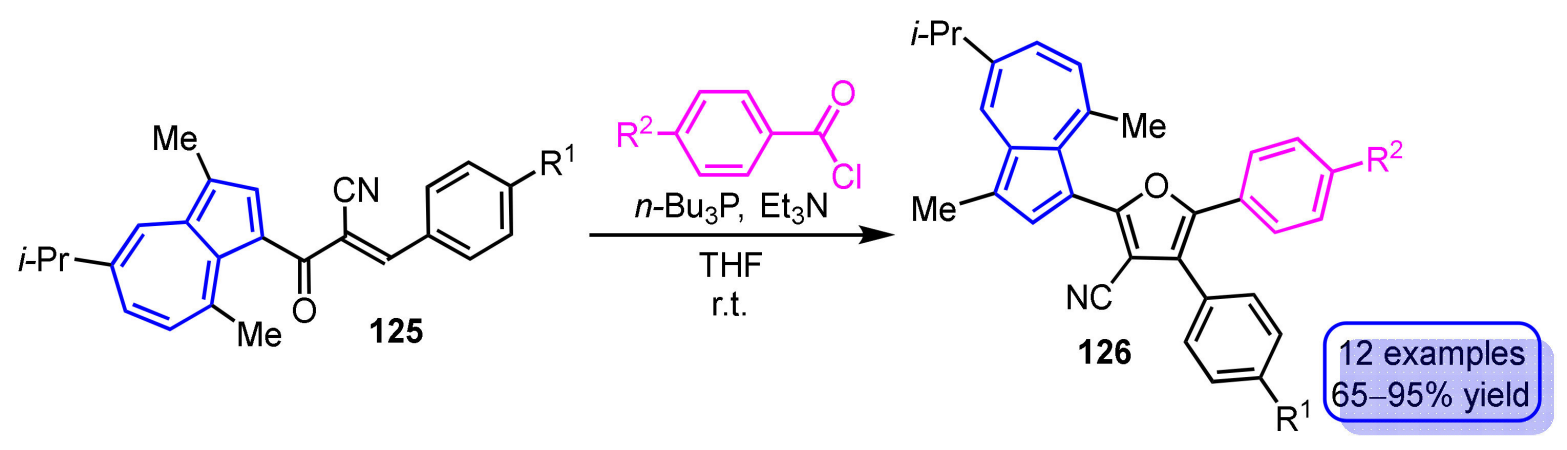
Phosphine-mediated synthesis of 3-(2-furyl)guaiazulene derivatives **126**.

The synthesis of 3-(2-furyl)guaiazulene derivatives **126** by phosphine-mediated intermolecular cyclization of 3-(3-phenyl-2-cyanopropenoyl)guaiazulenes **125** with benzoyl chlorides is demonstrated by Wang and Xu et al. (Scheme 43) [62]. This reaction provides 3-(2-furyl)guaiazulene derivatives **126** in excellent yields when the substituent R^2^ on the benzoyl chloride is an electron-donating group, while the yield of the products is slightly lower when the substituent R^2^ has an electron-withdrawing nature.

**Scheme 44:**
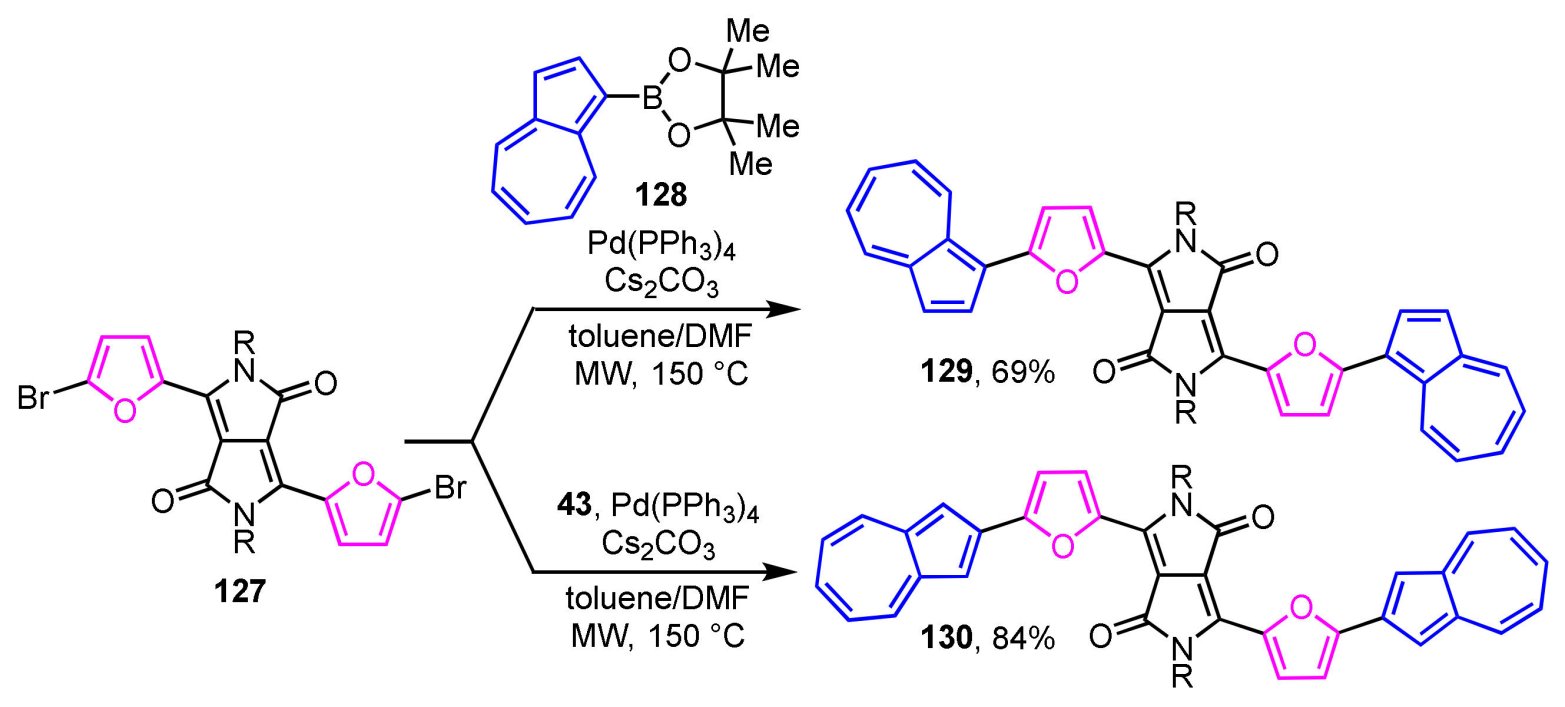
Synthesis of 1- and 2-(2-furyl)azulenes **129** and **130** with a diketopyrrolopyrrole by microwave-assisted Suzuki–Miyaura cross-coupling reaction.

The synthesis and optical properties of π-extended derivatives **129** and **130** with two 2-(2-furyl)azulene units connected to diketopyrrolopyrrole have been reported by Hawker et al. (Scheme 44) [63]. The two regioisomers **129** and **130** are obtained in 69% and 84% yields, respectively, by the Suzuki–Miyaura cross-coupling reaction of 1- and 2-azulenylboronates **128** and **43** with 3,6-bis(5-bromo-2-furyl)pyrrolo[3,4-*c*]pyrrole-1,4-dione (**127**) in a mixed solvent of toluene and DMF by microwave irradiation.

**Scheme 45:**
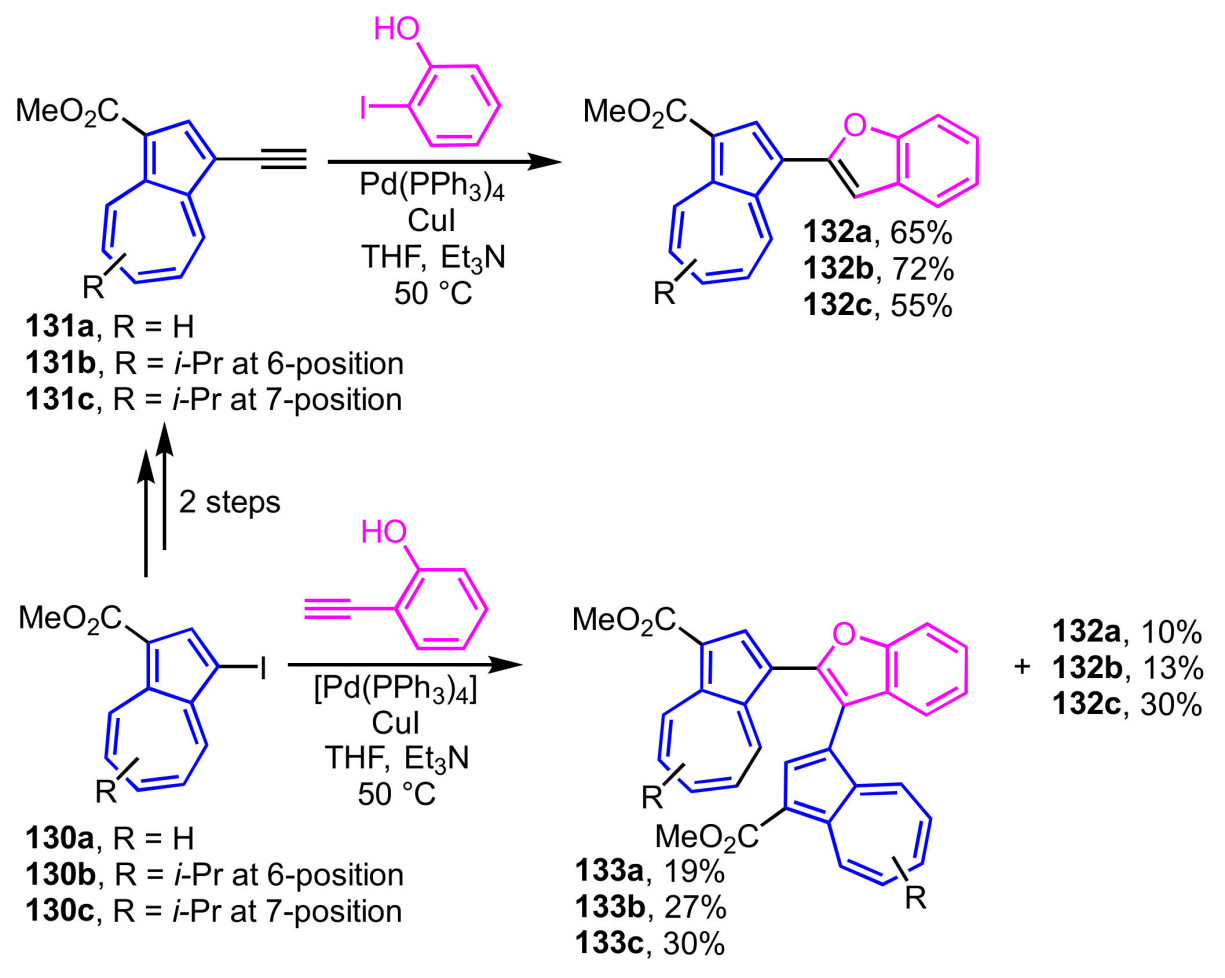
Synthesis of 2-(1-azulenyl)benzofurans **132a**–**132c** and 2,3-di(1-azulenyl)benzofurans **133a**–**133c** under Sonogashira–Hagihara reaction conditions.

2-(1-Azulenyl)benzofurans **132a**–**132c** are obtained in one pot under the Sonogashira–Hagihara reaction conditions by the reaction of 1-ethynylazulene derivatives **131a**–**131c**, which are prepared from 1-iodoazulenes **130a**–**130c** as starting materials, with 2-iodophenol (Scheme 45) [35]. By contrast, the reaction of **130a**–**130c** with 2-ethynylphenol under the similar conditions affords 2,3-di(1-azulenyl)benzofurans **133a**–**133c** along with **132a**–**132c** as a byproduct. The structures of these compounds were clarified by single-crystal X-ray structure analysis.

**Scheme 46:**
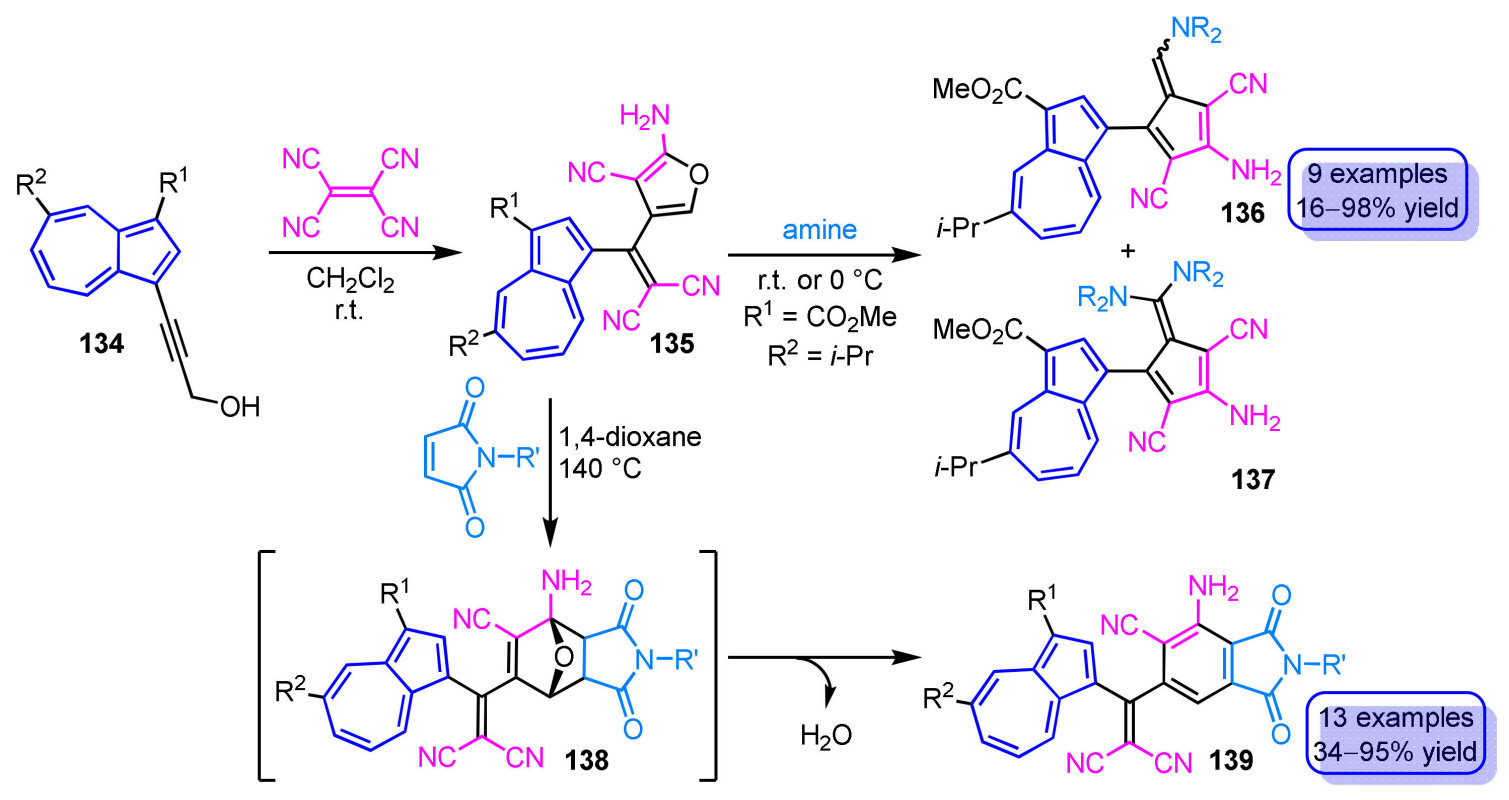
Synthesis of 2-aminofurans **135** by the reaction of 3-(1-azulenyl)propargyl alcohols **134** with tetracyanoethylene and conversion of **135** to 6-aminopentafulvenes **136**, **137** and phthalimides **139**.

The reaction of 3-(1-azulenyl)propargyl alcohols **134** with tetracyanoethylene produces 2-aminofuran derivatives **135** cross-conjugated by azulene-ring in excellent yields (Scheme 46) [64]. The formation of the 2-amino furan ring is believed to derive from the [2 + 2] cycloaddition–retroelectrocyclization of alkynes with tetracyanoethylene and subsequent nucleophilic addition of the hydroxy group to one of the cyano groups. The 2-aminofuran **135** can be converted into 6-aminopentafulvenes **136** and 6,6-diaminopentafulvenes **137** in moderate to excellent yields by treatment with an excess of primary and secondary amines. Diels–Alder reaction of **135** with maleimides affords phthalimide derivatives **139** cross-conjugated with an azulene ring in one pot and without the isolation of presumed [4 + 2] cycloadducts **138** [65]. Even though **139** does not exhibit luminescence in CH_2_Cl_2_, remarkable fluorescence is observed in a mixed solvent of CH_2_Cl_2_ and CF_3_CO_2_H.

### 3.4. Azulene Derivatives with Other Ring Heterocycles

In addition to the aforementioned, preparation of various azulene derivatives with a variety of heterocycles have been reported. An overview of the derivatives with other 5-membered ring heterocycles is given below.

**Scheme 47:**
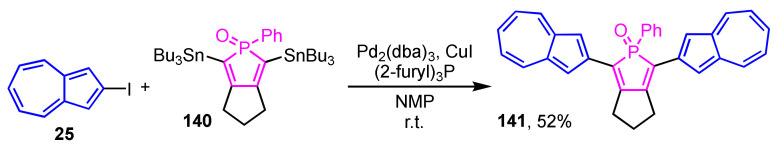
Synthesis of 2,5-di(2-azulenyl)phosphole **141** by Stille cross-coupling reaction.

Matano et al. reported the synthesis and optical properties of phosphole derivative **141** substituted with two 2-azulenyl groups (Scheme 47) [66]. The synthesis is performed under Stille cross-coupling conditions by the reaction of 2,5-bis(tributylstannyl)phosphole **140** and 2-iodoazulene (**25**) using Pd_2_(dba)_3_, (2-furyl)_3_P, and CuI as catalysts, giving the target compound **141** in 52% yield. Compound **141** in CH_2_Cl_2_ shows an absorption wavelength maximum at λ_max_ = 481 nm in UV/Vis spectrum, while no emission is displayed from **141** in the same way as the usual azulene derivatives.

**Scheme 48:**
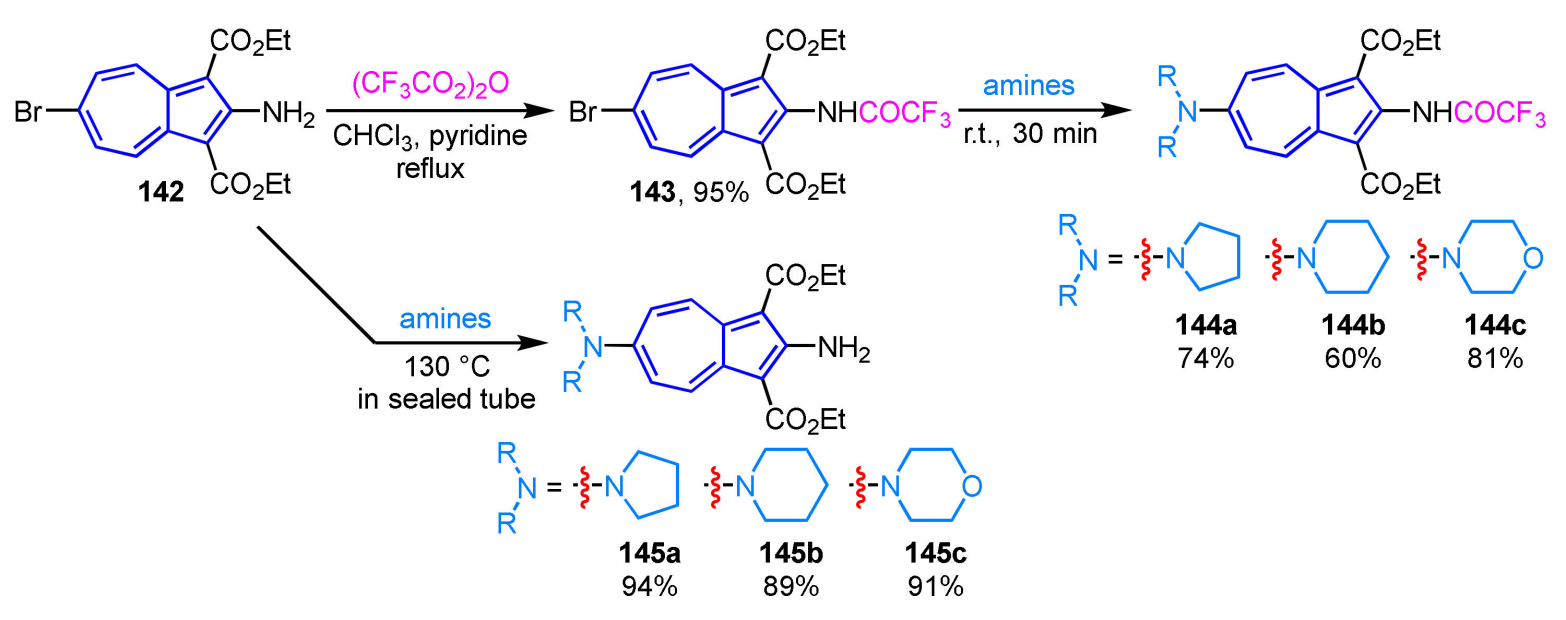
Synthesis of 2,6-diaminoazulenes **145a**–**145c** by S_N_Ar reaction with amines.

Although preparation of 2,6-diaminoazulenes has been known to be difficult, an efficient method for their synthesis by aromatic nucleophilic substitution reactions (S_N_Ar) was reported in 2015 (Scheme 48) [67]. The S_N_Ar reaction of 2-amino-6-bromoazulene derivative **142** with cyclic amines such as pyrrolidine, piperidine, and morpholine at 130 °C in a sealed tube affords the corresponding 2,6-diaminoazulene derivatives **145a**–**145c** in excellent yields. Alternatively, the amination reaction at lower temperature is achieved by the treatment of trifluoroacetamide derivative **143**, which is obtained by the reaction of **142** with trifluoroacetic anhydride, with amines at room temperature to give the corresponding 2,6-diaminoazulenes **144a**–**144c**. The trifluoroacetamide substituent of **145a**–**145c** is transformed into an amino group by deprotection with K_2_CO_3_ in EtOH to form **145a**–**145c**.

**Scheme 49:**
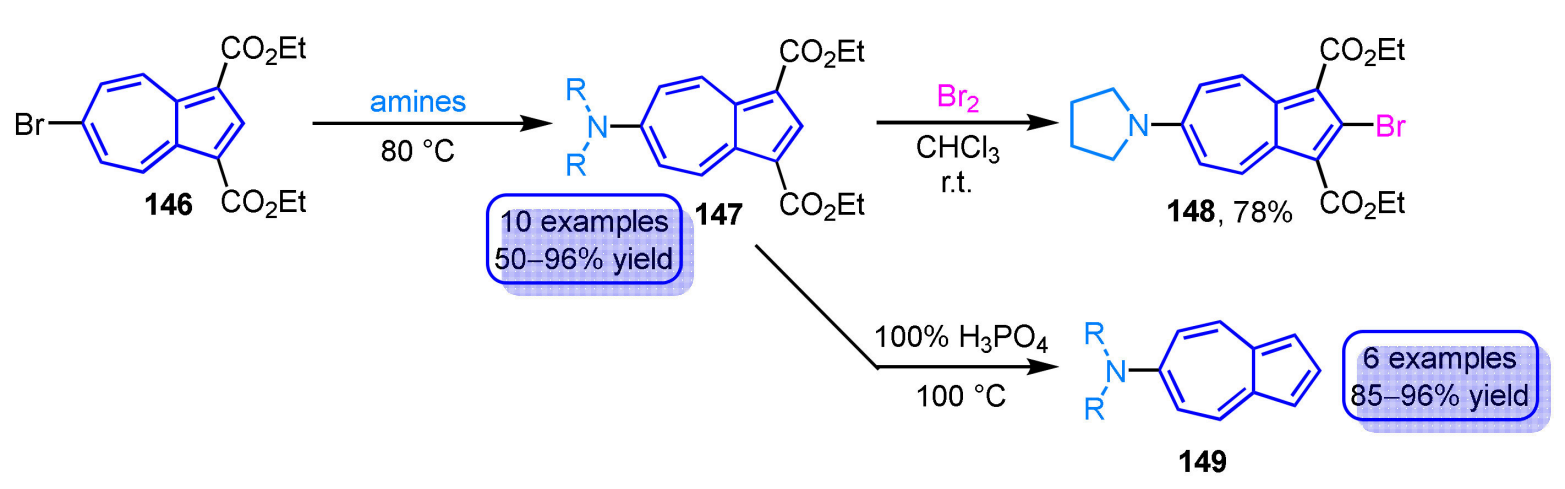
Synthesis and reactivity of 6-aminoazulene derivatives **147**.

Similar to the aforementioned reaction, the reaction of 6-bromoazulene **146** with two ester groups with amines yields the corresponding 6-aminoazulene derivatives **147** in good to excellent yields (Scheme 49) [68,69]. The electrophilic substitution reaction of a derivative with a pyrrolidine substituent at the 6-position with bromine provides 2-bromo-6-pyrrolidinylazulene derivative **148**. Since the introduction of halogen functional group at the 2-position of azulene derivatives by electrophilic substitution reactions is usually difficult, this method is one of the preferable methods for the functionalization of azulenes. The two ester groups of the 6-aminoazulene derivatives **147** can be removed by decarboxylation reaction using 100% H_3_PO_4_ to afford parent 6-aminoazulenes **149** in excellent yields.

**Scheme 50:**
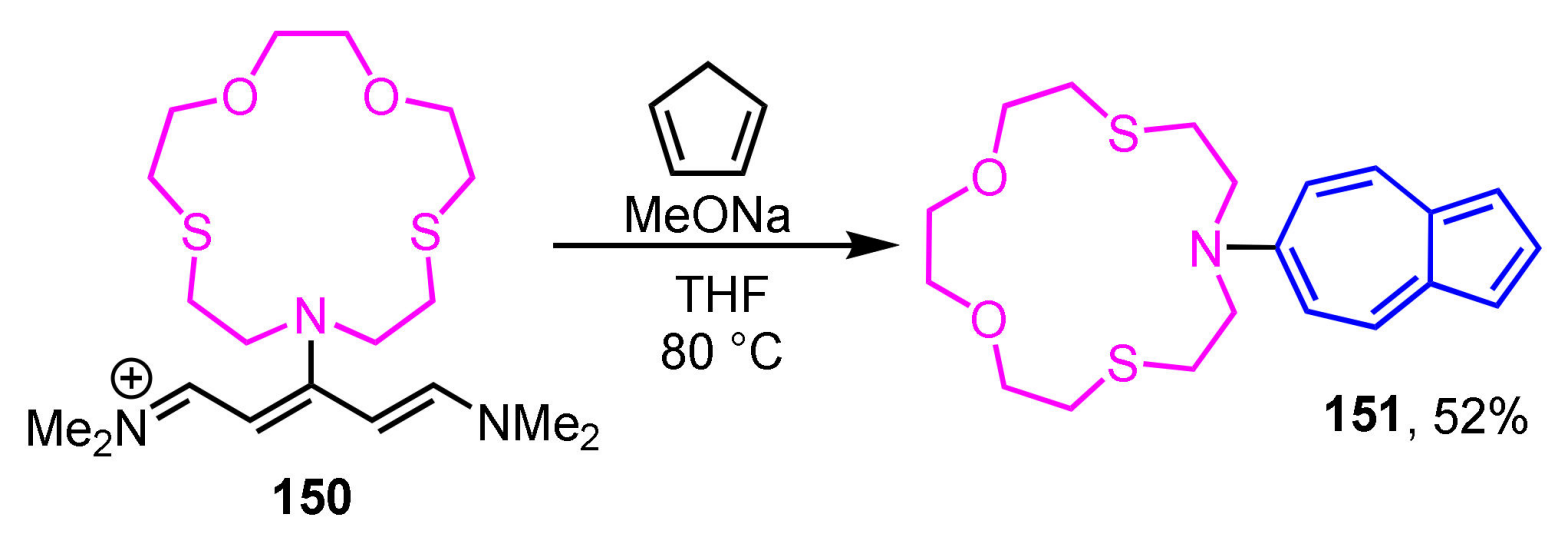
Synthesis of azathiocrown derivative **151** substituted by azulene ring at the 6-position.

In 2013, Wakabayashi et al. reported the synthesis and coordination behavior of 7-aza-4,10-dithia-1-oxacyclododecane (azathiocrown) derivative **151** substituted by azulene ring at the 6-position (Scheme 50) [70]. The synthesis of **151** is accomplished by the one-pot condensation–cyclization reaction of iminium salt **150** with cyclopentadienide ion, i.e., by Ziegler–Hafner method. Compound **151** shows a color change from red to purple when Ag^+^ ion (AgClO_4_) is added in an acetone solution. This change in coloration is believed to be caused by the interaction of Ag^+^ ions with the nitrogen atoms of azathiocrown ether, based on the ^1^H NMR experiments.

**Scheme 51:**
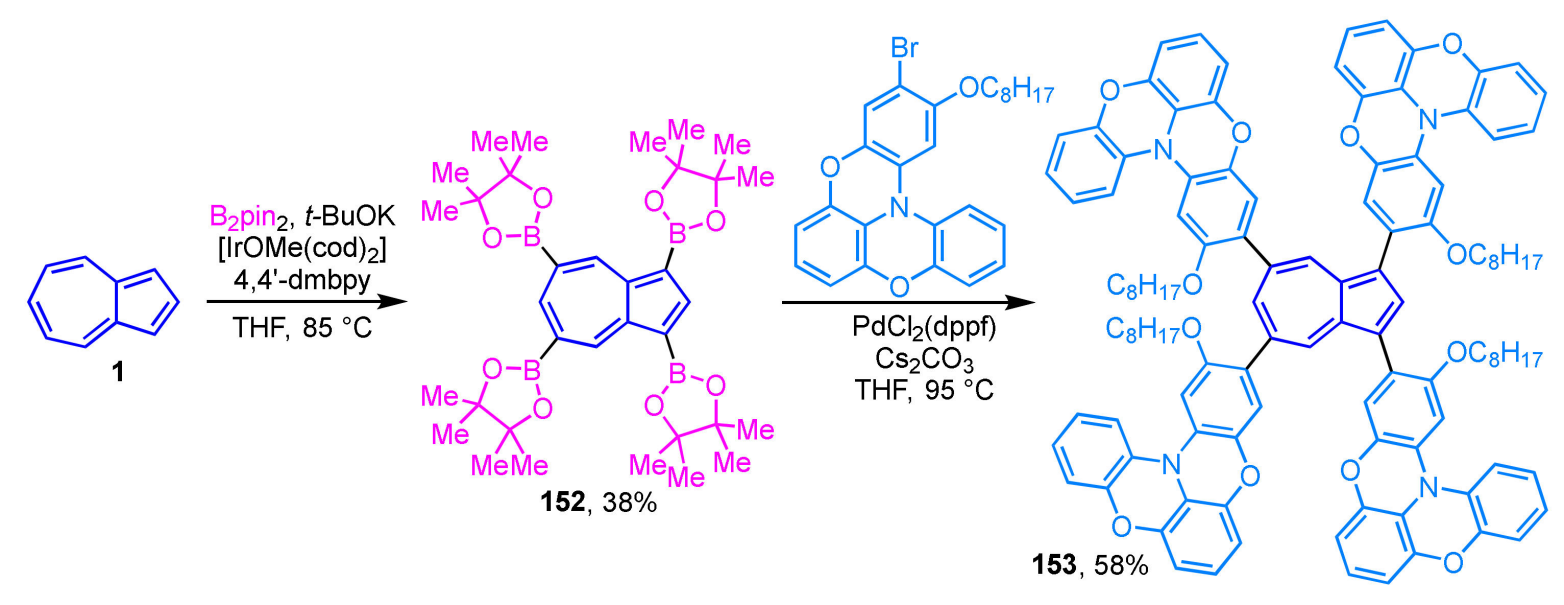
Synthesis of tetrasubstituted azulene **153** by multiple Suzuki–Miyaura cross-coupling of **152**.

Wakamiya, Scott, and their colleagues reported the synthesis and properties of extended π-electron molecules in which the oxygen-bridged triarylamines are connected to an azulene ring, for application in perovskite solar cells [71]. The precursor, 1,3,5,7-tetraborylazulene **152**, is obtained by iridium-catalyzed Hartwig–Miyaura borylation of azulene (**1**) with bis(pinacolato)diboron with 38% as isolated yield (Scheme 51) [72]. Synthesis of the target molecule, tetrasubstituted azulene **153**, is achieved by multiple Suzuki–Miyaura cross-coupling of **152** with mono-brominated oxygen-bridged triarylamine in 58% yield. The performance of tetrasubstituted azulene **153** as a hole transport material for perovskite solar cells (power conversion efficiency = 16.5%) was found to be superior to the current HTM standard, spiro-OMeTAD.

**Scheme 52:**
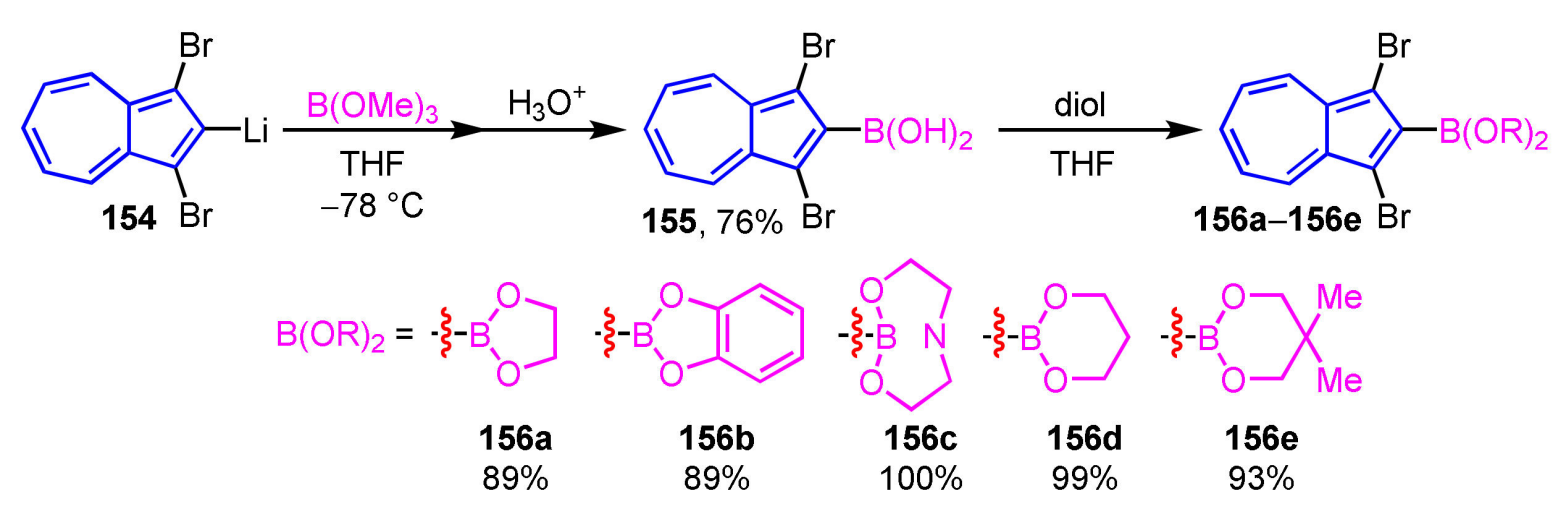
Synthesis of 2-azulenylboronic acid esters **156a**–**156e** by the reaction of 2-azulenylboronic acid **155**.

Murafuji et al. demonstrated the synthesis of azulene-substituted cyclic boronic acid esters **156a**–**156e** by the reaction of 2-azulenylboronic acid **155** with several diols (Scheme 52) [73]. The reaction of 2-azulenyl lithium **154** with trimethyl borate yields a boronic acid ester, which is then hydrolyzed by acid to give 2-azulenylboronic acid **155**. Cyclic esterification of 2-azulenylboronic acid **155** with several diols are completed within 3 h in most cases, whereas the reaction with catechol requires 42 h to complete the reaction due to the low nucleophilicity of the diol.

**Scheme 53:**
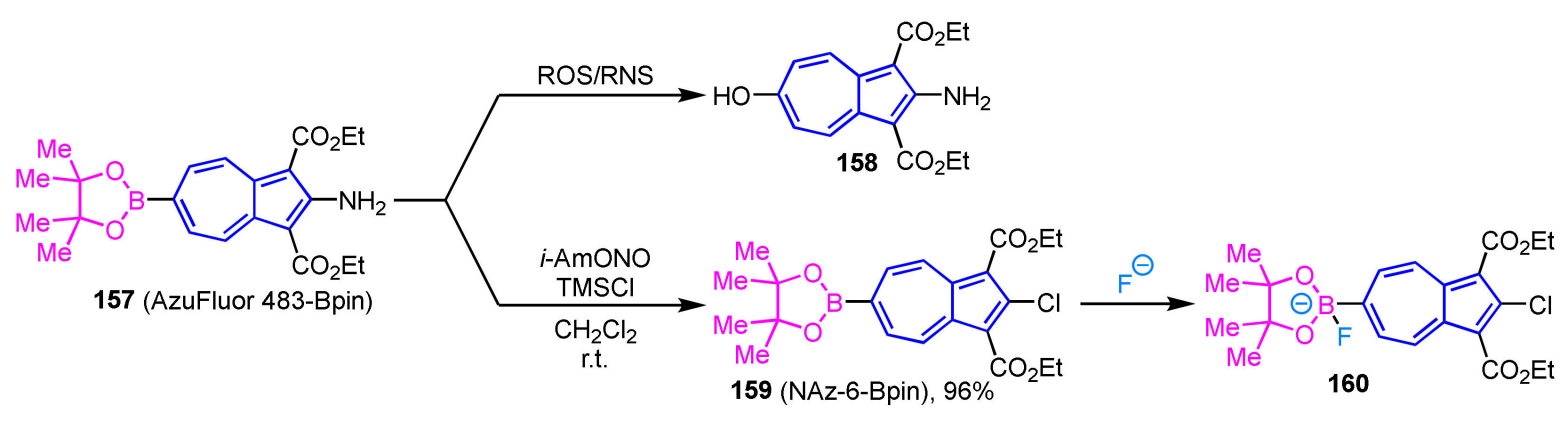
The reaction of an azulene-based fluorescent probe (AzuFluor 483-Bpin **157**).

Since the elevated concentrations of reactive oxygen species (ROS) and reactive nitrogen species (RNS) in living organisms are associated with a variety of diseases including cancer and cardiovascular disease, much attention has been paid to the development of methods to detect these active species in living cells. Lewis et al. reported AzuFluor 483-Bpin **157** that exhibits fluorescence upon reaction with ROS and RNS (Scheme 53) [74]. The synthesis of AzuFluor 483-Bpin **157** is accomplished by the palladium-catalyzed borylation of the corresponding 2-amino-6-bromoazulene. The staining of various living cells with compound **157** shows remarkable luminescence due to the conversion to **158** by the reaction with ROS and RNS in the cells. Therefore, compound **157** is expected to apply to the direct detection of ROS and RNS in the living cells.

The same group reported the synthesis of an azulene derivative (NAz-6-Bpin **159**) as a potential fluoride detection agent in water and the evaluation of its ion selectivity (Scheme 53) [75]. The synthesis of NAz-6-Bpin **159** has been achieved by the Sandmeyer reaction of **157** using TMSCl and isoamyl nitrite. Since NAz-6-Bpin **159** can be used in a mixed solvent of water and ethanol without surfactants, this compound has potential applications for the detection of fluoride in drinking water in the field.

## 4. Synthesis of Azulene-Fused Imide Derivatives

Phthalimide and other aromatic imide derivatives have attracted much interest in the fields of materials chemistry and pharmaceutical sciences so that various synthetic methods have been reported.

**Scheme 54:**
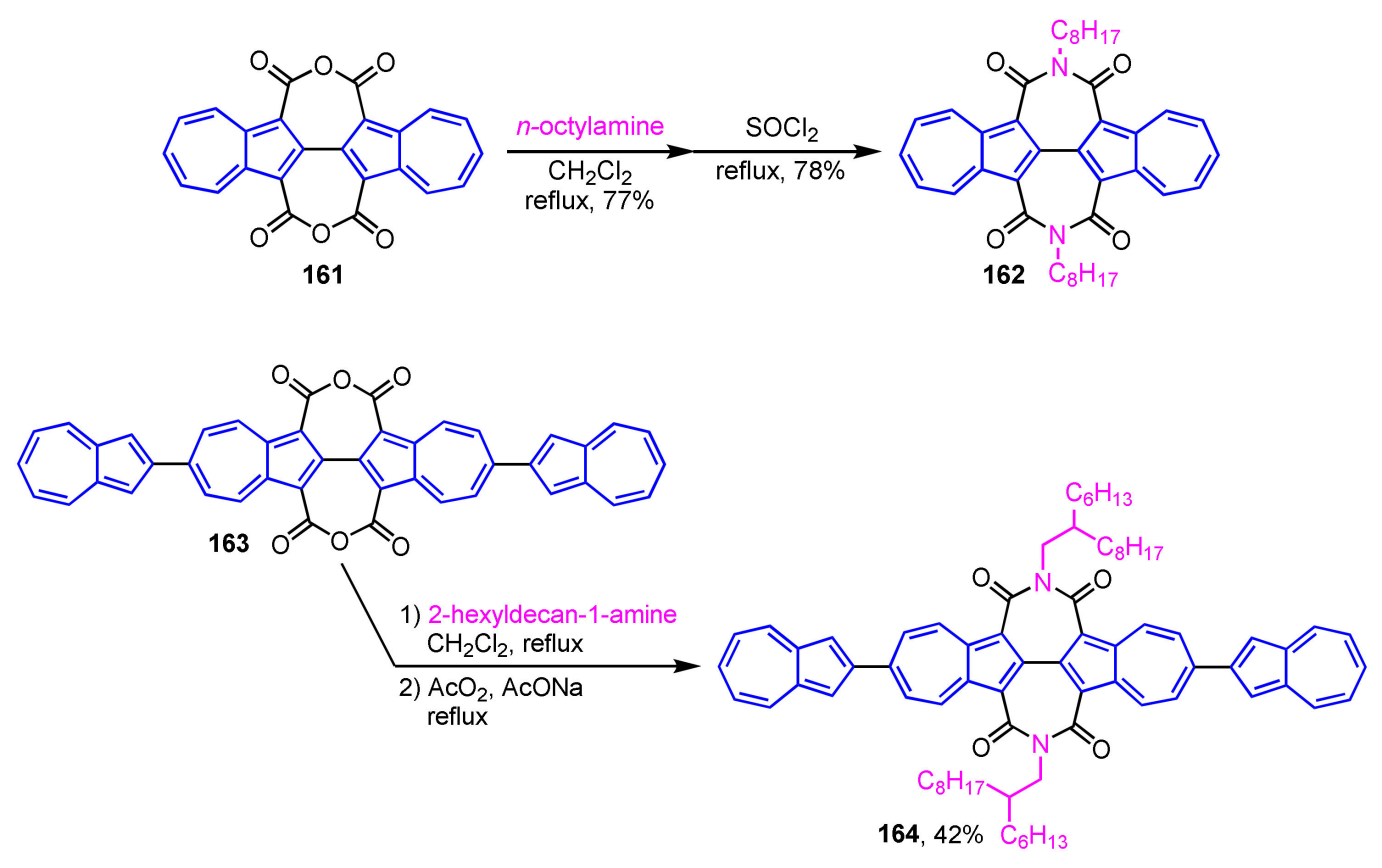
Synthesis of 2,2′-biazulene-1,1′,3,3′-tetracarboxylic diimides (BAZDIs **162** and **164**).

Gao and colleagues have prepared diimide derivatives based on 2,2′-biazulene and demonstrated their optical and electrochemical properties (Scheme 54) [76]. 2,2′-Biazulene-1,1′,3,3′-tetracarboxylic diimides (BAZDIs **162** and **164**) are synthesized by aminolysis of acid anhydrides **161** and **163**, followed by dehydration with SOCl_2_. The X-ray crystal structures, optical and electrochemical properties, and charge transport properties of **162** and **164** are examined. The results of various measurements reveal that BAZDIs **162** and **164** have relatively higher LUMO energies and narrower band gaps than those of the corresponding perylene diimides.

**Scheme 55:**
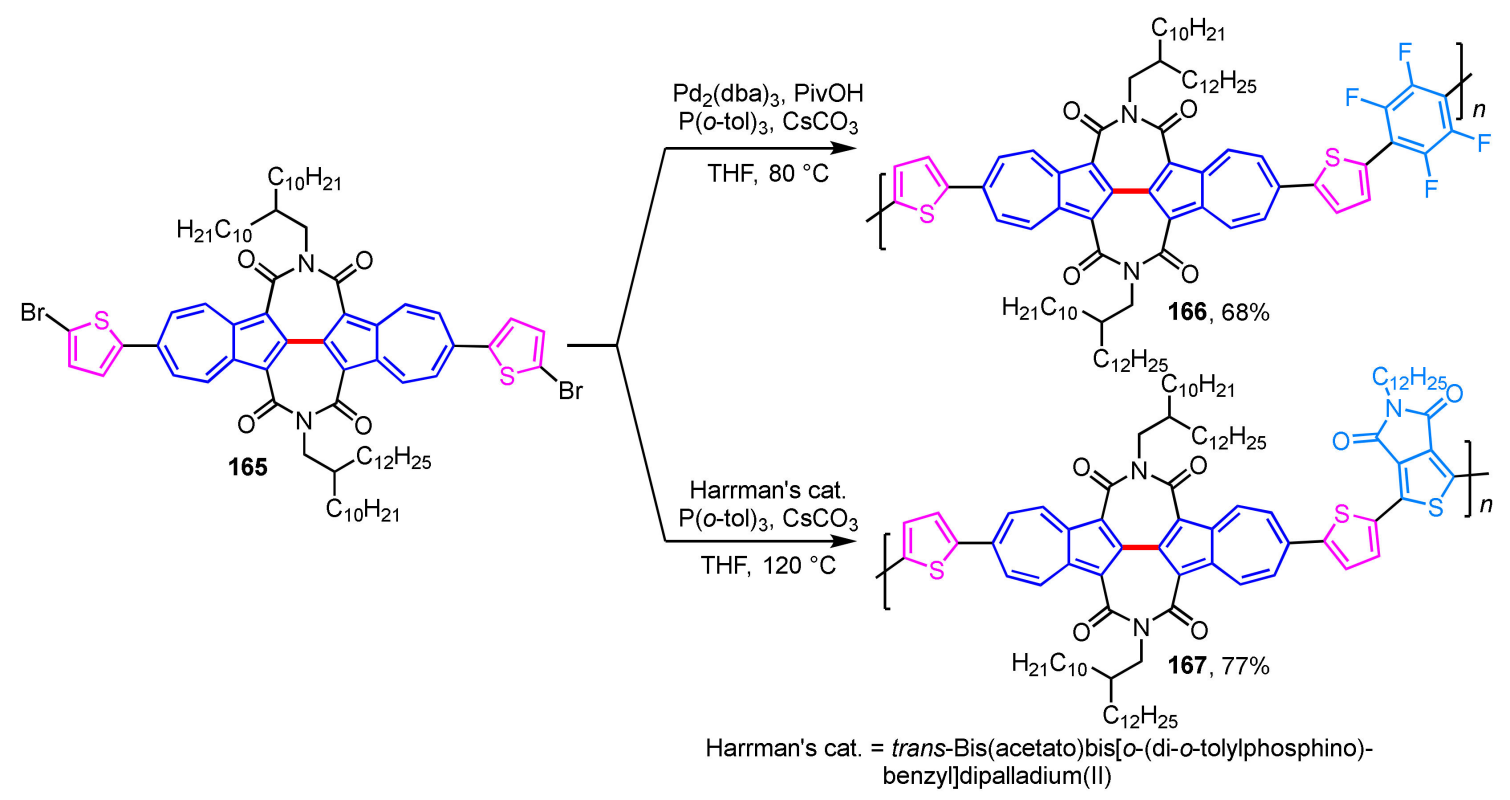
Synthesis of 2,2′-biazulene-based conjugate polymers **166** and **167**.

The same group found that conjugated polymers **166** and **167** embedded with 2,2′-biazulene diimide units act as high-performance OFETs (Scheme 55) [77]. The thermal stability of polymers **166** and **167** is examined by thermogravimetric analysis (TGA), and these polymers did not decompose until the temperature was raised above 400 °C. Conjugated polymers **166** and **167** represent excellent OFET performance with high-electron mobility, and especially polymer **167** was found to be one of the best unipolar *n*-type polymers reported to date.

**Scheme 56:**
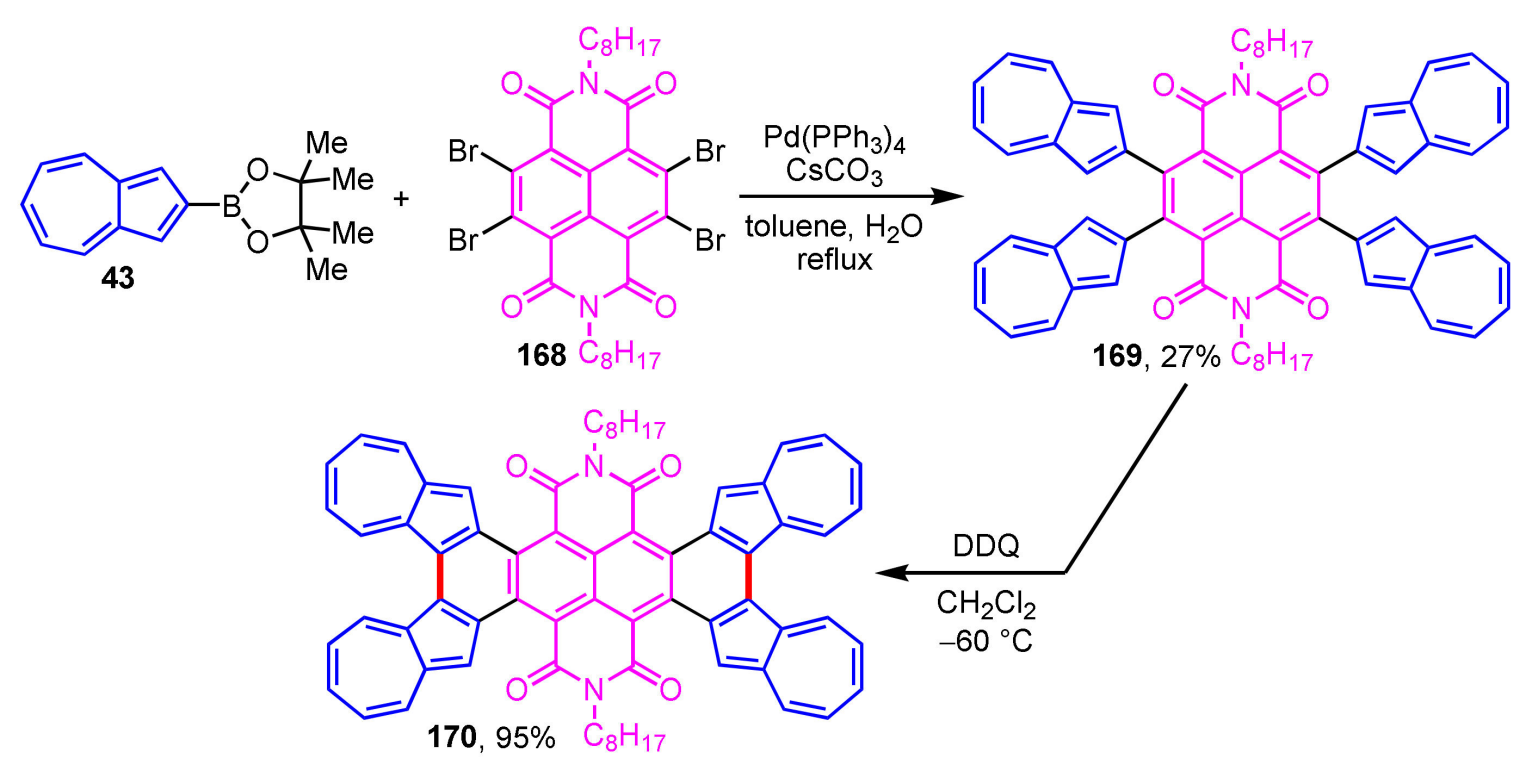
Synthesis of tetraazulene-fused tetracenediimide (TA-fused TDI **170**).

A novel tetraazulene-fused tetracenediimide (TA-fused TDI **170**) was prepared and evaluated using various experimental measurements and theoretical calculations by Tani and co-workers (Scheme 56) [78]. The synthesis of **170** is accomplished by the Suzuki–Miyaura cross-coupling reaction of **43** with tetrabromonaphthalene diimide to give **169**, following the Scholl reaction of **169** with DDQ at low temperature (−60 °C). Fusing azulene rings to the TDI core, **170** shows a small HOMO–LUMO gap and intramolecular CT interaction based on an effective π-electron conjugation. TA-fused TDI **170** also shows unique properties such as absorption in the near-infrared region which appeared in the absorption spectrum, a four-step reversible reduction process observed in the CV experiments, and *n*-type semiconductivity. These outcomes suggest that TA-fused TDI **170** has potential application in photonics as an electronic material.

**Scheme 57:**
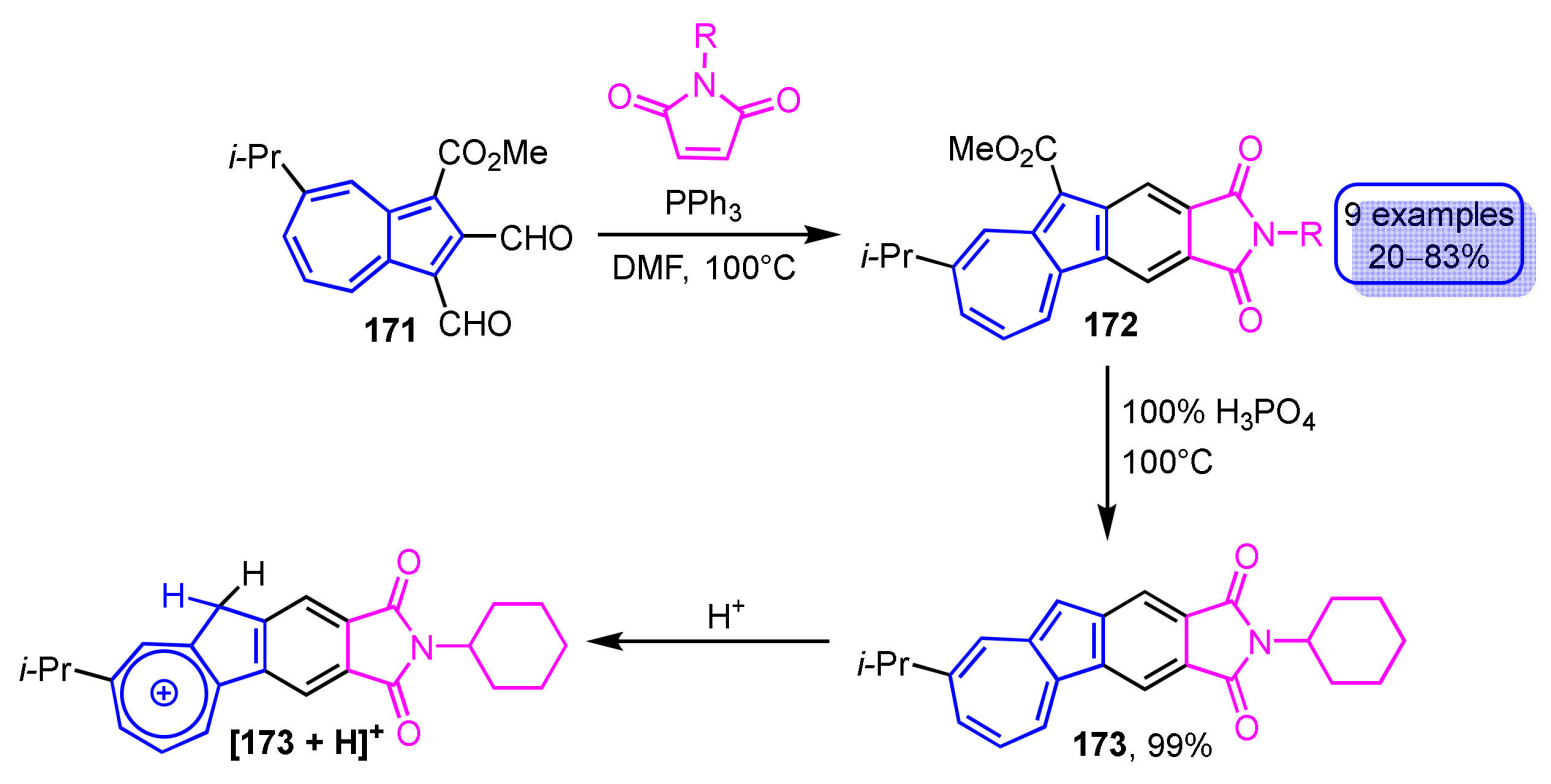
Synthesis and protonation of phthalimide-fused azulene derivative **173**.

Recently, the synthesis of phthalimide-fused azulene derivatives **172** was accomplished by condensation of 1,2-diformylazulene derivatives with maleimides in the presence of phosphine catalysts (Scheme 57) [79]. Various phosphine catalysts and the reaction solvents were investigated to find that phthalimide-fused azulene derivatives **172** were obtained in the highest yields when triphenylphosphine was used as a catalyst in DMF as the solvent. The ester group of **172** can be removed by decarboxylation with 100% H_3_PO_4_, yielding **173** in quantitative yield. The addition of trifluoroacetic acid to the solution of **173** indicates a marked color change, so-called halochromism. Furthermore, blue-colored luminescence is observed when the acidic solution of **173** is irradiated at λ_ex_ = 365 nm, even though no emission is observed in common organic solvents. This luminescence could be ascribed to that from the phthalimide moiety because protonation by trifluoroacetic acid produces a tropylium ion [**173** + H]^+^, which reduces the contribution of the azulene form that may quench the emission.

## 5. Synthesis of Azulene Derivatives Incorporated with Porphyrinoid

**Figure 1:**
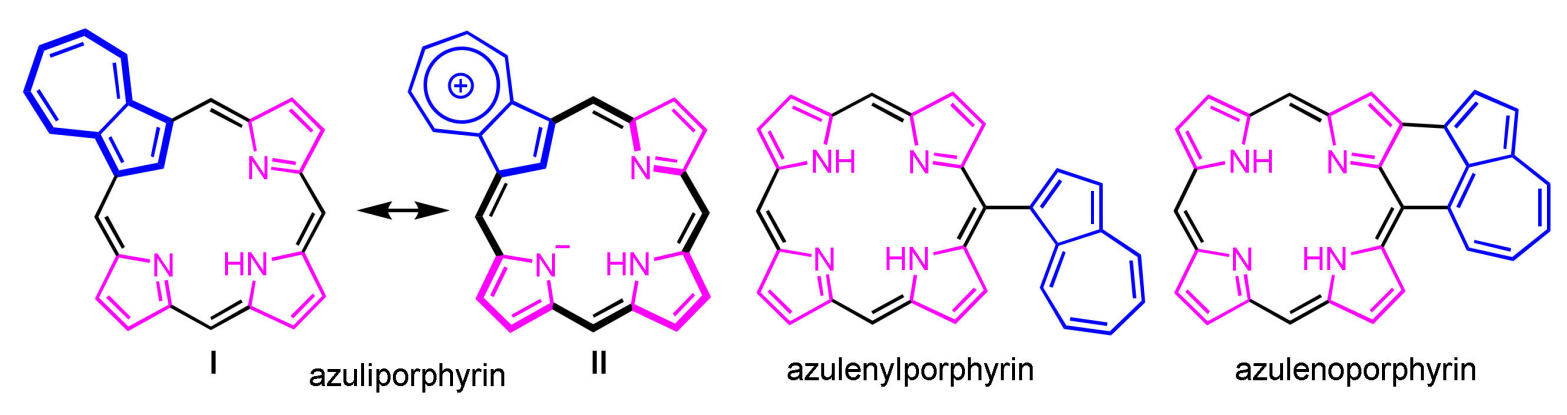
Structures of porphyrinoids incorporated with azulene.

Azulenes with porphyrinoid are classified into three structures as shown in Figure 1. Azuliporphyrin, one of the core-modified porphyrins in which an azulene has replaced a pyrrole subunit, was first reported by Lash in 1997 [80]. Azulene-substituted and azulene-fused porphyrins were reported by Osuka and co-workers [81,82]. There were many reports of azuliporphyrins and related compounds over the last two decades [83,84,85,86]. The azulene subunit interrupts the conjugative pathway, which suggests a nonaromatic character (**I** in Figure 1). However, the zwitterionic resonance structure **II** leads to full macrocyclic aromaticity as an 18π-porphyrinoid. This section surveys the synthetic methods of azuliporphyrin, ring-expanded/contracted azuliporphyrins, and related conjugated compounds.

**Scheme 58:**
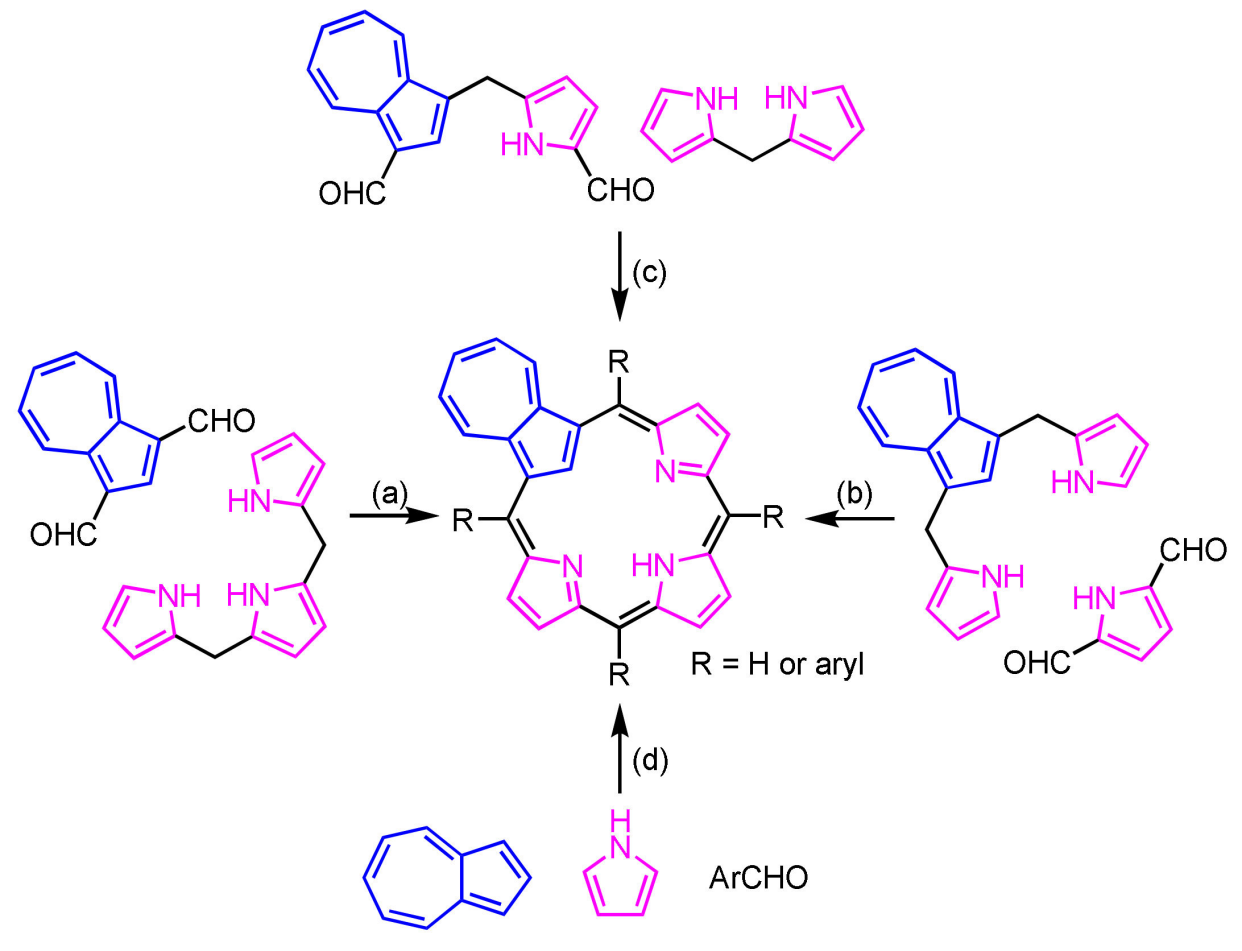
Construction of azuliporphyrin frameworks.

Azulene (**1**) favors the electrophilic substitution reaction at the 1,3-positions, which structurally resemble pyrrole at the α-positions. Thus, azulene (**1**) is used as an ideal precursor for the construction of carbaporphyrinoid. The azuliporphyrin skeleton is constructed via the following routes: (a) [3 + 1] MacDonald condensation [80], (b) back-to-front [3 + 1] strategy [87], (c) [2 + 2] MacDonald condensation [88] and (d) Lindsey–Rothemund-type condensation [89] (Scheme 58).

**Scheme 59:**
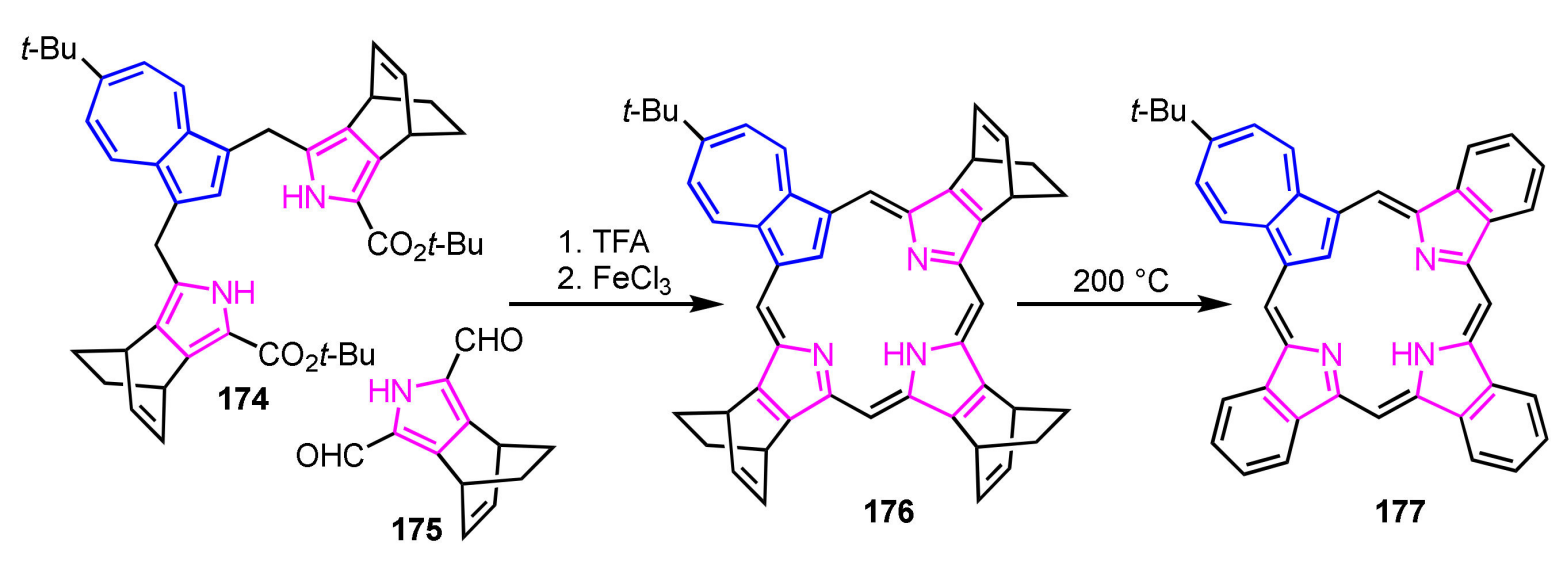
Synthesis of tribenzoazuliporphyrin **177** via retro Diels–Alder reaction.

Back-to-front [3 + 1] synthesis afforded various azuliporphyrin derivatives by taking advantage of stable azulitripyrrane. This method was adopted for the preparation of mono-, di-, and tribenzoazuliporphyrins [90]. Bicyclo[2.2.2]octadiene-fused azuliporphyrin **176** was prepared by condensation of azulitripyrrane **174** with 2,5-diformylpyrrole **175**, followed by oxidation with FeCl_3_ before tribenzoazuliporphyrin **177** was quantitatively obtained via retro Diels–Alder reaction (Scheme 59).

**Scheme 60:**
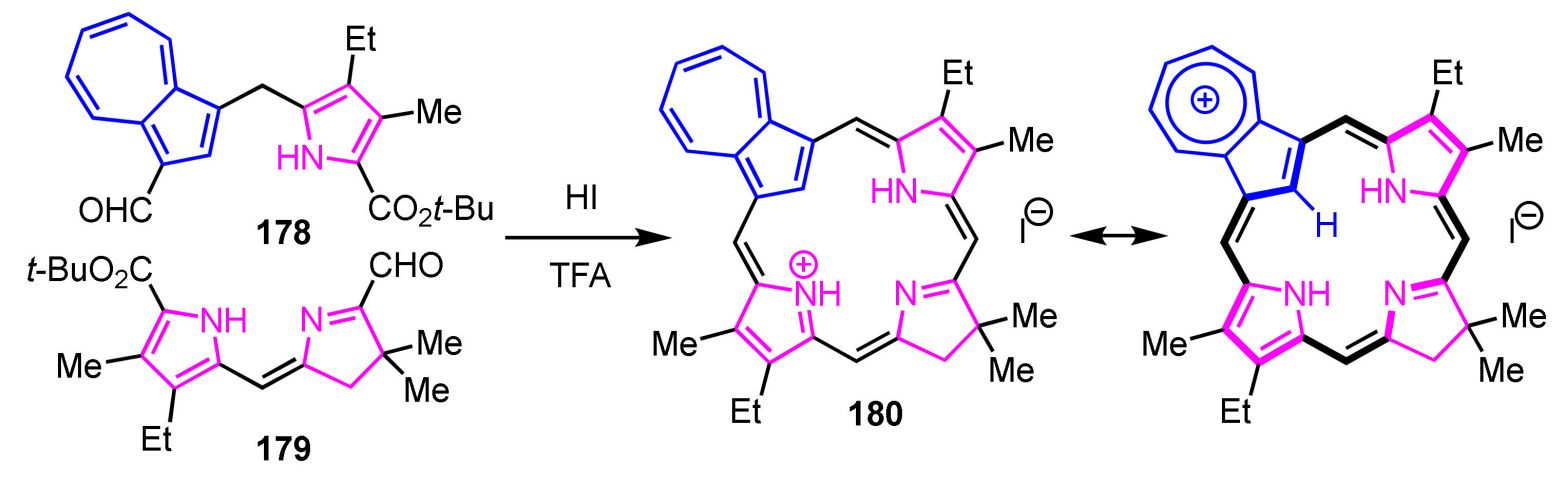
Synthesis of protonated azulichlorin **180**.

Lash and co-workers reported azulichlorins via [2 + 2] condensation. Azulidipyrrane **178** and dihydrodipyrrin **179** were treated with TFA to remove the *tert*-butyl ester groups. After dilution with acetic acid and addition of HI, the reaction mixture was stirred at room temperature overnight. The protonated azulichlorin **180** was obtained, resulting from the cross-condensation in 17% yield (Scheme 60) [91]. In this reaction, the self-condensation products were not obtained. The NMR spectrum of **180** shows the internal CH at −2.11 ppm and *meso*-CHs at 7.5–9.3 ppm, which indicates that the cation **180** has a similar diamagnetic ring current to protonated azuliporphyrins due to the contribution of resonance structure of the 18π-electron system as shown in Scheme 60.

**Figure 2:**
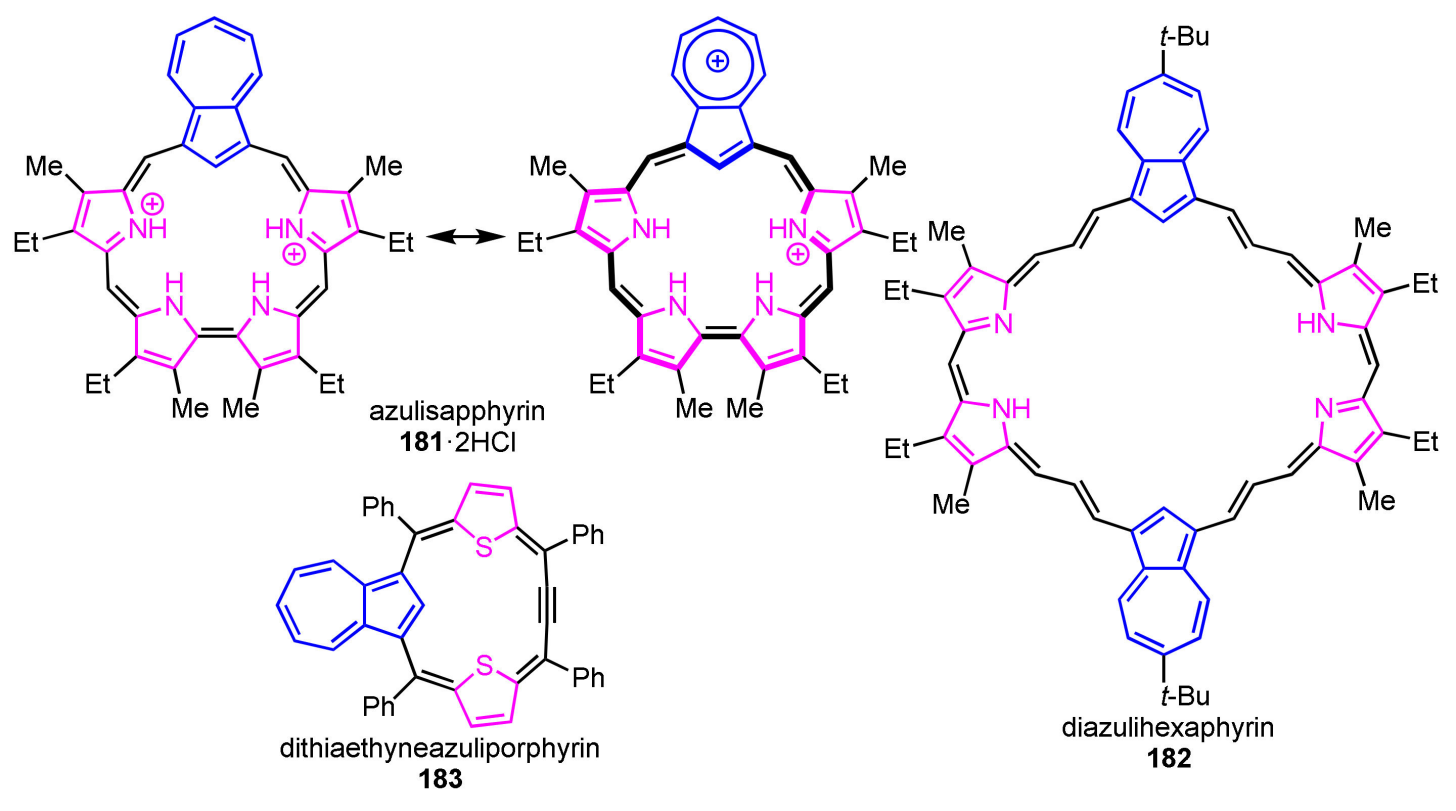
Reported ring-expanded/contracted azuliporphyrins **181**–**183**.

There are few ring-expanded/contracted azuliporphyrinoids while a variety of azuliporphyrins were reported (Figure 2). Lash and co-workers reported a [4 + 1] route to azulisapphyrin with a 22π-system [92]. Diazulihexaphyrin(3.3.1.3.3.1) was reported under [2 + 1] condensation between a dipyrromethane and an azulene bisacrylaldehyde, which did not show macrocyclic aromaticity [93]. Latos-Grażyński and co-workers reported dithiaethyneazuliporphyrin with a nonaromatic triphyrin(4.1.1) framework [94].

**Scheme 61:**
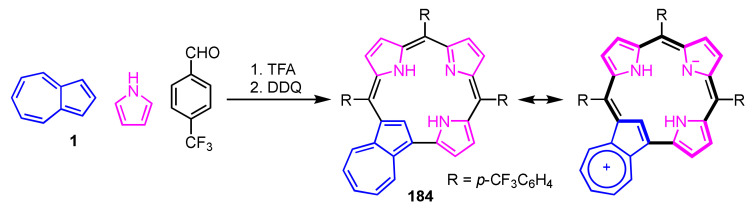
Synthesis of azulicorrole **184**.

Ghosh and co-workers reported azulicorrole as the first carbacorrole except for *N*-confused corroles in 2019 [95,96]. Azulicorrole **184** was isolated via acid-catalyzed condensation of azulene, pyrrole and *p*-trifluoromethylbenzaldehyde with a molar ratio of 1.5:4.5:2.5, followed by oxidation with DDQ (Scheme 61). Although the yield was poor, free base **184** was readily converted into the Cu(III) and Au(III) complexes. The NMR spectrum of **184** shows signals of the internal CH at 3.19 ppm and NHs at 3.47 ppm, indicating that **184** has a lower diatropic ring current compared to triarylcorroles.

**Scheme 62:**
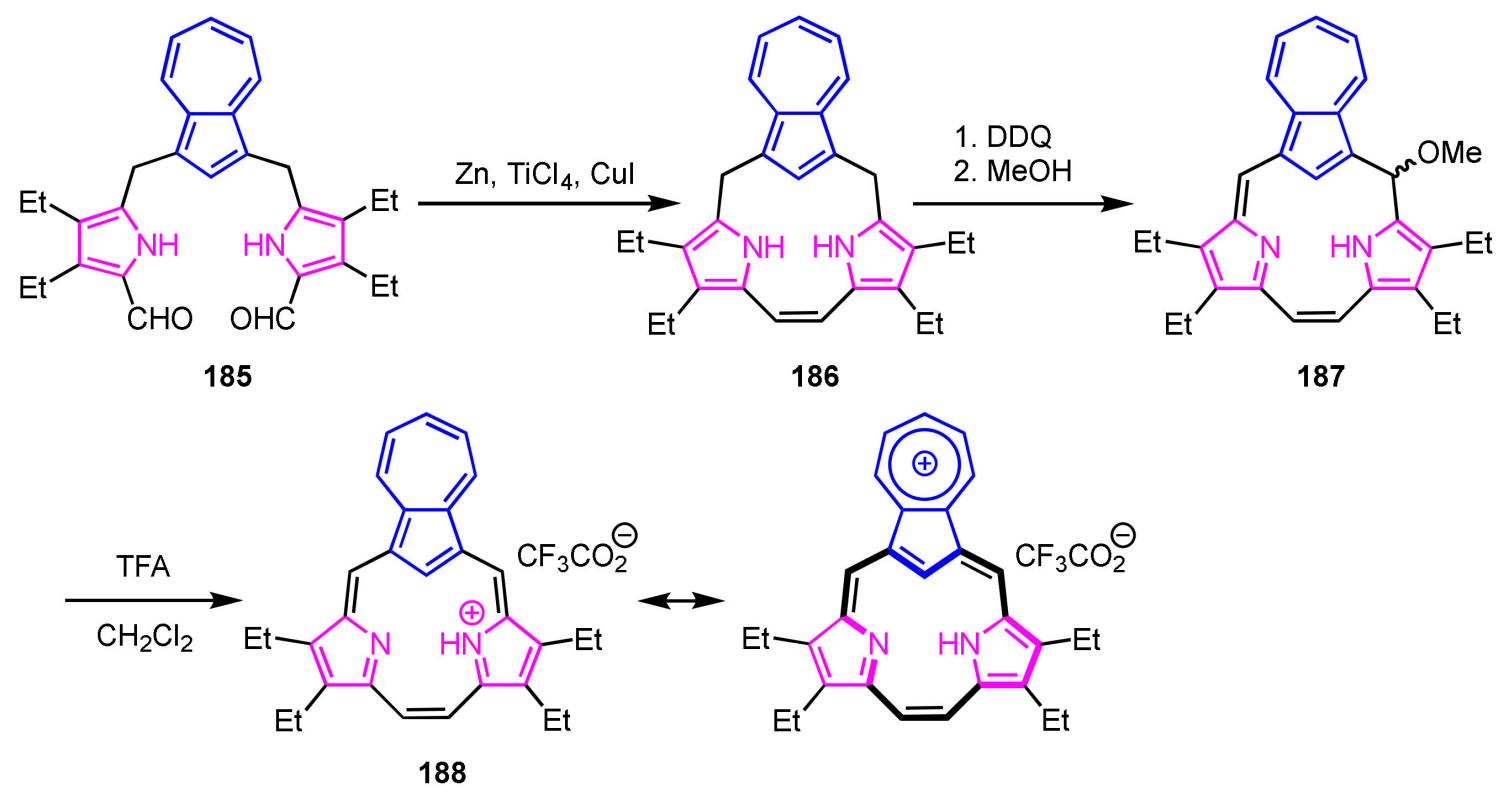
Synthesis of azulitriphyrin **188** with triphyrin(2.1.1) framework.

Okujima et al. [97] and Lash et al. [98] subsequently reported azulitriphyrin with triphyrin(2.1.1) framework. Intramolecular McMurry coupling of diformyltripyrrane is a key step for the construction of the triphyrin(2.1.1) framework (Scheme 62) [99]. Tetrahydroazulitriphyrin was obtained via McMurry coupling of **185**. Oxidation with DDQ and treatment with TFA afforded protonated azulitriphyrin **188** as a trifluoroacetate. The NMR spectrum of **182** shows signals of the internal CH at 2.53 ppm and NHs at 10.86 ppm, which are upfield-shifted compared to those of **187** due to global diatropic ring current. The NICS calculations also supported that azulitriphyrin **188** is a 14π-electron aromatic system.

**Scheme 63:**
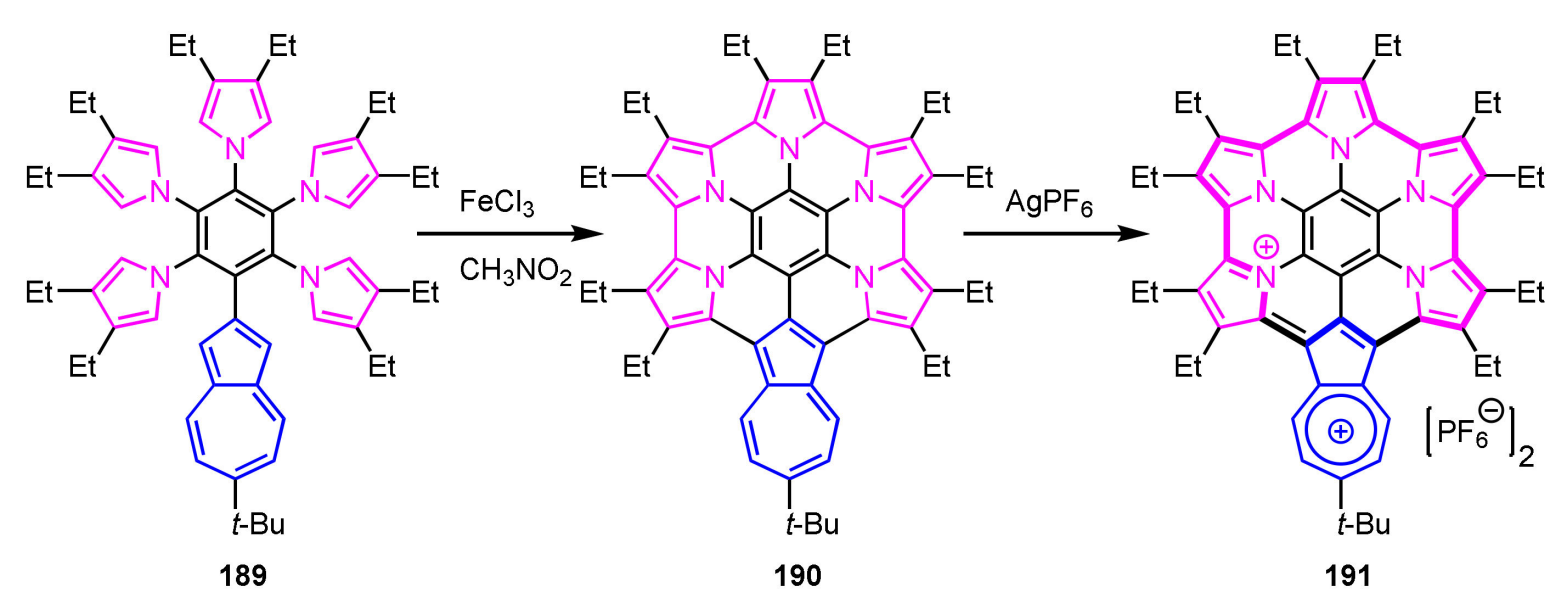
Synthesis of azulene-fused azacoronene analogue **190**.

In 2019, Takase, Uno, and co-workers reported azulene-fused azacoronene analogue **190** (Scheme 63) [100]. Suzuki–Miyaura cross-coupling reaction of bromopentafluorobenzene with the corresponding azulenylboronate, followed by the nucleophilic aromatic substitution with 3,4-diethylpyrrole, gave 2-azulenylpentapyrrolylbenzene **189**. Azulene-fused azacoronene **190** was obtained via the Scholl reaction with FeCl_3_ in a moderate yield. This azacoronene showed reversible multistep-oxidations similar to hexapyrrolohexaazacorone [101]. Dication **191** was isolated via oxidation of **190** with AgPF_6_ or SbCl_5_ and showed a 22π-electron conjugation with azulihexaphyrin(0.0.0.0.0.0) framework confirmed by the crystal analysis and the NICS and ACID calculations.

## 6. Conclusions

In this review, we summarize the preparation, reactivity, and physical properties of heterocycle-substituted and heterocycle-fused azulene derivatives that have been reported in the last decade. There are a wide variety of methods for their synthetic approach and the physical properties of the azulene derivatives are highly dependent on the type of heterocycles and their substitution and fused positions.

**Scheme 64:**
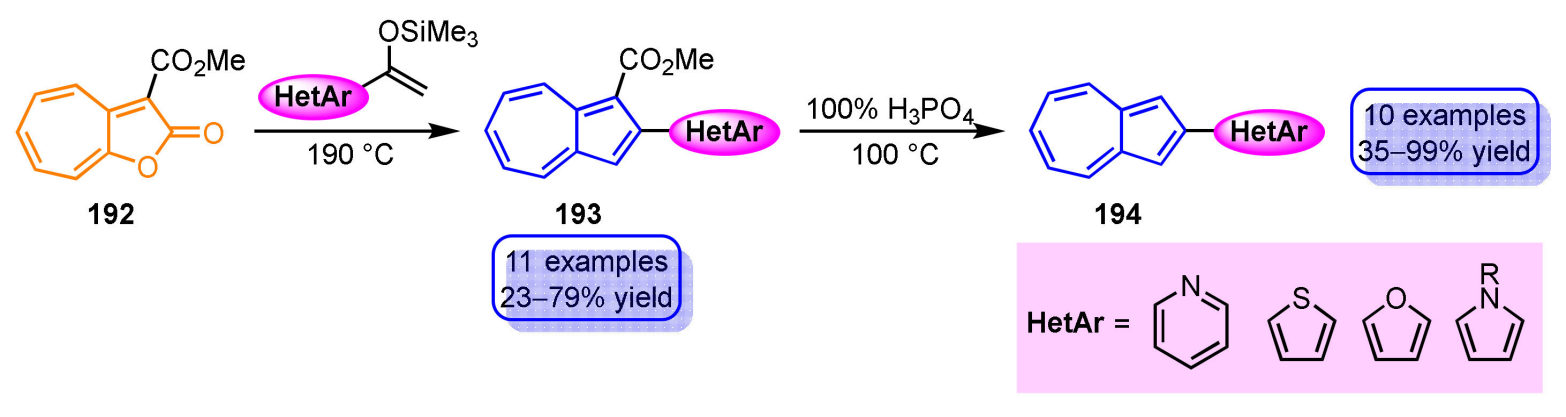
Synthesis of 2-heteroarylazulenes **190**.

1-Heteroaryl- and 1,3-bis(heteroaryl)azulene derivatives have been prepared by various methods, including electrophilic substitution, cyclization, radical coupling, and transition metal-catalyzed cross-coupling reactions, whereas the introduction of a heterocycle to the 2- and 6-positions of azulene ring is mostly achieved by cross-coupling reactions using the corresponding haloazulenes as a starting material because of the low reactivity of electrophiles to these sites. There is only one example of electrophilic substitution reaction by heterocyclic compound at the 2-position of the azulene ring, but in this case, a strong electron-donating group such as a dimethylamino group at the 6-position and protection at the 1,3-positions of the azulene ring are both essential. Recently, the reaction of 2*H*-cyclohepta[*b*]furan-2-one derivatives **192** with heterocycle-substituted silyl enol ethers have been reported as a new synthetic method for 2-heteroarylazulenes without using cross-coupling reactions (Scheme 64) [102]. This method is applied to the synthesis of azulenes with various heterocycles such as 2-(pyridyl-, thienyl-, furyl-, and pyrrolyl)azulenes **193**, although the reaction requires high temperature (190 °C). Furthermore, the ester group of **194** can be removed by decarboxylation with 100% H_3_PO_4_.

The synthesis of azulene derivatives with heterocyclic substitutions at the 4-, 5-, and 6-positions can be accomplished by a variety of methods, such as Zieglar–Hafner method, aromatic nucleophilic substitution, and cross-coupling reactions.

The synthetic methods described in this review are extremely beneficial for the preparation of novel heterocycle-substituted and heterocycle-fused azulene derivatives that have growth potential as organic electronics and pharmaceuticals.
